# Muscular fatigue in response to different modalities of CrossFit sessions

**DOI:** 10.1371/journal.pone.0181855

**Published:** 2017-07-28

**Authors:** José Luis Maté-Muñoz, Juan H. Lougedo, Manuel Barba, Pablo García-Fernández, Manuel V. Garnacho-Castaño, Raúl Domínguez

**Affiliations:** 1 Department of Physical Activity and Sport Sciences, Alfonso X el Sabio University, Madrid, Spain; 2 TecnoCampus, College of Health Sciences, Pompeu Fabra University, Mataró-Maresme, Barcelona, Spain; Universitat de les Illes Balears, SPAIN

## Abstract

**Background:**

CrossFit is a new strength and conditioning regimen involving short intense daily workouts called workouts of the day (WOD). This study assesses muscular fatigue levels induced by the three modalities of CrossFit WOD; gymnastics (G), metabolic conditioning (M) and weightlifting (W).

**Material and methods:**

34 healthy subjects undertook three WOD (one per week): a G WOD consisting of completing the highest number of sets of 5 pull-ups, 10 push-ups and 15 air squats in 20 min; an M WOD, in which the maximum number of double skipping rope jumps was executed in 8 sets (20 s), resting (10 s) between sets; and finally, a W WOD in which the maximum number of power cleans was executed in 5 min, lifting a load equivalent to 40% of the individual's 1RM. Before and after each WOD, blood lactate concentrations were measured. Also, before, during, and after each WOD, muscular fatigue was assessed in a countermovement jump test (CMJ).

**Results:**

Significant reductions were produced in the mechanical variables jump height, average power and maximum velocity in response to G; and in jump height, mean and peak power, maximum velocity and maximum force in response to W (*P*<0.01). However, in M, significant reductions in mechanical variables were observed between pre- and mid session (after sets 2, 4, 6 and 8), but not between pre- and post session.

**Conclusions:**

Muscular fatigue, reflected by reduced CMJ variables, was produced following the G and W sessions, while recovery of this fatigue was observed at the end of M, likely attributable to rest intervals allowing for the recovery of phosphocreatine stores. Our findings also suggest that the high intensity and volume of exercise in G and W WODs could lead to reduced muscular-tendon stiffness causing a loss of jump ability, related here to a longer isometric phase during the CMJ.

## Introduction

CrossFit is a new fitness regimen consisting of short, high-intensity daily workouts [1]. Since its development in the 1990's, it has become a competition sport with close to 11,000 affiliates in gymnasiums, and the over 200,000 athletes participating worldwide in 2014 [2]. CrossFit is described by its founder as a physical strength and conditioning program based on functional movements such as weightlifting, gymnastics and metabolic conditioning [3]. Training is organized as daily sessions designated workouts of the day or WOD. Exercises differ widely and are constantly varied. According to the contents of the workouts, there are three modalities: gymnastics (G), metabolic conditioning (M) and weightlifting (W) [4, 5].

The gymnastics modality comprises body weight exercises designed to improve body control and include for example bodyweight squats, push-ups, pull-ups, rope climb, rings or parallel bars. Metabolic conditioning movements offer little resistance and are designed to generate fatigue. Exercises may be aerobic or anaerobic and sessions are organised as interval training (bouts of high intensity work interspersed with rest periods). Examples are running, rowing, jump rope, and even swimming or cycling. The weightlifting modality comprises external load exercises including functional power lifts such as squat or deadlift, Olympic lifts such as the snatch, clean and jerk, or other lifts such as overhead press using kettlebells, sandbags, med-balls, etc. [6]. The purpose of some of these exercises is to achieve the best time possible while for others the goal is the largest number of rounds over periods in the range of 10 to 20 minutes [6].

Despite CrossFit having been described to induce muscular injuries including rhabdomyolysis, there is scarce scientific evidence linking this form of training to this risk [7]. In effect, CrossFit has been related to similar risks of injury to those described for power lifting, gymnastics or contact sports such as rugby [8]. However, given that many CrossFit exercises are technically demanding and require high power outputs sustained over time, they could provoke considerable fatigue and lead to injury in subsequent exercise sessions. In effect it has been observed that fatigue modifies movement biodynamics [9].

Muscular fatigue is defined as the incapacity of the neuromuscular system to produce energy around a joint [10]. Among the mechanisms giving rise to fatigue is the inhibition of muscle contraction due to the build-up of end metabolic products such as lactate [11]. Increased lactate concentrations diminish the contractile capacity of muscle [12] because of both the accumulation of hydrogen ions reducing pH and generating metabolic acidosis and the inhibition of phosphofructokinase (PFK). Muscular fatigue reflected by mechanical variables (force, velocity and power output), is the outcome of the impaired contractile properties of muscle or of their neuromuscular control [13]. One of the methods most used to quantify neuromuscular fatigue in terms of given mechanical variables is to calculate the muscle's loss of capacity to generate power following exercise in the countermovement jump test (CMJ) [14, 15].

Possible mechanisms that may produce fatigue during Crossfit WOD have been investigated in a single study examining the effects of one and two consecutive Crossfit sessions on metabolic responses (lactate and blood glucose), muscle power production and cytokine levels (IL-6, IL-10 and osteoprotegin) [16]. Thus, given the high technical demands of some CrossFit exercises along with the known effects of fatigue on movement biodynamics, this study was designed to examine fatigue levels induced by the three CrossFit workout modalities (G, M and W) by assessing metabolic responses (blood lactate concentrations) and CMJ jump performance losses. Our ultimate aim was to identify the WOD inducing most fatigue so that programs could be designed to avoid excessive risks of muscle injury.

## Materials and methods

### Experimental approach to the problem

Four exercise sessions were executed by each study participant on the same day of the week and within the same time frame (±3 hours). Each session was separated by 1 week of rest. Before arrrival at the laboratory, participants were informed about the exercises they were to undertake on that day. The first exercise session consisted of a gymnastics WOD and the second session of a metabolic conditioning WOD. In the third session, the load to lift in the fourth session was individually determined in an incremental weightlifting power clean test to give the maximal strength for this exercise. Accordingly, once the load to be used in the power clean test had been determined, the final WOD was the weightlifting WOD. All sessions were conducted under the same ambient conditions (temperature 21–25°C, atmospheric pressure 715–730 mm Hg, relative humidity 40–50%). An external investigator supervised each WOD to ensure the correct completion of each movement. All CrossFit sessions were conducted at the Exercise Physiology and Sports Training laboratory of our university. This spacious facility is well equiped for this type of exercise.

### Subjects

The study participants were 34 young, healthy men, all students of the Physical activity and Sport Sciences degree course. Descriptive data for these subjects are provided in Table 1. Inclusion criteria for participant selection were at least 6 months of experience in strength training routines including free weight lifts and Olympic lifts and the non use of medication or performance enhancing drugs during the study. None of the participants had experience with CrossFit WOD and elite athletes were excluded. These data were collected in a questionnaire completed before the study start.

**Table 1:** Anthropometric characteristics of the 34 study participants.

Variable	M ± *SD*
Age (years)	22.03 ± 3.1
Weight (kg)	76.90 ± 7.1
Height (cm)	178.65 ± 0.6
BMI (kg·m^2-1^)	23.64 ± 1.7

BMI = body mass index; G = Gymnastics session; M = Metabolic conditioning session; W = Weightlifting session; M ± SD = Mean (± standard deviation).

Subjects were instructed to refrain from any physical exercise in the 48 hours before each exercise session.

Before the study outset, the exercises and tests participants would have to undertake were explained to them and voluntary, written informed consent was obtained from each one. The study protocol adhered to the tenets of the Declaration of Helsinki and was approved by the ethics committee of the Department of Physical Activity and Sport Sciences of the Universidad Alfonso X El Sabio.

### Session 1: Gymnastics WOD

The session started with a warm-up consisting of 5 min of low intensity running and 5 min of joint mobility and dynamic stretching exercises. The gymnastics WOD was the “Cindy” protocol [2, 17,18] which involves executing the greatest number of sets of 5 pull-ups, 10 push-ups and 15 air squats (bodyweight squats) in 20 min. Each repetition of each exercise had to be completed to continue on to the next round, and each exercise had to be properly executed according to preestablished minimum standards. One of the authors was responsible for counting rounds using a hand held counter. The techniques used for each exercise were as follows:

#### Pull-up

The starting position is hanging from a bar with the elbows fully extended and hands in pronation separated by a distance wider than the hips. From this position, the body is lifted in one movement by bending the elbows and raising the shoulders until the chin is higher than the bar. No kipping pull ups or butterfly pull ups were allowed as the study participants were insufficiently experienced with these movements.

#### Push-up

The starting position is elbows fully extended and hands on the floor directly under the shoulders, with feet together also touching the floor and the trunk and legs fully stretched. From this position, the elbows are flexed until just touching the floor with the chest and then immediately extended again.

#### Air squat

Starting with knees and hips straight, arms crossed over chest, feet at shoulder width and toes pointing slightly outwards, the subject flexes the knees and hips until 120°, and from this position returns to the fully extended starting position.

### Session 2: Metabolic conditioning WOD

Subjects undertook the same warm-up as in session 1. The exercise selected for the M modality WOD was double skipping rope jumps (CrossFit double unders) as high-intensity interval training (HIIT). For this exercise, the subject undertakes a high vertical jump and passess the skipping rope two times under the feet. The WOD consisted of conducting the maximum number of double unders possible in 8 sets of 20 s with 10 s of rest between sets. This intermittent training protocol is based on the findings of Tabata et al. (1996) [19]. Test duration was 4 min. In this WOD, an observer counted the number of double unders completed per set while another observer checked the work and rest times.

### Session 3: Power clean incremental load test

In the third session, the weights to be lifted by each subject in the weightlifting WOD the following week (session 4) were calculated (40% 1RM). Subjects performed an incremental load power clean test to determine each individual's maximum strength or 1RM.

Mean barbell velocity measurements were made using the linear position transducer Tendo Weight-lifting Analyzer System (Trencin, Slovak Republic), which has been recently validated [20].

The general warm-up was as described for the previous sessions. Specific warm-up consisted of 2 sets of 3 power clean repetitions using a 20 kg load. Then, after 3 min of rest, the test was started.

Using an initial load of 20 kg subjects executed one power clean repetition increasing the load by 10 kg in each repetition provided mean barbell velocity was higher than 1.5 m·s^-1^, with an inter-repetition rest period of 3 min. When mean barbell displacement velocity was less than 1.5 m·s^-1^, load increases were reduced to 5 kg and rest periods lengthened up to 5 min. The test ended when subjects reached their 1RM, that is, the maximum load lifted via a correct exercise technique [21].

One week before the first session, each participant was able to practice the power clean with the help of a qualified weightlifting trainer.

### Session 4: Weightlifting WOD

Subjects performed the same general and specific warm up as for the G and M sessions. In the W session, the maximum number of power cleans (Olympic lifts) was executed lifting a load equivalent to 40% of the individual's 1RM determined in session 3. Session duration was 5 minutes. The barbell used for the power clean weighed 20 kg and to this weight the necessary discs were added to obtain the weight equivalent to each subject's 40% 1RM. One of the authors was responsible for counting the total number of power cleans completed in the 5 min available.

### Blood lactate

Before the onset and at the end of each CrossFit session, the same operator took a blood sample (5 μl) by finger pricking for lactate determination. Blood lactate concentrations were measured in these samples using a portable analyzer validated for this purpose Lactate Pro LT-1710 (Arkray Factory Inc., KDK Corporation, Siga, Japan) [22, 23].

### Muscular fatigue

Muscular fatigue was measured in the lower limbs during a CMJ [24] performed on a portable, 92 x 92 x 12.5 cm force platform (Quattro Jump model 9290AD; Kistler Instruments, Winterthur, Switzerland). The jump was initiated while standing on the platform with legs extended and hands on hips. For the jump, the legs are first flexed to 90° (eccentric action) and then explosively extended in a coordinated manner (concentric action) trying to reach maximum height. During the flight stage, the knees should be extended. Contact with the ground is made with the toes first. During the test, subjects were instructed to keep their hands on their hips and avoid any sideways displacements during the flight stage.

**Fig 1:**
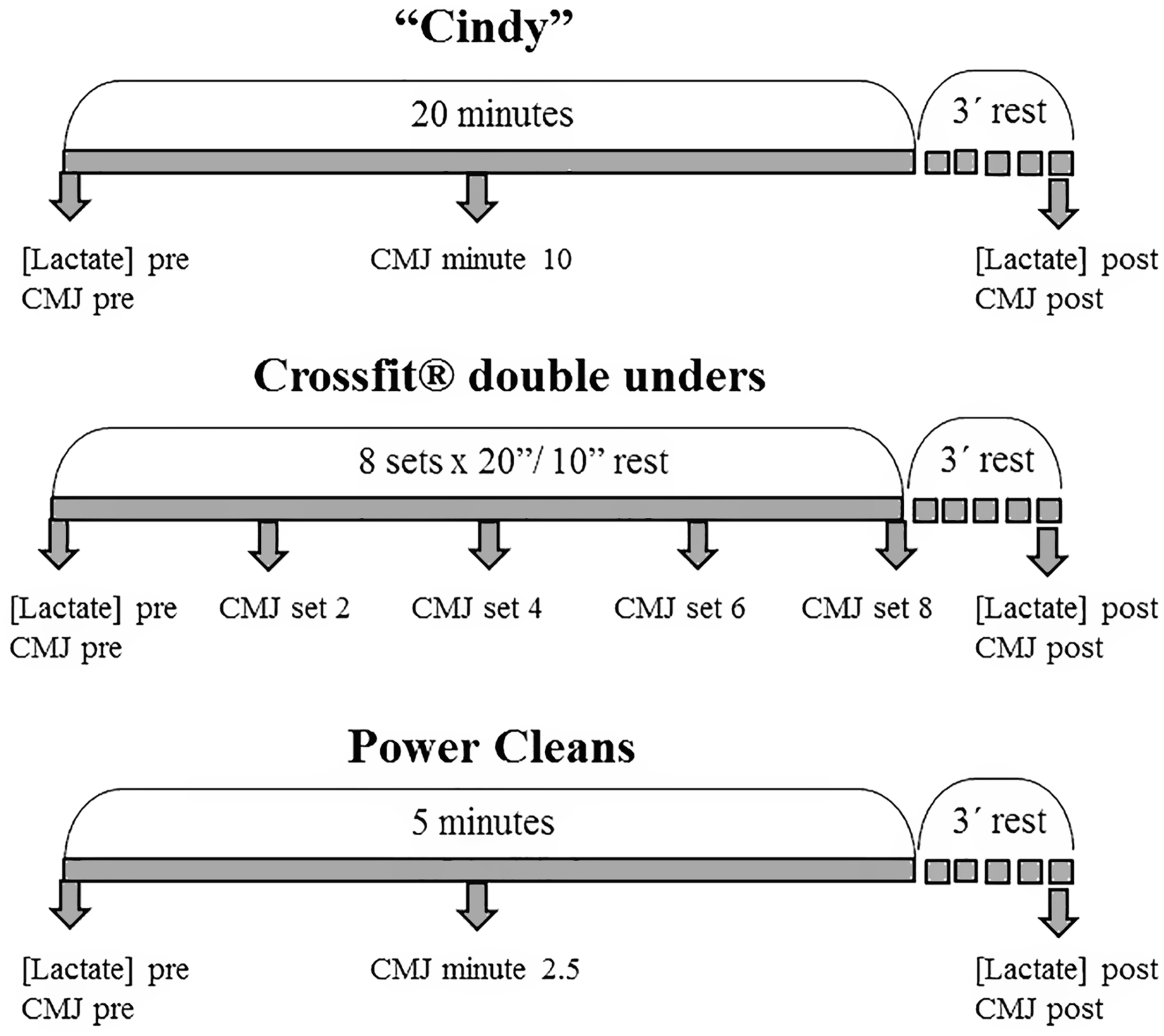
Three modalities of CrossFit WOD; Gymnastics (Cindy), Metabolic conditioning (Crossfit double-unders) and Weightlifting (Power Cleans). The figure shows the working times and [Lactate] and Countermovement Jump measurements in each Crossfit WOD.

This test was performed before, during and after executing each of the different WOD. In all the WOD modalities, the initial and final jump tests were performed after warm up and 3 min after session completion, respectively. In these tests three jumps were executed separated by 30 s rest periods. For the intrasession CMJ tests, one jump was performed at minutes 10 and 2.5 in the G and W workouts respectively and after sets 2, 4, 6 and 8 in the M sessions. The objective of the jump measurements was to assess jump ability throughout the sessions and 3 min after finishing the sessions (Fig 1). To interfere as little as possible with the WOD, one of the observers was prepared to complete the CMJ test in as short a time possible. In effect, intrasession jump test duration was around 3–4 seconds. In the M WOD, it was made sure that the 10-s rest periods were uninterrupted.

The CMJ measurements recorded were jump height (H), average power relative (APR), average power total (APT), peak power relative (PPR), peak power total (PPT), maximum takeoff velocity (Vmax), maximum force (Fmax), peak rate of velocity development (RVD_peak_), peak rate of force development (RFD_peak_), total jump duration and duration of the eccentric (ECC), isometric (ISO) and concentric (CON) phases.

These variables were selected because a loss of jump height and force in a CMJ test during an exercise session has been used to indicate neuromuscular fatigue [14]. Other variables that have been used to assess jump ability and neuromuscular fatigue are total jump duration, and the duration of the eccentric and concentric phases [25, 26]. In our study, these durations were determined by measuring vertical reaction forces during the jump (range 0–10 kN; sampling frequency 0.5 kHz). The force platform was connected to a PC and the software package of Kistler (Quattro Jump software, version 1.1.1.4) was used to quantify the variables. During the complete jump, this software measures time in seconds (X axis) and force expressed as subject body weight (Y axis). Accordingly, when the participant stood still on the platform, the force was 1. The jump started when an abrupt drop in this value of 1 (equivalent to the weight of the still subject), and this was also the onset of the ECC phase. As reference, the first value greater than—0.01 over 1, was used to quantify the jump start. The jump finalized when the subject stopped contacting the platform (X axis = 0). The final stage of ECC corresponded to the first force peak which almost always coincides with RFD_peak_. The end of the ECC stage determines the start of the ISO stage, which continues until a difference greater than 0.009 is recorded (in relation to the value 1 of body weight) in the forces column (Y axis). Finally, CON was taken from the time when ISO finishes until the moment the individual stops making contact with the platform (X axis = 0).

### Statistical analysis

Initially the Shapiro-Wilk test was used to check the normality of data. To compare [lactate] in the different CrossFit WOD, two factor ANOVA tests were conducted: one with repeated measures, that is, an inter-subject factor, or group effect (3 levels G, M and W) and one with an intra-subject factor (2 levels pre- and postexercise). The effects of their interaction were also assessed.

To quantify muscular fatigue during the three CrossFit modalities, repeated measures ANOVA was performed for the factor time, contrasting it using the Mauchley sphericity test. When the hypothesis of sphericity was rejected, the univariate *F* statistic was used adjusting it with the correcting index Greenhouse-Geisser. When significant differences between measurements were detected, the post-hoc Bonferroni test was used.

All data were expressed as the M (mean) and SD (standard deviation). In all tests, effect size (*ES*) and the statistical power (SP) of the data were determined. Percentage gains were calculated using the formula ([post-pre]/pre X 100). Significance was set at *P*<0.05. All tests were performed using the SPSS package for Windows, version 18.0 (SPSS, Chicago, III).

## Results

Table 1 provides demographic data for the study participants (S1 File). Performance indicators are shown in Table 2 as the number of rounds executed in the gymnastics WOD, the number of double unders completed in each set of the metabolic WOD and the number of lifts performed in the 5 min weightlifting WOD.

**Table 2:** Performance data recorded in the three CrossFit WOD.

Variable	M ± *SD*
No. rounds in G	19.59 ± 2.6
No. double unders in M:	
Set 1	13.85 ± 7.7
Set 2	12.76 ± 7.2
Set 3	11.71 ± 6.5
Set 4	9.62 ± 4.9
Set 5	10.65 ± 5.7
Set 6	9.88 ± 5.0
Set 7	10.21 ± 5.2
Set 8	9.35 ± 5.1
No. power cleans in W	108.85 ± 24.4

G = Gymnastics WOD; M = Metabolic conditioning WOD; W = Weightlifting WOD; M ± SD = mean (± standard deviation).

### Capillary blood lactate

Table 3 provides descriptive data for blood lactate levels recorded in the three CrossFit WOD (S2 File). Repeated measures ANOVA detected significant differences in [lactate] pre- and post exercise (*F* = 1342.88, *P* = 0.000, *ES* = 0.931). For the group factor, significant differences were also observed (*F* = 3.995, *P* = 0.021, *ES* = 0.075), though no differences emerged for the interaction Group x Time (*F* = 2.620, *P* = 0.078, *ES* = 0.050). Through pairwise comparisons (Bonferroni post-hoc test) we detected differences in this variable between the G and M workouts (*P* = 0.017).

**Table 3:** Capillary blood lactate levels recorded in the 3 different CrossFit modalities in the 34 participants (M ± SD).

[Lactate] mmol·L^-1^	Preexercise	Postexercise
G	1.56 ± 0.61^†^	11.79 ± 2.33 *
M	1.30 ± 0.37	10.15 ± 3.04
W	1.22 ± 0.31	11.24 ± 2.62

G = Gymnastics WOD; M = Metabolic conditioning WOD; W = Weightlifting WOD.

* Significant difference between the G and M workouts (*P*<0.05).

^†^ Significant difference between the G and W workouts (*P*<0.05).

### Countermovement jump height and average and peak power losses

#### Gymnastics WOD

A significant reduction was produced across sets in CMJ height (*F* = 7.996; *P* = 0.003; *ES* = 0.195, SP = 0.886). A post-hoc comparison using the Bonferroni index of the H values recorded in each set identified significant differences between pre- and post exercise values (*P*<0.01) and no differences between the time points preexercise and minute–10 (*P* = 0.061). In contrast, significant differences did emerge between these two time points (preexercise vs minute–10) (*P*<0.05) for the variables PPR (*F* = 5.798; *P* = 0.005; *ES* = 0.149, SP = 0.855) and PPT (*F* = 5.081; *P* = 0.009; *ES* = 0.133, SP = 0.803), along with significant differences in these two variables also between minute–10 and postexercise (*P* = 0.014; *P* = 0.041 respectively) (S3 File) (Table 4).

**Table 4:** Variables reflecting muscular fatigue recorded at the 3 time points of the gymnastics WOD in the 34 participants.

Variables	Preexercise	Minute–10	Postexercise	% loss Pre-Post
**H (cm)**	38.06 ± 4.31 ^#^	36.23 ± 5.90	35.60 ± 4.65	-6.46
**PPR (watts·kg**^**-1**^**)**	56.48 ± 4.96 ^†^	54.69 ± 5.97 ^‡^	56.51 ± 5.98	0.053
**PPT (watts)**	4326.07 ± 498.31 ^†^	4188.23 ± 548.81 ^‡^	4325.036 ± 614.09	-0.016
**APR (watts·kg**^**-1**^**)**	32.37 ± 3.07 *	30.51 ± 4.13	31.01 ± 4.18	-4.2
**APT (watts)**	2479.01 ± 291.35 *	2336.57 ± 367.48	2381.82 ± 399.71	-3.92
**Vmax (m·s**^**-1**^**)**	2.886 ± 0.14 *	2.811 ± 0.17	2.807 ± 0.15	-2.74
**Fmax (N)**	2021.67 ± 237.48	1973.4 ± 224.23	1934.4 ± 361.17	-4.32
**Total duration (s)**	0.7219 ± 0.1034 ^†^	0.4786 ± 0.1201	0.7180 ± 0.1266	-0.54
**Duration ECC (s)**	0.4892 ± 0.0799 ^†^	0.2439 ± 0.0891 ^‡^	0.5104 ± 0.1186	+4.33
**Duration ISO (s)**	0.0098 ± 0.0044	0.0109 ± 0.0054	0.0109 ± 0.0039	+11.22
**Duration CON (s)**	0.2229 ± 0.0425 ^#^	0.2239 ± 0.0597 ^‡^	0.1966 ± 0.0568	-11.8
**RVD**_**peak**_ **(m·s**^**2–1**^**)**	4.1681 ± 0.5508	4.1337 ± 0.8453	4.0632 ± 0.628	-2.5
**RFD**_**peak**_ **(N·s**^**-1**^**)**	4235.24 ± 976.99	4150.36 ± 1113.64	3951.34 ± 1266.24	-6.7

H = jump height; PPR = peak power relative; PPT = peak power total; APR = average power relative; APT = average power total; Vmax = maximum takeoff velocity; Fmax = maximum force; ECC = eccentric phase; ISO = isometric phase; CON = concentric phase; RVD_peak_ = peak rate of velocity development; RFD_peak_ = peak rate of force development; % = percentage.

*significant difference preexercise *vs* minute–10 and postexercise (*P*<0.05).

^†^significant difference preexercise *vs* minute–10 (*P*<0.05).

^‡^significant difference minute–10 *vs* postexercise (*P*<0.05).

^#^significant difference preexercise *vs* postexercise (*P*<0.05).

For the variables APR, APT and Vmax, significant differences were also observed between different time points (*F* = 10.296, *P* = 0.000, *ES* = 0.238, SP = 0.984; *F* = 9.567, *P* = 0.000, *ES* = 0.225, SP = 0.976; *F* = 20.808, *P* = 0.000, *ES* = 0.387, SP = 0.999, respectively). Through Bonferroni pairwise comparisons, reductions in these variables were observed for preexercise values versus those recorded at the remaining time points (APR minute–10 *P* = 0.000, postexercise *P* = 0.010; APT minute–10 *P* = 0.000, postexercise *P* = 0.021; Vmax: minute–10 *P* = 0.000, postexercise *P* = 0.000, respectively) (S3 File) (Table 4).

Significant differences were detected in total CMJ duration across the three measurements made (*F* = 86.875, *P* = 0.000, *ES* = 0.725, SP = 1.000). However, no differences emerged between pre- and post exercise values (*P* = 1.000), though preexercise and minute–10 jump durations did vary significantly (*P* = 0.000) (S4 File) (Table 4). When we considered the durations of each jump stage, ECC, ISO and CON, it was observed that the difference in total jump duration between preexercise and minute–10 was many attributable to ECC, given significant differences between measurements (*P* = 0.000) (*F* = 113.92, *P* = 0.000, *ES* = 0.775, SP = 1.000) involving reduced ECC duration from preexercise to minute–10, followed by its recovery postexercise with no difference for pre- versus post exercise durations (*P* = 0.699). ISO stage duration showed an increase exceeding 11% from pre- to post exercise, though the difference between these two time points was not significant (*P* = 0.816). The same was observed for the CON phase between preexercise and minute–10 (*P* = 1.000). However, CON duration significantly fell between minute–10 and postexercise (*P* = 0.001) by almost 12%, while ISO phase durations at these two time points were similar (*P* = 1.000).

#### Metabolic conditioning WOD

Significant differences were produced in all variables indicating jump ability during this type of exercise (S5 File) (Table 5). A significant reduction was observed between the first time point (preexercise) and the time points sets 2, 4, 6 and 8 for H (*F* = 14.996, *P* = 0.000, *ES* = 0.312, SP = 1.000), PPR (*F* = 32.697, *P* = 0.000, *ES* = 0.498, PE = 1.000), PPT (*F* = 34.913, *P* = 0.000, *ES* = 0.514, SP = 1.000), APR (*F* = 15.085, *P* = 0.000, *ES* = 0.314, SP = 1.000), APT (*F* = 17.454, *P* = 0.000, *ES* = 0.346, SP = 1.000), and Vmax (*F* = 15.163, *P* = 0.000, *ES* = 0.315, SP = 1.000).

**Table 5:** Variables reflecting muscular fatigue recorded during the 6 sets of the metabolic conditioning WOD in the 34 study participants.

Variable	Preexercise	Set 2	Set 4	Set 6	Set 8	Postexercise	% loss Pre-Post
**H (cm)**	37.06 ± 4.28 *	34.11 ± 4.37	33.84 ± 4.63	33.51 ± 4.63 ^†^	34.24 ± 4.54	35.74 ± 5.34	-3.56
**PPR (watts·kg**^**-1**^**)**	55.58 ± 6.11 *	51.64 ± 6.06	51.84 ± 6.34	51.50 ± 5.75	52.87 ± 5.97	55.92 ± 6.97 ^‡^	0.61
**PPT (watts)**	4277.34 ± 553.10 *	3971.17 ± 546.13	3984.71 ± 552.00	3960.13 ± 521.78	3980.20 ± 528.12	4301.38 ± 633.19 ^‡^	0.56
**APR (watts·kg**^**-1**^**)**	31.83 ± 3.53 *	29.99 ± 3.42	29.59 ± 3.41	29.33 ± 3.36	29.58 ± 3.69	31.18 ± 3.94 ^#^	-2.04
**APT (watts)**	2445.22 ± 312.26 *	2303.88 ± 300.76	2271.73 ± 283.65	2255.04 ± 304.67	2226.90 ± 319.36	2400.56 ± 375.02 ^‡^	-1.82
**Vmax (m·s**^**-1**^**)**	2.843 ± 0.14 *	2.743 ± 0.15	2.754 ± 0.16	2.725 ± 0.15	2.761 ± 0.15	2.81 ± 0.17 ^‡^	-1.16
**Fmax (N)**	1998.19 ± 229.35	2027.34 ± 237.87	1972.74 ± 204.93	1968.07 ± 268.98	1428.7 ± 842.73 ^£^	1926.055 ± 445	-3.6
**Total duration (s)**	0.6851 ± 0.1478	0.6851 ± 0.1432	0.7011 ± 0.1565	0.7131 ± 0.1713	0.5417 ± 0.3398	0.6809 ± 0.1997	-0.61
**Duration ECC (s)**	0.4823 ± 0.0742	0.4555 ± 0.0725	0.4796 ± 0.0918	0.4883 ± 0.0978	0.3711 ± 0.2278	0.4997 ± 0.0961	+3.6
**Duration ISO (s)**	0.0087 ± 0.0030	0.0083 ± 0.0057	0.0084 ± 0.0043	0.0105 ± 0.0063	0.0140 ± 0.0176	0.0092 ± 0.0022	+5.75
**Duration CON (s)**	0.2126 ± 0.0503 ^$^	0.2409 ± 0.0442	0.2384 ± 0.0461	0.2370 ± 0.0524	0.2270 ± 0.0654	0.2128 ± 0.0571 ^¥^	+0.09
**RVD**_**peak**_ **(m·s**^**2–1**^**)**	4.2617 ± 0.5116 ^Σ^	4.1133 ± 0.4766	4.0582 ± 0.4672	3.9666 ± 0.664	3.847 ± 0.5678	4.079 ± 0.4631	-4.29
**RFD**_**peak**_ **(N·s**^**-1**^**)**	4332.34 ± 885.89	4588.22 ± 857.7 ^ß^	4288.17 ± 810.1	4297.33 ± 1482.8	3839.25 ± 869.9	3921.36 ± 897.1	-9.5

H = jump height; PPR = peak power relative; PPT = peak power total; APR = average power relative; APT = average power total; Vmax = maximum takeoff velocity; Fmax = maximum force; ECC = eccentric phase; ISO = isometric phase; CON = concentric phase; RVD_peak_ = peak rate of velocity development; RFD_peak_ = peak rate of force development; % = percentage.

* significant difference preexercise *vs* sets 2, 4, 6 and 8 (*P*<0.05).

^†^ significant difference set 6 *vs* postexercise (*P*<0.05).

^‡^ significant difference postexercise *vs* sets 2, 4, 6 and 8 (*P*<0.05).

^#^ significant difference postexercise *vs* sets 4, 6 and 8 (*P*<0.05).

^$^ significant difference preexercise *vs* sets 2 and 4 (*P*<0.05).

^¥^ significant difference postexercise *vs* sets 2 and 4 (*P*<0.05).

^Σ^ significant difference preexercise *vs* sets 6 and 8 (*P*<0.05).

^ß^ significant difference set 2 *vs* set 8 and postexercise (*P*<0.05).

^£^ significant difference set 8 *vs* preexercise and sets 2, 4 and 6 (*P*<0.05).

Pairwise Bonferroni comparisons indicated significant increases produced at the last time point 3 min after finishing the exercise session in PPR, PPT, APT, and Vmax compared to values recorded in sets 2, 4, 6 and 8, while for APR this same difference was detected with respect to sets 4, 6 and 8. For Fmax, significant differences (*F* = 10.870, *P* = 0.001, *ES* = 0.254, SP = 0.949) were observed between set 8 versus preexercise and sets 2, 4 and 6 (*P* = 0.011, *P* = 0.005, *P* = 0.015, *P* = 0.018, respectively).

Total jump durations failed to vary significantly among the different CMJ time points (*F* = 2.296, *P* = 0.071, *ES* = 0.284, SP = 0.653) (S6 File) (Table 5).

By exercise stage, neither were differences observed in ECC action duration, though jump height was reduced by 23% from the time points preexercise to set 8. Similarly, no significant differences emerged in ISO phase duration across time points (*F* = 1.829, *P* = 0.182, *ES* = 0.074, SP = 0.314), though ISO was longer in sets 6 (by 20.7%) and 8 (by 61%) compared to its preexercise value. The duration of CON did vary significantly during the jumps (*F* = 3.981, *P* = 0.012, *ES* = 0.148, SP = 0.811), being significantly longer in sets 2 and 4 compared to its preexercise duration (*P* = 0.014, *P* = 0.023, respectively) and significantly shorter in these sets compared to its postexercise duration (*P* = 0.010, *P* = 0.015, respectively) (S6 File) (Table 5).

Significant differences were also recorded for the RVD_peak_ data (*F* = 5.218, *P* = 0.001 *ES* = 0.185, SP = 0.968). Through pairwise Bonferroni comparisons, significant RVD_peak_ reductions of 6.9% to 9.7% were identified from the time points preexercise to sets 6 (*P* = 0.022) and 8 (*P* = 0.003), respectively. These acceleration capacity losses could be consistent with the lengthened ISO phase duration between preexercise and sets 6 (20.6%) and 8 (60.4%) (S6 File) (Table 5).

For the variable RFD_peak_, we also detected significant differences (*F* = 4.037, *P* = 0.002, *ES* = 0.149, SP = 0.834), including a lower value for set 2 versus 8 (*P* = 0.007) and postexercise (*P* = 0.002) and explosive force losses of 16.3% and 14.5% respectively. This diminished explosive force in set 8 and at the end of exercise could, as for RVD_peak_, have been influenced by the increased duration of the ISO component (S6 File) (Table 5).

#### Weightlifting WOD

During this exercise session, significant reductions were produced from preexercise to the other time points (minute–2.5 and postexercise) in many of the variables used to measure jump ability: H (*F* = 27.413, *P* = 0.000, *ES* = 0.454, SP = 1.000), PPR (*F* = 24.558, *P* = 0.000, *ES* = 0.427, SP = 1.000), PPT (*F* = 20.305, *P* = 0.000, *ES* = 0.381, SP = 1.000), APR (*F* = 38.995, *P* = 0.000, *ES* = 0.542, SP = 1.000), APT (*F* = 34.211, *P* = 0.000, *ES* = 0.509, SP = 1.000), Vmax (*F* = 32.374, *P* = 0.000, *ES* = 0.495, SP = 1.000) and Fmax (*F* = 26.271, *P* = 0.000, *ES* = 0.443, SP = 1.000) (S7 File) (Table 6).

**Table 6:** Variables reflecting muscular fatigue recorded at the three time points in the weightlifting session in the 34 study participants.

Variable	Preexercise	Minute–2.5	Postexercise	% loss Pre-Post
**H (cm)**	36.59 ± 4.04*	32.84 ± 5.26	33.90 ± 4.85	-7.35
**PPR (watts·kg**^**-1**^**)**	54.58 ± 5.36 *	51.20 ± 5.78 ^†^	53.03 ± 5.77	-2.84
**PPT (watts)**	4187.06 ± 535.76 *	3924.34 ± 527.88 ^†^	4071.46 ± 519.09	-2.76
**APR (watts·kg**^**-1**^**)**	30.95 ± 3.38 *	27.93 ± 4.06 ^†^	28.66 ± 3.88	-7.4
**APT (watts)**	2371.27 ± 309.48 *	2140.95 ± 348.23	2198.02 ± 319.38	-7.31
**Vmax (m·s**^**-1**^**)**	2.834 ± 0.12 *	2.706 ± 0.16 ^†^	2.745 ± 0.16	-3.14
**Fmax (N)**	2021.67 ± 237.48 *	1894.76 ± 272.76	1839.4 ± 293.18	-9.02
**Total duration (s)**	0.7203 ± 0.0908 ^‡^	0,7465 ± 0.0955	0,7431 ± 0.0966	+3.17
**Duration ECC (s)**	0.4892 ± 0,0799	0.4790 ± 0.0793	0.5076 ± 0.0893	+3.76
**Duration ISO (s)**	0.0098 ± 0.0044	0.0096 ± 0.005	0.0160 ± 0.0275	+63.26
**Duration CON (s)**	0.2229 ± 0.0425 ^‡^	0.2579 ± 0.0624 ^†^	0.2195 ± 0.0706	-1.53
**RVD**_**peak**_ **(m·s**^**2–1**^**)**	4.1681 ± 0.5508 *	3.804 ± 0.5817	3.8357 ± 0.5089	-7.97
**RFD**_**peak**_ **(N·s**^**-1**^**)**	4235.24 ± 976.993 ^#^	3906.72 ± 1458.124	3519.724 ± 916.586	-16.9

H = jump height; PPR = peak power relative; PPT = peak power total; APR = average power relative; APT = average power total; Vmax = maximum takeoff velocity; Fmax = maximum force; ECC = eccentric phase; ISO = isometric phase; CON = concentric phase; RVD_peak_ = peak rate of velocity development; RFD_peak_ = peak rate of force development; % = percentage.

*significant difference preexercise *vs* remaining time points (minute–2.5 and postexercise) (*P*<0.05).

^†^significant difference minute–2.5 *vs* postexercise (*P*<0.05).

^‡^significant difference preexercise *vs* minute–2.5 (P<0.05).

^#^significant difference pre- *vs* postexercise (P<0.05).

In effect, both Vmax and Fmax fell from their preexercise values by 4.5% and 5.8% at minute–2.5 and by 6.3% and 9% at minute–5, respectively. Reductions were even more marked for RVD_peak_ and RFD_peak_ (*F* = 13.471, *P* = 0.000, *ES* = 0.290, SP = 0.987; *F* = 9.391, *P* = 0.000, *ES* = 0.222, SP = 0.974 respectively), differences being significant for preexercise versus minute–2.5 (*P* = 0.003, 8.7%) and postexercise (*P* = 0.001, 8%) for RVD_peak_ and pre- versus post exercise (*P* = 0.000, -16.9%) for RFD_peak_ (S8 File) (Table 6).

It should be emphasized that despite a loss of jump capacity at the end of the exercise session with respect to its start, jumps were potentiated between minute–2.5 and postexercise. This means there were significant increases from the middle of exercise (2.5 min) to the end of exercise (3 min after exercise completion) in the jump ability indicators PPR, (*P* = 0.001), PPT, (*P* = 0.001), APR (*P* = 0.042), and Vmax (*P* = 0.012). However, their values were still significantly reduced compared to their preexercise values by -2.84%, -2.76%, -7.4% and -3.14% respectively (S7 File) (Table 6).

## Discussion

### Jump height, peak and average power, maximum takeoff velocity

Our study was designed to examine metabolic responses (blood lactate concentrations) and the metabolic variables inducing muscular fatigue (through jump ability measured in the CMJ test) in response to the three modalities of CrossFit workouts. One of the findings of this study was the high work intensity reflected by blood lactate levels in the three modalities of CrossFit, gymnastics, metabolic conditioning and weightlifting. At the end of each WOD, blood lactate levels were above 10 mmol·L^-1^, due to the glycolytic nature of the sessions. However, the main finding was the significantly reduced values of H, APR, APT and Vmax observed 3 minutes after the end of exercise indicating increased muscular fatigue for the G and W modalities in a CMJ test. In contrast, a significant loss of jump ability in the middle of the M workout was indicated by reductions between the time points preexercise and sessions 2, 4, 6 and 8 in H, PPR, PPT, APR, APT and Vmax. This suggests that fatigue worsened during the course of the session M though 3 minutes of rest after the session was sufficient for the return to normal values, unlike the situation following the G and W modalities.

This main finding of our study could be explained by the primary metabolism required for a CMJ from high-enery phosphagens [27], and its predominance in intermittent high-energy activities [28] such as the double skipping rope jump sets performed here in the M WOD. Hence, a 20 second-set of CrossFit double unders may partly deplete phosphocreatine stores. Moreover, short rest intervals between sets (10 s) might mean that this depletion progressed. Thus, the significant progressive loss of jump ability from the start to the completion of the session (set 8), was possibly the outcome of depleted phosphocreatine stores as suggested by other authors [27]. Given that phosphocreatine resynthesis shows a rapid stage in which 70% of these reserves are replenished in the first 30 seconds of exercise, and a second stage that lasts 3–5 minutes [29], the recovered jump ability observed here 3 minutes after the end of exercise is likely explained by recovered phosphocreatine levels.

This means that the muscular fatigue produced during the M WOD could be due to the depletion of high-energy phosphate deposits during the skipping rope sets, with starting jump ability recovered 3 minutes after exercise. This suggests that this rest period is appropriate between sets of interval exercise sessions such as M sessions in which exercises such as double unders are performed. In contrast, as training load is given by exercise intensity, volume and rest period, in our G and W workouts subjects performed the maximum number of “Cindy” rounds possible of power cleans, respectively, undertaking each repetition at maximum velocity. Further, the high exercise volume (20 and 5 minutes of exercise, respectively) and lack of recovery periods might mean that the demands of these sessions were fairly high. Thus, despite the light loads used in the sessions (bodyweight in G and 40% 1RM in W), these stimuli may have been sufficient to induce the muscular fatigue observed. The results obtained in the G and W sessions are consistent with the findings of Tibana et al. (2016) [16], who assessed mean power generated while executing 5 back squat repetitions using a load equivalent to 50% of the 1RM. In this study, a marked reduction was observed in muscular power production capacity after completing one CrossFit session and the authors considered this power loss to indicate muscular fatigue.

Evidence exists that fatigue levels may modify movement biomechanics [9] and technically demanding exercises, such as the squat, snatch, clean and jerk, generate tensions and overload in the shoulder joint and lumbar region [8]. This along with high exercise intensities and volumes means that this level of mechanical fatigue in G and M sessions determines a need for adequate rest periods. Moreover, before starting this type of CrossFit WOD individuals should undertake an anatomical adaptation and technical phase to avoid the risk of injury. In a case report, a healthy fit young adult was described to develop rhabdomyolysis after 5 days of high-intensity CrossFit training, requiring his hospitalization [7].

Mechanical fatigue arises from effects on contractile strength capacity which leads to an inability to generate force [15]. Among the factors that condition the appearance of muscular fatigue are the muscle group involved, exercise duration and intensity and the type of contractile action [30]. The high-intensity of G and W, involving stretch-shortening cycles and the lack of rest periods, may have provoked structural damage at muscle-tendon insertions reducing muscle-tendon stiffness [31, 32] and impairing CMJ performance [33].

### Capillary blood lactate levels

In addition, given their greater contraction velocity [34], type II motor units may have been preferentially recruited in the G and W WOD. These units are more susceptible to fatigue than motor I units and depend on a glycolytic metabolism [35]. The recruitment of these motor units was confirmed by the high blood lactate concentrations recorded in sessions G (11.79 ± 2.3 mmol·L^-1^) and W (11.06 ± 2.7 mmol·L^-1^). These results are in line with those reported by other investigators examining blood lactate responses to a CrossFit WOD [16].

Given that CMJ performance is greatly influenced by the motor units recruited [36], the fatigue of type II units in sessions G and W, may explain the progressive loss in jump capacity produced. In contrast, though session M was performed at high intensity (double skipping rope jumps) also requiring the recruitment of type II motor units, exercise volume was smaller (4 min total) and included rest periods (8 intervals of 10 seconds). This determined the recovery of CMJ ability at 3 minutes postexercise, likely due to the replenishment of phosphocreatine stores despite a final [lactate] of 10.2 mmol·L^-1^, which did not trigger muscular fatigue.

### Jump duration

To quantifying muscular fatigue through mechanical variables, some studies have focused on total jump duration [25, 26]. These investigations distinguish two jump stages: a negative movement phase (corresponding to ECC) and a positive movement phase (corresponding to CON). The start of ECC has been linked to the time point at which bodyweight falls by 2% [25] or 5% [26]. This stage finishes when angular displacement of the knee peaks [26] or when the net velocity of the center of mass is zero [25]. In both these studies, CON was found to start when ECC finished and continued until jump takeoff [25, 26]. Here, besides these two phases, we also measured ISO through vertical reaction forces using a force platform.

We observed here that ECC phase duration was slightly higher (though not significantly) in the CMJ performed 3 minutes after the end of each session than its preexercise value (G 4.3%, M 3.6%, W 3.8%). However, in intermediate sets, reductions were produced in ECC duration of -50.1% in session G, -23% (non-significant) in set 8 of session M and -2.1% in session W. This could indicate a slight shortening of ECC in the CMJ due to a diminished flexion angle of the knee when jumping in conditions of mechanical fatigue [26]. This reduced angle would give rise to a lesser moment of force around the knee joint, as the greater the levering arm in the knee (given by its degree of flexion) the greater will be the moment of force the knee needs to withstand [37]. Further, the non-use of elastic energy by the ECC action means that passive structures will need to cushion these loads, thus possibly increasing the risk of injury [13] [17]. While in G and M, leg work was essential for squats and double unders the neuromuscular fatigue detected in session W perhaps determined the poor execution of the power clean. This exercise uses the upper more than the lower trunk to lift the weighted barbell and this may have determined an adequate ECC phase in the postexercise CMJ.

The mechanism whereby the neuromuscular system signals the motor system to reduce the knee's flexion angle could be a reduction in muscle stiffness. This stiffness refers to a biological tissue's capacity to resist deformation and is reduced as the consequence of fatigue or in response to successive workloads without adequate rest periods [38]. Conversely, increased stiffness potentiates the stretch-shortening cycle [39], reducing the ISO phase and promoting the improved use of elastic energy. It is likely that the high intensity and high volume of exercise and lack of recovery periods in the G and W sessions led to diminished muscle and tendon stiffness around the knee joint [32]. Such compromised elastic properties of the muscle-tendon junction was confirmed by the substantially lengthened ISO phase produced in G (11.2%) and W (63.3%) at the end of exercise, albeit not significant. In the M session, non-significant ISO phase increases were also noted in sets 6 and 8 compared to its preexercise duration (20.7% and 61%, respectively). However, in M, although exercise intensity was high, exercise volume was not excessive and there were rest periods. Even so, impacts on the ground of CrossFit double unders might generate more stress in bones, tendons and joints, producing osteomuscular fatigue due to diminished transmission of information by peripheral mechanoreceptors towards the brain [40]. In contrast, reduced ISO phase duration from pre- to post exercise (5.8%) was observed in session M, and this was unaccompanied by differences in jump ability variables (H, APR, APT, PPR, PPT, Vmax, Fmax) indicating fatigue recovery 3 minutes after the session end.

### RVD_peak_

Such diminished neuromuscular performance due possibly to an impacted stretch-shortening cycle would be reflected in the results obtained for other variables such as Vmax, RVD_peak_, Fmax, and RFD_peak_. If we examine RVD_peak_, which represents the acceleration produced from 0 to Vmax reflecting an individual's capacity to generate high power levels until Vmax is reached [41, 42], its significant reduction was produced in workout W from pre- to post exercise (-8%) and in workout M between preexercise and sets 6 (-6.9%) and 8 (-9.7%). In G, this reduction was not significant (-2.5%). This finding is consistent with the significant decrease in power variables (APR, APT, PPR and PPT) produced in the three CrossFit WOD, except for PPT and PPR in session G, which is why a significant pre- to postexercise decrease in RVD_peak_ was not produced. That is, while on the one hand power production fell and high Vmax levels were not reached (being significantly lower post exercise than pre- in all three CrossFit WOD), on the other hand, ISO phase duration increased until Vmax, causing a reduction in RVD_peak_. Similarly, other studies have detected that muscle shortening velocity conditions takeoff velocity in a CMJ. This is crucial for jump performance [14, 43] and could be the outcome of the decreased power and lengthened ISO phase.

Since RVD_peak_ is a determining factor for sport performance, especially for activities in which muscle mass and/or an object need to be quickly displaced [44], the mechanical fatigue produced in the G and W workouts should be taken into account, since this loss of performance may mean that the athlete's targets are not attained.

## Limitations

In this study, we quantified levels of fatigue by measuring several mechanical variables in a CMJ test and compared test performance before, during and after the different WOD. However, while intrasession CMJ performance provides useful information on the behavior of the variables measured, this test could interfere with the normal execution of each of the WOD. This possible impact was minimized by making sure work:rest ratios (20:10) in the M workouts were unaffected by the CMJ test. This was achieved by rapidly placing the subject on the force platform and executing only one jump during the G and W sessions. This meant that intrasession jump tests involved only one jump per set in M (sets 2, 4, 6, 8) and one jump at minutes 10 and 2.5 only in the G and W WOD respectively.

## Conclusions

In conclusion, through the measurement of mechanical variables in a CMJ, our study assesses muscular fatigue produced in response to the different CrossFit WOD. Muscular fatigue was not observed at the end of the metabolic conditioning WOD and diminished jump capacity in this type of WOD only occurred after sets 2, 4, 6 and 8. These data indicate that while for an interval session of double unders, a 3 minute rest period would be appropriate, for the other session modalities, a longer rest period would be recommended. Further, given that in G and W the cause of mechanical fatigue might be the high intensity and volume of exercise along with the lack of rest intervals, individuals practicing these CrossFit modalities should first carry out an anatomical adaptation routine according to periodization strength training models [45]. For this prior strength training phase, we would recommend progressive adaptation of muscles, ligaments and especially muscle insertions in bone in an effort to improve tolerance to the high stresses of CrossFit sessions, thus reducing risks of injury. A further finding of our study was that the reduced muscle-tendon stiffness related to jump ability loss in the different WOD may have been the outcome of a lengthened ISO phase in the CMJ. This finding also requires confirmation in future work.

## Supporting information

S1 File Results descriptive data Crossfit. Statistical analysis performed with descriptive data.
(PDF)

S2 File Results 2_Factor_Test_ANOVA_LACTATE_Pre_Post_Crossfit. Statistical analysis performed with blood lactate concentrations.
(PDF)

S3 File Results_GYMNASTIC_Jump Height_ Peak and Average Power_ Vmax_ Repeated_ Measures_ ANOVA_Crossfit. Statistical analysis performed with mechanical variables in gymnastic WOD.
(PDF)

S4 File Results_GYMNASTIC_Jump_Duration_Repeated_Measures_ANOVA_Crossfit. Statistical analysis performed with mechanical variables in gymnastic WOD.
(PDF)

S5 File Results_METABOLIC_ Jump Height_ Peak and Average Power_ Vmax_ Repeated_ Measures_ ANOVA_Crossfit. Statistical analysis performed with mechanical variables in Metabolic WOD.
(PDF)

S6 File Results_METABOLIC_Jump_Duration_Repeated_Measures_ANOVA_Crossfit. Statistical analysis performed with mechanical variables in Metabolic WOD
(PDF)

S7 File Results_WEIGHT_ Jump Height_ Peak and Average Power_ Vmax_ Repeated_ Measures_ ANOVA_Crossfit. Statistical analysis performed with mechanical variables in Weight WOD.
(PDF)

S8 File Results_WEIGHT_Jump_Duration_Repeated_Measures_ANOVA_Crossfit. Statistical analysis performed with mechanical variables in Weight WOD.
(PDF)
