# Supplementary material for: Muscular fatigue in response to different modalities of CrossFit sessions
Source: PLoS One. 2017 Jul 28;12(7):e0181855. doi: 10.1371/journal.pone.0181855 (PMC5533437; doi:10.1371/journal.pone.0181855)
Supplement: S1 File — Statistical analysis performed with descriptive data. (PDF) [file pone.0181855.s001.pdf]

Estadísticos descriptivos

	N	Mínimo	Máximo	Media	Desv. típ.
Edad	34	19,00	33,00	22,0294	3,14773
Peso	34	62,90	90,60	76,8997	7,04920
Talla	34	1,61	1,92	1,7865	,06427
G_Sindys	34	16,00	26,00	19,5882	2,59507
M_nº_Dcomba_S1	34	2,00	30,00	13,8529	7,73474
M_nº_Dcomba_S2	34	1,00	35,00	12,7647	7,21135
M_nº_Dcomba_S3	34	4,00	28,00	11,7059	6,45980
M_nº_Dcomba_S4	34	3,00	21,00	9,6176	4,94218
M_nº_Dcomba_S5	34	4,00	25,00	10,6471	5,70424
M_nº_Dcomba_S6	34	3,00	21,00	9,8824	5,00766
M_nº_Dcomba_S7	34	3,00	23,00	10,2059	5,21524
M_nº_Dcomba_S8	34	2,00	21,00	9,3529	5,06253
W_nº_Cargadas_Completas	34	76,00	186,00	108,8529	24,35984
N válido (según lista)	34				
