# Supplementary material for: Muscular fatigue in response to different modalities of CrossFit sessions
Source: PLoS One. 2017 Jul 28;12(7):e0181855. doi: 10.1371/journal.pone.0181855 (PMC5533437; doi:10.1371/journal.pone.0181855)
Supplement: S2 File — Statistical analysis performed with blood lactate concentrations. (PDF) [file pone.0181855.s002.pdf]

LACTATE

Factores intra-sujetos

Medida:MEASURE_1

TIEMPO	Variable dependiente
1	Lactato_reposo
2	Lactato_final

Factores inter-sujetos

		Etiqueta del valor	N
Grupos	1	Gimnástica	34
	2	Metabólico	34
	3	Weight	34

Estadísticos descriptivos

	Grupos	Media	Desviación típica	N
Lactato_reposo	Gimnástica	1,5647	,61540	34
	Metabólico	1,3059	,37086	34
	Weight	1,2265	,31843	34
	Total	1,3657	,47209	102
Lactato_final	Gimnástica	11,7971	2,33049	34
	Metabólico	10,1588	3,04383	34
	Weight	11,2441	2,62819	34
	Total	11,0667	2,74339	102

Contrastes multivariados[c]

Efecto		Valor	F	Gl de la hipótesis	Gl del error	Sig.	Eta al cuadrado parcial	Parámetro de no centralidad Parámetro	Potencia observada[b]
TIEMPO	Traza de Pillai	,931	1342,879[a]	1,000	99,000	,000	,931	1342,879	1,000
	Lambda de Wilks	,069	1342,879[a]	1,000	99,000	,000	,931	1342,879	1,000
	Traza de Hotelling	13,564	1342,879[a]	1,000	99,000	,000	,931	1342,879	1,000
	Raíz mayor de Roy	13,564	1342,879[a]	1,000	99,000	,000	,931	1342,879	1,000
TIEMPO * Grupos	Traza de Pillai	,050	2,620[a]	2,000	99,000	,078	,050	5,241	,511
	Lambda de Wilks	,950	2,620[a]	2,000	99,000	,078	,050	5,241	,511
	Traza de Hotelling	,053	2,620[a]	2,000	99,000	,078	,050	5,241	,511
	Raíz mayor de Roy	,053	2,620[a]	2,000	99,000	,078	,050	5,241	,511

a. Estadístico exacto

b. Calculado con alfa = ,05

c. Diseño: Intersección + Grupos

Diseño intra-sujetos: TIEMPO

Prueba de esfericidad de Mauchly[b]

Medida:MEASURE_1

Efecto intra-sujetos	W de Mauchly	Chi-cuadrado aprox.	gl	Sig.	Epsilon[a] Greenhouse-Geisser	Huynh-Feldt	Límite-inferior
TIEMPO	1,000	,000	0	.	1,000	1,000	1,000

Contrasta la hipótesis nula de que la matriz de covarianza error de las variables dependientes transformadas es proporcional a una matriz identidad.

a. Puede usarse para corregir los grados de libertad en las pruebas de significación promediadas. Las pruebas corregidas se muestran en la tabla Pruebas de los efectos inter-sujetos.

b. Diseño: Intersección + Grupos
Diseño intra-sujetos: TIEMPO

Pruebas de efectos intra-sujetos.

Medida:MEASURE_1

Origen		Suma de cuadrados tipo III	gl	Media cuadrática	F	Sig.	Eta al cuadrado parcial	Parámetro de no centralidad Parámetro	Potencia observada[a]
TIEMPO	Esfericidad asumida	4799,560	1	4799,560	1342,879	,000	,931	1342,879	1,000
	Greenhouse-Geisser	4799,560	1,000	4799,560	1342,879	,000	,931	1342,879	1,000
	Huynh-Feldt	4799,560	1,000	4799,560	1342,879	,000	,931	1342,879	1,000
	Límite-inferior	4799,560	1,000	4799,560	1342,879	,000	,931	1342,879	1,000
TIEMPO * Grupos	Esfericidad asumida	18,731	2	9,365	2,620	,078	,050	5,241	,511
	Greenhouse-Geisser	18,731	2,000	9,365	2,620	,078	,050	5,241	,511
	Huynh-Feldt	18,731	2,000	9,365	2,620	,078	,050	5,241	,511
	Límite-inferior	18,731	2,000	9,365	2,620	,078	,050	5,241	,511
Error(TIEMPO)	Esfericidad asumida	353,834	99	3,574					
	Greenhouse-Geisser	353,834	99,000	3,574					
	Huynh-Feldt	353,834	99,000	3,574					
	Límite-inferior	353,834	99,000	3,574					

a. Calculado con alfa = ,05

Pruebas de contrastes intra-sujetos

Medida:MEASURE_1

Origen	TIEMPO	Suma de cuadrados tipo III	gl	Media cuadrática	F	Sig.	Eta al cuadrado parcial	Parámetro de no centralidad Parámetro	Potencia observada[a]
TIEMPO	Lineal	4799,560	1	4799,560	1342,879	,000	,931	1342,879	1,000
TIEMPO * Grupos	Lineal	18,731	2	9,365	2,620	,078	,050	5,241	,511
Error(TIEMPO)	Lineal	353,834	99	3,574					

a. Calculado con alfa = ,05

Pruebas de los efectos inter-sujetos

Medida:MEASURE_1

Variable transformada:Promedio

Origen	Suma de cuadrados tipo III	gl	Media cuadrática	F	Sig.	Eta al cuadrado parcial	Parámetro de no centralidad Parámetro	Potencia observada[a]
Intersección	7882,733	1	7882,733	2056,559	,000	,954	2056,559	1,000
Grupos	30,627	2	15,314	3,995	,021	,075	7,990	,703
Error	379,464	99	3,833					

a. Calculado con alfa = ,05

Comparaciones por pares

Medida:MEASURE_1

(I)Grupos	(J)Grupos	Diferencia de medias (I-J)	Error típ.	Sig.[a]	Intervalo de confianza al 95 % para la diferencia[a]	
					Límite inferior	Límite superior
Gimnástica	Metabólico	,949*	,336	,006	,282	1,615
	Weight	,446	,336	,188	-,221	1,112
Metabólico	Gimnástica	-,949*	,336	,006	-1,615	-,282
	Weight	-,503	,336	,137	-1,169	,163
Weight	Gimnástica	-,446	,336	,188	-1,112	,221
	Metabólico	,503	,336	,137	-,163	1,169

Basadas en las medias marginales estimadas.

*. La diferencia de medias es significativa al nivel ,05.

a. Ajuste para comparaciones múltiples: Diferencia menos significativa (equivalente a la ausencia de ajuste).

3. Grupos * TIEMPO

Medida:MEASURE_1

Grupos	TIEMPO	Media	Error típ.	Intervalo de confianza 95%	
				Límite inferior	Límite superior
Gimnástica	1	1,565	,078	1,410	1,719
	2	11,797	,460	10,884	12,710
Metabólico	1	1,306	,078	1,151	1,460
	2	10,159	,460	9,246	11,072
Weight	1	1,226	,078	1,072	1,381
	2	11,244	,460	10,331	12,157

Pruebas post hoc

Comparaciones múltiples

MEASURE_1

Bonferroni

(I)Grupos	(J)Grupos	Diferencia de medias (I-J)	Error típ.	Sig.	Intervalo de confianza 95%	
					Límite inferior	Límite superior
Gimnástica	Metabólico	,9485*	,33576	,017	,1308	1,7662
	Weight	,4456	,33576	,563	-,3721	1,2633
Metabólico	Gimnástica	-,9485*	,33576	,017	-1,7662	-,1308
	Weight	-,5029	,33576	,412	-1,3206	,3147
Weight	Gimnástica	-,4456	,33576	,563	-1,2633	,3721
	Metabólico	,5029	,33576	,412	-,3147	1,3206

Basadas en las medias observadas.

El término de error es la media cuadrática(Error) = 1,916.

*. La diferencia de medias es significativa al nivel ,05.
