# Supplementary material for: Muscular fatigue in response to different modalities of CrossFit sessions
Source: PLoS One. 2017 Jul 28;12(7):e0181855. doi: 10.1371/journal.pone.0181855 (PMC5533437; doi:10.1371/journal.pone.0181855)
Supplement: S3 File — Statistical analysis performed with mechanical variables in gymnastic WOD. (PDF) [file pone.0181855.s003.pdf]

JUMP HEIGHT

Factores intra-sujetos

Medida:MEASURE_1

Tiempo	Variable dependiente
1	G_CMJ_Media_Pre_Alt_Vue
2	G_CMJ1_min10_AlturaVuelo
3	G_CMJ_Med_post_AltuVuelo

Estadísticos descriptivos

	Media	Desviación típica	N
G_CMJ_Media_Pre_Alt_Vue	38,0661	4,31499	34
G_CMJ1_min10_AlturaVuelo	36,2343	5,90805	34
G_CMJ_Med_post_AltuVuelo	35,6091	4,65009	34

Contrastes multivariados[c]

Efecto		Valor	F	Gl de la hipótesis	Gl del error	Sig.	Eta al cuadrado parcial	Parámetro de no centralidad Parámetro	Potencia observada[b]
Tiempo	Traza de Pillai	,526	17,754[a]	2,000	32,000	,000	,526	35,508	1,000
	Lambda de Wilks	,474	17,754[a]	2,000	32,000	,000	,526	35,508	1,000
	Traza de Hotelling	1,110	17,754[a]	2,000	32,000	,000	,526	35,508	1,000
	Raíz mayor de Roy	1,110	17,754[a]	2,000	32,000	,000	,526	35,508	1,000

a. Estadístico exacto

b. Calculado con alfa = ,05

c. Diseño: Intersección

Diseño intra-sujetos: Tiempo

Prueba de esfericidad de Mauchly[b]

Medida:MEASURE_1

Efecto intra-sujetos	W de Mauchly	Chi-cuadrado aprox.	gl	Sig.	Epsilon[a]		
					Greenhouse-Geisser	Huynh-Feldt	Límite-inferior
Tiempo	,635	14,534	2	,001	,733	,758	,500

Contrasta la hipótesis nula de que la matriz de covarianza error de las variables dependientes transformadas es proporcional a una matriz identidad.

a. Puede usarse para corregir los grados de libertad en las pruebas de significación promediadas. Las pruebas corregidas se muestran en la tabla Pruebas de los efectos inter-sujetos.

b. Diseño: Intersección

Diseño intra-sujetos: Tiempo

Pruebas de efectos intra-sujetos.

Medida:MEASURE_1

Origen		Suma de cuadrados tipo III	gl	Media cuadrática	F	Sig.	Eta al cuadrado parcial	Parámetro de no centralidad Parámetro	Potencia observada[a]
Tiempo	Esfericidad asumida	110,880	2	55,440	7,996	,001	,195	15,991	,948
	Greenhouse-Geisser	110,880	1,465	75,677	7,996	,003	,195	11,715	,886
	Huynh-Feldt	110,880	1,516	73,126	7,996	,002	,195	12,124	,894
	Límite-inferior	110,880	1,000	110,880	7,996	,008	,195	7,996	,784
Error(Tiempo)	Esfericidad asumida	457,627	66	6,934					
	Greenhouse-Geisser	457,627	48,351	9,465					
	Huynh-Feldt	457,627	50,037	9,146					
	Límite-inferior	457,627	33,000	13,867					

a. Calculado con alfa = ,05

Comparaciones por pares

Medida:MEASURE_1

(I)Tiempo	(J)Tiempo	Diferencia de medias (I-J)	Error típ.	Sig.[a]	Intervalo de confianza al 95 % para la diferencia[a]	
					Límite inferior	Límite superior
1	2	1,832	,752	,061	-,064	3,728
	3	2,457*	,406	,000	1,433	3,481
2	1	-1,832	,752	,061	-3,728	,064
	3	,625	,703	1,000	-1,147	2,397
3	1	-2,457*	,406	,000	-3,481	-1,433
	2	-,625	,703	1,000	-2,397	1,147

Basadas en las medias marginales estimadas.

PEAK POWER RELATIVE

Factores intra-sujetos

Medida:MEASURE_1

Tiempo	Variable dependiente
1	G_CMJ_Med_Pre_Pmax_rel
2	G_CMJ_min10_Pmax_rel
3	G_CMJ_Med_post_Pmax_rel

Estadísticos descriptivos

	Media	Desviación típica	N
G_CMJ_Med_Pre_Pmax_rel	56,4888	4,96262	34
G_CMJ_min10_Pmax_rel	54,6927	5,97486	34
G_CMJ_Med_post_Pmax_rel	56,5116	5,97713	34

Contrastes multivariados[c]

Efecto		Valor	F	Gl de la hipótesis	Gl del error	Sig.	Eta al cuadrado parcial	Parámetro de no centralidad Parámetro	Potencia observada[b]
Tiempo	Traza de Pillai	,251	5,362[a]	2,000	32,000	,010	,251	10,723	,805
	Lambda de Wilks	,749	5,362[a]	2,000	32,000	,010	,251	10,723	,805
	Traza de Hotelling	,335	5,362[a]	2,000	32,000	,010	,251	10,723	,805
	Raíz mayor de Roy	,335	5,362[a]	2,000	32,000	,010	,251	10,723	,805

a. Estadístico exacto

b. Calculado con alfa = ,05

c. Diseño: Intersección

Diseño intra-sujetos: Tiempo

Prueba de esfericidad de Mauchly[b]

Medida:MEASURE_1

Efecto intra-sujetos	W de Mauchly	Chi-cuadrado aprox.	gl	Sig.	Epsilon[a]		
					Greenhouse-Geisser	Huynh-Feldt	Límite-inferior
Tiempo	,989	,359	2	,836	,989	1,000	,500

Contrasta la hipótesis nula de que la matriz de covarianza error de las variables dependientes transformadas es proporcional a una matriz identidad.

a. Puede usarse para corregir los grados de libertad en las pruebas de significación promediadas. Las pruebas corregidas se muestran en la tabla Pruebas de los efectos inter-sujetos.

b. Diseño: Intersección

Diseño intra-sujetos: Tiempo

Pruebas de efectos intra-sujetos.

Medida:MEASURE_1

Origen		Suma de cuadrados tipo III	gl	Media cuadrática	F	Sig.	Eta al cuadrado parcial	Parámetro de no centralidad Parámetro	Potencia observada[a]
Tiempo	Esfericidad asumida	74,059	2	37,029	5,798	,005	,149	11,595	,855
	Greenhouse-Geisser	74,059	1,978	37,442	5,798	,005	,149	11,467	,852
	Huynh-Feldt	74,059	2,000	37,029	5,798	,005	,149	11,595	,855
	Límite-inferior	74,059	1,000	74,059	5,798	,022	,149	5,798	,647
Error(Tiempo)	Esfericidad asumida	421,539	66	6,387					
	Greenhouse-Geisser	421,539	65,272	6,458					
	Huynh-Feldt	421,539	66,000	6,387					
	Límite-inferior	421,539	33,000	12,774					

a. Calculado con alfa = ,05

Comparaciones por pares

Medida:MEASURE_1

(I)Tiempo	(J)Tiempo	Diferencia de medias (I-J)	Error típ.	Sig.[a]	Intervalo de confianza al 95 % para la diferencia[a]	
					Límite inferior	Límite superior
1	2	1,796*	,644	,026	,171	3,421
	3	-,023	,595	1,000	-1,523	1,477
2	1	-1,796*	,644	,026	-3,421	-,171
	3	-1,819*	,599	,014	-3,328	-,309
3	1	,023	,595	1,000	-1,477	1,523
	2	1,819*	,599	,014	,309	3,328

Basadas en las medias marginales estimadas.

*. La diferencia de medias es significativa al nivel ,05.

a. Ajuste para comparaciones múltiples: Bonferroni.

PEAK POWER TOTAL

Factores intra-sujetos

Medida:MEASURE_1

Tiempo	Variable dependiente
1	G_CMJ_Med_Pre_Pmax_total
2	G_CMJ_min10_Pmax_total
3	G_CMJ_Med_post_Pmax_total

Estadísticos descriptivos

	Media	Desviación típica	N
G_CMJ_Med_Pre_Pmax_total	4326,0710	498,31526	34
G_CMJ_min10_Pmax_total	4188,2385	548,81780	34
G_CMJ_Med_post_Pmax_total	4325,3634	614,09286	34

Contrastes multivariados[c]

Efecto		Valor	F	Gl de la hipótesis	Gl del error	Sig.	Eta al cuadrado parcial	Parámetro de no centralidad Parámetro	Potencia observada[b]
Tiempo	Traza de Pillai	,215	4,376[a]	2,000	32,000	,021	,215	8,752	,715
	Lambda de Wilks	,785	4,376[a]	2,000	32,000	,021	,215	8,752	,715
	Traza de Hotelling	,273	4,376[a]	2,000	32,000	,021	,215	8,752	,715
	Raíz mayor de Roy	,273	4,376[a]	2,000	32,000	,021	,215	8,752	,715

a. Estadístico exacto

b. Calculado con alfa = ,05

c. Diseño: Intersección

Diseño intra-sujetos: Tiempo

Prueba de esfericidad de Mauchly[b]

Medida:MEASURE_1

Efecto intra-sujetos	W de Mauchly	Chi-cuadrado aprox.	gl	Sig.	Epsilon[a]		
					Greenhouse-Geisser	Huynh-Feldt	Límite-inferior
Tiempo	,979	,687	2	,709	,979	1,000	,500

Contrasta la hipótesis nula de que la matriz de covarianza error de las variables dependientes transformadas es proporcional a una matriz identidad.

a. Puede usarse para corregir los grados de libertad en las pruebas de significación promediadas. Las pruebas corregidas se muestran en la tabla Pruebas de los efectos inter-sujetos.

b. Diseño: Intersección

Diseño intra-sujetos: Tiempo

Pruebas de efectos intra-sujetos.

Medida:MEASURE_1

Origen		Suma de cuadrados tipo III	gl	Media cuadrática	F	Sig.	Eta al cuadrado parcial	Parámetro de no centralidad Parámetro	Potencia observada[a]
Tiempo	Esfericidad asumida	428417,072	2	214208,536	5,081	,009	,133	10,163	,803
	Greenhouse-Geisser	428417,072	1,958	218760,191	5,081	,009	,133	9,951	,797
	Huynh-Feldt	428417,072	2,000	214208,536	5,081	,009	,133	10,163	,803
	Límite-inferior	428417,072	1,000	428417,072	5,081	,031	,133	5,081	,590
Error(Tiempo)	Esfericidad asumida	2782257,646	66	42155,419					
	Greenhouse-Geisser	2782257,646	64,627	43051,167					
	Huynh-Feldt	2782257,646	66,000	42155,419					
	Límite-inferior	2782257,646	33,000	84310,838					

a. Calculado con alfa = ,05

Comparaciones por pares

Medida:MEASURE_1

(I)Tiempo	(J)Tiempo	Diferencia de medias (I-J)	Error típ.	Sig.[a]	Intervalo de confianza al 95 % para la diferencia[a]	
					Límite inferior	Límite superior
1	2	137,832*	50,070	,029	11,545	264,120
	3	,708	46,411	1,000	-116,350	117,765
2	1	-137,832*	50,070	,029	-264,120	-11,545
	3	-137,125*	52,709	,041	-270,068	-4,182
3	1	-,708	46,411	1,000	-117,765	116,350
	2	137,125*	52,709	,041	4,182	270,068

Basadas en las medias marginales estimadas.

*. La diferencia de medias es significativa al nivel ,05.

a. Ajuste para comparaciones múltiples: Bonferroni.

AVERAGE POWER RELATIVE

Factores intra-sujetos

Medida:MEASURE_1

Tiempo	Variable dependiente
1	G_CMJ_Med_Pre_Pmed_rel
2	G_CMJ_min10_Pmed_rel
3	G_CMJ_Med_post_Pmed_rel

Estadísticos descriptivos

	Media	Desviación típica	N
G_CMJ_Med_Pre_Pmed_rel	32,3720	3,07092	34
G_CMJ_min10_Pmed_rel	30,5130	4,13823	34
G_CMJ_Med_post_Pmed_rel	31,0175	4,18105	34

Contrastes multivariados[c]

Efecto		Valor	F	Gl de la hipótesis	Gl del error	Sig.	Eta al cuadrado parcial	Parámetro de no centralidad Parámetro	Potencia observada[b]
Tiempo	Traza de Pillai	,373	9,530[a]	2,000	32,000	,001	,373	19,060	,969
	Lambda de Wilks	,627	9,530[a]	2,000	32,000	,001	,373	19,060	,969
	Traza de Hotelling	,596	9,530[a]	2,000	32,000	,001	,373	19,060	,969
	Raíz mayor de Roy	,596	9,530[a]	2,000	32,000	,001	,373	19,060	,969

a. Estadístico exacto

b. Calculado con alfa = ,05

c. Diseño: Intersección

Diseño intra-sujetos: Tiempo

Prueba de esfericidad de Mauchly[b]

Medida:MEASURE_1

Efecto intra-sujetos	W de Mauchly	Chi-cuadrado aprox.	gl	Sig.	Epsilon[a]		
					Greenhouse-Geisser	Huynh-Feldt	Límite-inferior
Tiempo	,997	,102	2	,950	,997	1,000	,500

Contrasta la hipótesis nula de que la matriz de covarianza error de las variables dependientes transformadas es proporcional a una matriz identidad.

a. Puede usarse para corregir los grados de libertad en las pruebas de significación promediadas. Las pruebas corregidas se muestran en la tabla Pruebas de los efectos inter-sujetos.

b. Diseño: Intersección

Diseño intra-sujetos: Tiempo

Pruebas de efectos intra-sujetos.

Medida:MEASURE_1

Origen		Suma de cuadrados tipo III	gl	Media cuadrática	F	Sig.	Eta al cuadrado parcial	Parámetro de no centralidad Parámetro	Potencia observada[a]
Tiempo	Esfericidad asumida	62,840	2	31,420	10,296	,000	,238	20,591	,984
	Greenhouse-Geisser	62,840	1,994	31,521	10,296	,000	,238	20,526	,984
	Huynh-Feldt	62,840	2,000	31,420	10,296	,000	,238	20,591	,984
	Límite-inferior	62,840	1,000	62,840	10,296	,003	,238	10,296	,876
Error(Tiempo)	Esfericidad asumida	201,420	66	3,052					
	Greenhouse-Geisser	201,420	65,790	3,062					
	Huynh-Feldt	201,420	66,000	3,052					
	Límite-inferior	201,420	33,000	6,104					

a. Calculado con alfa = ,05

Comparaciones por pares

Medida:MEASURE_1

(I)Tiempo	(J)Tiempo	Diferencia de medias (I-J)	Error típ.	Sig.[a]	Intervalo de confianza al 95 % para la diferencia[a]	
					Límite inferior	Límite superior
1	2	1,859*	,430	,000	,776	2,942
	3	1,354*	,430	,010	,271	2,438
2	1	-1,859*	,430	,000	-2,942	-,776
	3	-,504	,412	,687	-1,543	,534
3	1	-1,354*	,430	,010	-2,438	-,271
	2	,504	,412	,687	-,534	1,543

Basadas en las medias marginales estimadas.

*. La diferencia de medias es significativa al nivel ,05.

a. Ajuste para comparaciones múltiples: Bonferroni.

AVERAGE POWER TOTAL

Factores intra-sujetos

Medida:MEASURE_1

Tiempo	Variable dependiente
1	G_CMJ_Med_Pre_Pmed_total
2	G_CMJ_min10_Pmed_total
3	G_CMJ_Med_post_Pmed_total

Estadísticos descriptivos

	Media	Desviación típica	N
G_CMJ_Med_Pre_Pmed_total	2476,0079	291,35833	34
G_CMJ_min10_Pmed_total	2336,5798	367,48640	34
G_CMJ_Med_post_Pmed_total	2381,8290	399,71630	34

Contrastes multivariados[c]

Efecto		Valor	F	Gl de la hipótesis	Gl del error	Sig.	Eta al cuadrado parcial	Parámetro de no centralidad Parámetro	Potencia observada[b]
Tiempo	Traza de Pillai	,367	9,285[a]	2,000	32,000	,001	,367	18,570	,965
	Lambda de Wilks	,633	9,285[a]	2,000	32,000	,001	,367	18,570	,965
	Traza de Hotelling	,580	9,285[a]	2,000	32,000	,001	,367	18,570	,965
	Raíz mayor de Roy	,580	9,285[a]	2,000	32,000	,001	,367	18,570	,965

a. Estadístico exacto

b. Calculado con alfa = ,05

c. Diseño: Intersección

Diseño intra-sujetos: Tiempo

Prueba de esfericidad de Mauchly[b]

Medida:MEASURE_1

Efecto intra-sujetos	W de Mauchly	Chi-cuadrado aprox.	gl	Sig.	Epsilon[a]		
					Greenhouse-Geisser	Huynh-Feldt	Límite-inferior
Tiempo	1,000	,006	2	,997	1,000	1,000	,500

Contrasta la hipótesis nula de que la matriz de covarianza error de las variables dependientes transformadas es proporcional a una matriz identidad.

a. Puede usarse para corregir los grados de libertad en las pruebas de significación promediadas. Las pruebas corregidas se muestran en la tabla Pruebas de los efectos inter-sujetos.

b. Diseño: Intersección

Diseño intra-sujetos: Tiempo

Pruebas de efectos intra-sujetos.

Medida:MEASURE_1

Origen		Suma de cuadrados tipo III	gl	Media cuadrática	F	Sig.	Eta al cuadrado parcial	Parámetro de no centralidad Parámetro	Potencia observada[a]
Tiempo	Esfericidad asumida	344050,384	2	172025,192	9,567	,000	,225	19,134	,976
	Greenhouse-Geisser	344050,384	2,000	172058,118	9,567	,000	,225	19,131	,976
	Huynh-Feldt	344050,384	2,000	172025,192	9,567	,000	,225	19,134	,976
	Límite-inferior	344050,384	1,000	344050,384	9,567	,004	,225	9,567	,851
Error(Tiempo)	Esfericidad asumida	1186727,487	66	17980,719					
	Greenhouse-Geisser	1186727,487	65,987	17984,161					
	Huynh-Feldt	1186727,487	66,000	17980,719					
	Límite-inferior	1186727,487	33,000	35961,439					

a. Calculado con alfa = ,05

Comparaciones por pares

Medida:MEASURE_1

(I)Tiempo	(J)Tiempo	Diferencia de medias (I-J)	Error típ.	Sig.[a]	Intervalo de confianza al 95 % para la diferencia[a]	
					Límite inferior	Límite superior
1	2	139,428*	32,425	,000	57,645	221,211
	3	94,179*	32,746	,021	11,587	176,770
2	1	-139,428*	32,425	,000	-221,211	-57,645
	3	-45,249	32,394	,515	-126,955	36,456
3	1	-94,179*	32,746	,021	-176,770	-11,587
	2	45,249	32,394	,515	-36,456	126,955

Basadas en las medias marginales estimadas.

*. La diferencia de medias es significativa al nivel ,05.

a. Ajuste para comparaciones múltiples: Bonferroni.

MAXIMUM FORCE

Factores intra-sujetos

Medida:MEASURE_1

Tiempo	Variable dependiente
1	G_CMJ_Media_Pre_Fmax
2	G_CMJ1_min10_Fmax
3	G_CMJ_Media_Post_Fmax

Estadísticos descriptivos

	Media	Desviación típica	N
G_CMJ_Media_Pre_Fmax	2,5936	,29255	34
G_CMJ1_min10_Fmax	2,5386	,25740	34
G_CMJ_Media_Post_Fmax	2,5470	,30162	34

Contrastes multivariados[c]

Efecto		Valor	F	Gl de la hipótesis	Gl del error	Sig.	Eta al cuadrado parcial	Parámetro de no centralidad Parámetro	Potencia observada[b]
Tiempo	Traza de Pillai	,139	2,587[a]	2,000	32,000	,091	,139	5,175	,479
	Lambda de Wilks	,861	2,587[a]	2,000	32,000	,091	,139	5,175	,479
	Traza de Hotelling	,162	2,587[a]	2,000	32,000	,091	,139	5,175	,479
	Raíz mayor de Roy	,162	2,587[a]	2,000	32,000	,091	,139	5,175	,479

a. Estadístico exacto

b. Calculado con alfa = ,05

c. Diseño: Intersección

Diseño intra-sujetos: Tiempo

Prueba de esfericidad de Mauchly[b]

Medida:MEASURE_1

Efecto intra-sujetos	W de Mauchly	Chi-cuadrado aprox.	gl	Sig.	Epsilon[a]		
					Greenhouse-Geisser	Huynh-Feldt	Límite-inferior
Tiempo	,740	9,622	2	,008	,794	,827	,500

Contrasta la hipótesis nula de que la matriz de covarianza error de las variables dependientes transformadas es proporcional a una matriz identidad.

a. Puede usarse para corregir los grados de libertad en las pruebas de significación promediadas. Las pruebas corregidas se muestran en la tabla Pruebas de los efectos inter-sujetos.

b. Diseño: Intersección

Diseño intra-sujetos: Tiempo

Pruebas de efectos intra-sujetos.

Medida:MEASURE_1

Origen		Suma de cuadrados tipo III	gl	Media cuadrática	F	Sig.	Eta al cuadrado parcial	Parámetro de no centralidad Parámetro	Potencia observada[a]
Tiempo	Esfericidad asumida	,060	2	,030	1,793	,174	,052	3,586	,362
	Greenhouse-Geisser	,060	1,588	,038	1,793	,183	,052	2,847	,320
	Huynh-Feldt	,060	1,655	,036	1,793	,181	,052	2,967	,327
	Límite-inferior	,060	1,000	,060	1,793	,190	,052	1,793	,255
Error(Tiempo)	Esfericidad asumida	1,096	66	,017					
	Greenhouse-Geisser	1,096	52,394	,021					
	Huynh-Feldt	1,096	54,608	,020					
	Límite-inferior	1,096	33,000	,033					

a. Calculado con alfa = ,05

Comparaciones por pares

Medida:MEASURE_1

(I)Tiempo	(J)Tiempo	Diferencia de medias (I-J)	Error típ.	Sig.[a]	Intervalo de confianza al 95 % para la diferencia[a]	
					Límite inferior	Límite superior
1	2	,055	,024	,083	-,005	,115
	3	,047	,038	,679	-,049	,142
2	1	-,055	,024	,083	-,115	,005
	3	-,008	,031	1,000	-,086	,069
3	1	-,047	,038	,679	-,142	,049
	2	,008	,031	1,000	-,069	,086

Basadas en las medias marginales estimadas.

a. Ajuste para comparaciones múltiples: Bonferroni.

MAXIMUM TAKEOFF VELOCITY

Factores intra-sujetos

Medida:MEASURE_1

Tiempo	Variable dependiente
1	G_CMJ_Media_Pre_Vmáx
2	G_CMJ1_min10_Vmáx
3	G_CMJ_Media_Post_Vmax

Estadísticos descriptivos

	Media	Desviación típica	N
G_CMJ_Media_Pre_Vmáx	2,8863	,14675	34
G_CMJ1_min10_Vmáx	2,8116	,17144	34
G_CMJ_Media_Post_Vmax	2,8074	,15424	34

Contrastes multivariados[c]

Efecto		Valor	F	Gl de la hipótesis	Gl del error	Sig.	Eta al cuadrado parcial	Parámetro de no centralidad Parámetro	Potencia observada[b]
Tiempo	Traza de Pillai	,558	20,229[a]	2,000	32,000	,000	,558	40,458	1,000
	Lambda de Wilks	,442	20,229[a]	2,000	32,000	,000	,558	40,458	1,000
	Traza de Hotelling	1,264	20,229[a]	2,000	32,000	,000	,558	40,458	1,000
	Raíz mayor de Roy	1,264	20,229[a]	2,000	32,000	,000	,558	40,458	1,000

a. Estadístico exacto

b. Calculado con alfa = ,05

c. Diseño: Intersección

Diseño intra-sujetos: Tiempo

Prueba de esfericidad de Mauchly[b]

Medida:MEASURE_1

Efecto intra-sujetos	W de Mauchly	Chi-cuadrado aprox.	gl	Sig.	Epsilon[a]		
					Greenhouse-Geisser	Huynh-Feldt	Límite-inferior
Tiempo	,651	13,714	2	,001	,742	,768	,500

Contrasta la hipótesis nula de que la matriz de covarianza error de las variables dependientes transformadas es proporcional a una matriz identidad.

a. Puede usarse para corregir los grados de libertad en las pruebas de significación promediadas. Las pruebas corregidas se muestran en la tabla Pruebas de los efectos inter-sujetos.

b. Diseño: Intersección

Diseño intra-sujetos: Tiempo

Pruebas de efectos intra-sujetos.

Medida:MEASURE_1

Origen		Suma de cuadrados tipo III	gl	Media cuadrática	F	Sig.	Eta al cuadrado parcial	Parámetro de no centralidad Parámetro	Potencia observada[a]
Tiempo	Esfericidad asumida	,134	2	,067	20,808	,000	,387	41,616	1,000
	Greenhouse-Geisser	,134	1,483	,090	20,808	,000	,387	30,860	,999
	Huynh-Feldt	,134	1,536	,087	20,808	,000	,387	31,970	1,000
	Límite-inferior	,134	1,000	,134	20,808	,000	,387	20,808	,993
Error(Tiempo)	Esfericidad asumida	,212	66	,003					
	Greenhouse-Geisser	,212	48,942	,004					
	Huynh-Feldt	,212	50,703	,004					
	Límite-inferior	,212	33,000	,006					

a. Calculado con alfa = ,05

Comparaciones por pares

Medida:MEASURE_1

(I)Tiempo	(J)Tiempo	Diferencia de medias (I-J)	Error típ.	Sig.[a]	Intervalo de confianza al 95 % para la diferencia[a]	
					Límite inferior	Límite superior
1	2	,075*	,017	,000	,031	,118
	3	,079*	,012	,000	,048	,110
2	1	-,075*	,017	,000	-,118	-,031
	3	,004	,011	1,000	-,023	,031
3	1	-,079*	,012	,000	-,110	-,048
	2	-,004	,011	1,000	-,031	,023

Basadas en las medias marginales estimadas.

*. La diferencia de medias es significativa al nivel ,05.

a. Ajuste para comparaciones múltiples: Bonferroni.

PEAK RATE OF VELOCITY

Factores intra-sujetos

Medida:MEASURE_1

Tiempo	Variable dependiente
1	G_CMJ_Med_Pre_RVD
2	G_CMJ_10_RVD
3	G_CMJ_Media_Post_RVD

Estadísticos descriptivos

	Grupos	Media	Desviación típica	N
G_CMJ_Med_Pre_RVD	Gimnástica	4,1681	,55084	34
	Total	4,1681	,55084	34
G_CMJ_10_RVD	Gimnástica	4,1337	,84527	34
	Total	4,1337	,84527	34
G_CMJ_Media_Post_RVD	Gimnástica	4,0632	,62783	34
	Total	4,0632	,62783	34

Contrastes multivariados[c]

Efecto		Valor	F	Gl de la hipótesis	Gl del error	Sig.	Eta al cuadrado parcial	Parámetro de no centralidad Parámetro	Potencia observada[b]
Tiempo	Traza de Pillai	,035	,588[a]	2,000	32,000	,561	,035	1,176	,139
	Lambda de Wilks	,965	,588[a]	2,000	32,000	,561	,035	1,176	,139
	Traza de Hotelling	,037	,588[a]	2,000	32,000	,561	,035	1,176	,139
	Raíz mayor de Roy	,037	,588[a]	2,000	32,000	,561	,035	1,176	,139
Tiempo * Grupos	Traza de Pillai	,000	.[a]	,000	,000	.	.	.	.
	Lambda de Wilks	1,000	.[a]	,000	32,500	.	.	.	.
	Traza de Hotelling	,000	.[a]	,000	2,000	.	.	.	.
	Raíz mayor de Roy	,000	,000[a]	2,000	31,000	1,000	,000	,000	,050

a. Estadístico exacto

b. Calculado con alfa = ,05

c. Diseño: Intersección + Grupos

Diseño intra-sujetos: Tiempo

Prueba de esfericidad de Mauchly[b]

Medida:MEASURE_1

Efecto intra-sujetos	W de Mauchly	Chi-cuadrado aprox.	gl	Sig.	Epsilon[a]		
					Greenhouse-Geisser	Huynh-Feldt	Límite-inferior
_ Tiempo	,930	2,338	2	,311	,934	,988	,500

Contrasta la hipótesis nula de que la matriz de covarianza error de las variables dependientes transformadas es proporcional a una matriz identidad.

a. Puede usarse para corregir los grados de libertad en las pruebas de significación promediadas. Las pruebas corregidas se muestran en la tabla Pruebas de los efectos inter-sujetos.

b. Diseño: Intersección + Grupos

Diseño intra-sujetos: Tiempo

Pruebas de efectos intra-sujetos.

Medida:MEASURE_1

Origen		Suma de cuadrados tipo III	gl	Media cuadrática	F	Sig.	Eta al cuadrado parcial	Parámetro de no centralidad Parámetro	Potencia observada[a]
Tiempo	Esfericidad asumida	,195	2	,097	,458	,634	,014	,917	,122
	Greenhouse-Geisser	,195	1,868	,104	,458	,621	,014	,856	,119
	Huynh-Feldt	,195	1,976	,098	,458	,632	,014	,906	,121
	Límite-inferior	,195	1,000	,195	,458	,503	,014	,458	,101
Tiempo * Grupos	Esfericidad asumida	,000	0	.	.	.	,000	,000	.
	Greenhouse-Geisser	,000	,000	.	.	.	,000	,000	.
	Huynh-Feldt	,000	,000	.	.	.	,000	,000	.
	Límite-inferior	,000	,000	.	.	.	,000	,000	.
Error(Tiempo)	Esfericidad asumida	14,006	66	,212					
	Greenhouse-Geisser	14,006	61,657	,227					
	Huynh-Feldt	14,006	65,218	,215					
	Límite-inferior	14,006	33,000	,424					

a. Calculado con alfa = ,05

Comparaciones por pares

Medida:MEASURE_1

(I)Tiempo	(J)Tiempo	Diferencia de medias (I-J)	Error típ.	Sig.[a]	Intervalo de confianza al 95 % para la diferencia[a]	
					Límite inferior	Límite superior
1	2	,034	,117	1,000	-,261	,330
	3	,105	,096	,846	-,137	,347
2	1	-,034	,117	1,000	-,330	,261
	3	,071	,120	1,000	-,233	,374
3	1	-,105	,096	,846	-,347	,137
	2	-,071	,120	1,000	-,374	,233

Basadas en las medias marginales estimadas.

a. Ajuste para comparaciones múltiples: Bonferroni.
