# Supplementary material for: Muscular fatigue in response to different modalities of CrossFit sessions
Source: PLoS One. 2017 Jul 28;12(7):e0181855. doi: 10.1371/journal.pone.0181855 (PMC5533437; doi:10.1371/journal.pone.0181855)
Supplement: S5 File — Statistical analysis performed with mechanical variables in Metabolic WOD. (PDF) [file pone.0181855.s005.pdf]

# JUMP HEIGHT

**Factores intra-sujetos**

Medida:MEASURE_1

| Tiempo | Variable dependiente |
|---|---|
| 1 | M_CMJ_Media_Pre_AlturaVuelo |
| 2 | M_CMJ_Serie2_AlturaVuelo |
| 3 | M_CMJ_Serie4_AlturaVuelo |
| 4 | M_CMJ_Serie6_AlturaVuelo |
| 5 | M_CMJ_Serie8_AlturaVuelo |
| 6 | M_CMJ_Media_Post_AlturaVuelo |

**Estadísticos descriptivos**

| | Media | Desviación típica | N |
|---|---|---|---|
| M_CMJ_Media_Pre_AlturaVuelo | 37,0629 | 4,28916 | 34 |
| M_CMJ_Serie2_AlturaVuelo | 34,1189 | 4,37389 | 34 |
| M_CMJ_Serie4_AlturaVuelo | 33,8488 | 4,63791 | 34 |
| M_CMJ_Serie6_AlturaVuelo | 33,5160 | 4,63503 | 34 |
| M_CMJ_Serie8_AlturaVuelo | 34,2430 | 4,54842 | 34 |
| M_CMJ_Media_Post_AlturaVuelo | 35,7494 | 5,34102 | 34 |

**Contrastes multivariados[c]**

| Efecto | | Valor | F | Gl de la hipótesis | Gl del error | Sig. | Eta al cuadrado parcial | Parámetro de no centralidad Parámetro | Potencia observada[b] |
|---|---|---|---|---|---|---|---|---|---|
| Tiempo | Traza de Pillai | ,742 | 16,639[a] | 5,000 | 29,000 | ,000 | ,742 | 83,195 | 1,000 |
| | Lambda de Wilks | ,258 | 16,639[a] | 5,000 | 29,000 | ,000 | ,742 | 83,195 | 1,000 |
| | Traza de Hotelling | 2,869 | 16,639[a] | 5,000 | 29,000 | ,000 | ,742 | 83,195 | 1,000 |
| | Raíz mayor de Roy | 2,869 | 16,639[a] | 5,000 | 29,000 | ,000 | ,742 | 83,195 | 1,000 |

a. Estadístico exacto

b. Calculado con alfa = ,05

c. Diseño: Intersección

Diseño intra-sujetos: Tiempo

**Prueba de esfericidad de Mauchly[b]**

Medida:MEASURE_1

| Efecto intra-sujetos | W de Mauchly | Chi-cuadrado aprox. | gl | Sig. | Epsilon[a] | | |
|---|---|---|---|---|---|---|---|
| | | | | | Greenhouse-Geisser | Huynh-Feldt | Límite-inferior |
| Tiempo | ,184 | 52,657 | 14 | ,000 | ,581 | ,643 | ,200 |

Contrasta la hipótesis nula de que la matriz de covarianza error de las variables dependientes transformadas es proporcional a una matriz identidad.

a. Puede usarse para corregir los grados de libertad en las pruebas de significación promediadas. Las pruebas corregidas se muestran en la tabla Pruebas de los efectos inter-sujetos.

b. Diseño: Intersección

Diseño intra-sujetos: Tiempo

**Pruebas de efectos intra-sujetos.**

Medida:MEASURE_1

| Origen | | Suma de cuadrados tipo III | gl | Media cuadrática | F | Sig. | Eta al cuadrado parcial | Parámetro de no centralidad Parámetro | Potencia observada[a] |
|---|---|---|---|---|---|---|---|---|---|
| Tiempo | Esfericidad asumida | 317,503 | 5 | 63,501 | 14,966 | ,000 | ,312 | 74,828 | 1,000 |
| | Greenhouse-Geisser | 317,503 | 2,903 | 109,382 | 14,966 | ,000 | ,312 | 43,441 | 1,000 |
| | Huynh-Feldt | 317,503 | 3,213 | 98,829 | 14,966 | ,000 | ,312 | 48,079 | 1,000 |
| | Límite-inferior | 317,503 | 1,000 | 317,503 | 14,966 | ,000 | ,312 | 14,966 | ,964 |
| Error(Tiempo) | Esfericidad asumida | 700,113 | 165 | 4,243 | | | | | |
| | Greenhouse-Geisser | 700,113 | 95,789 | 7,309 | | | | | |
| | Huynh-Feldt | 700,113 | 106,017 | 6,604 | | | | | |
| | Límite-inferior | 700,113 | 33,000 | 21,216 | | | | | |

a. Calculado con alfa = ,05

**Comparaciones por pares**

Medida:MEASURE_1

| (I)Tiempo | (J)Tiempo | Diferencia de medias (I-J) | Error típ. | Sig.[a] | Intervalo de confianza al 95 % para la diferencia[a] | |
|---|---|---|---|---|---|---|
| | | | | | Límite inferior | Límite superior |
| 1 | 2 | 2,944* | ,325 | ,000 | 1,917 | 3,971 |
| | 3 | 3,214* | ,386 | ,000 | 1,992 | 4,436 |
| | 4 | 3,547* | ,486 | ,000 | 2,008 | 5,086 |
| | 5 | 2,820* | ,532 | ,000 | 1,135 | 4,505 |
| | 6 | 1,314 | ,653 | ,787 | -,753 | 3,380 |
| 2 | 1 | -2,944* | ,325 | ,000 | -3,971 | -1,917 |
| | 3 | ,270 | ,292 | 1,000 | -,654 | 1,194 |
| | 4 | ,603 | ,421 | 1,000 | -,730 | 1,936 |
| | 5 | -,124 | ,521 | 1,000 | -1,772 | 1,524 |
| | 6 | -1,630 | ,684 | ,346 | -3,795 | ,534 |
| 3 | 1 | -3,214* | ,386 | ,000 | -4,436 | -1,992 |
| | 2 | -,270 | ,292 | 1,000 | -1,194 | ,654 |
| | 4 | ,333 | ,348 | 1,000 | -,768 | 1,434 |
| | 5 | -,394 | ,440 | 1,000 | -1,786 | ,998 |
| | 6 | -1,901 | ,660 | ,104 | -3,988 | ,186 |
| 4 | 1 | -3,547* | ,486 | ,000 | -5,086 | -2,008 |
| | 2 | -,603 | ,421 | 1,000 | -1,936 | ,730 |
| | 3 | -,333 | ,348 | 1,000 | -1,434 | ,768 |
| | 5 | -,727 | ,386 | 1,000 | -1,948 | ,494 |
| | 6 | -2,233* | ,606 | ,012 | -4,152 | -,315 |
| 5 | 1 | -2,820* | ,532 | ,000 | -4,505 | -1,135 |
| | 2 | ,124 | ,521 | 1,000 | -1,524 | 1,772 |
| | 3 | ,394 | ,440 | 1,000 | -,998 | 1,786 |
| | 4 | ,727 | ,386 | 1,000 | -,494 | 1,948 |
| | 6 | -1,506 | ,524 | ,106 | -3,165 | ,153 |
| 6 | 1 | -1,314 | ,653 | ,787 | -3,380 | ,753 |
| | 2 | 1,630 | ,684 | ,346 | -,534 | 3,795 |
| | 3 | 1,901 | ,660 | ,104 | -,186 | 3,988 |
| | 4 | 2,233* | ,606 | ,012 | ,315 | 4,152 |
| | 5 | 1,506 | ,524 | ,106 | -,153 | 3,165 |

Basadas en las medias marginales estimadas.

*. La diferencia de medias es significativa al nivel ,05.

a. Ajuste para comparaciones múltiples: Bonferroni.

# PEAK POWER RELATIVE

**Factores intra-sujetos**

Medida:MEASURE_1

| Tiempo | Variable dependiente |
|---|---|
| 1 | M_CMJ_Med_Pre_Pmax_rel |
| 2 | M_CMJ_Serie2_Pmax_rel |
| 3 | M_CMJ_Serie4_Pmax_rel |
| 4 | M_CMJ_Serie6_Pmax_rel |
| 5 | M_CMJ_Serie8_Pmax_rel |
| 6 | M_CMJ_Med_Post_Pmax_rel |

**Estadísticos descriptivos**

| | Media | Desviación típica | N |
|---|---|---|---|
| M_CMJ_Med_Pre_Pmax_rel | 55,5822 | 6,11866 | 34 |
| M_CMJ_Serie2_Pmax_rel | 51,6498 | 6,06821 | 34 |
| M_CMJ_Serie4_Pmax_rel | 51,8496 | 6,34616 | 34 |
| M_CMJ_Serie6_Pmax_rel | 51,5019 | 5,75215 | 34 |
| M_CMJ_Serie8_Pmax_rel | 52,8792 | 5,97768 | 34 |
| M_CMJ_Med_Post_Pmax_rel | 55,9259 | 6,97055 | 34 |

**Contrastes multivariados[c]**

| Efecto | | Valor | F | Gl de la hipótesis | Gl del error | Sig. | Eta al cuadrado parcial | Parámetro de no centralidad Parámetro | Potencia observada[b] |
|---|---|---|---|---|---|---|---|---|---|
| Tiempo | Traza de Pillai | ,811 | 24,860[a] | 5,000 | 29,000 | ,000 | ,811 | 124,302 | 1,000 |
| | Lambda de Wilks | ,189 | 24,860[a] | 5,000 | 29,000 | ,000 | ,811 | 124,302 | 1,000 |
| | Traza de Hotelling | 4,286 | 24,860[a] | 5,000 | 29,000 | ,000 | ,811 | 124,302 | 1,000 |
| | Raíz mayor de Roy | 4,286 | 24,860[a] | 5,000 | 29,000 | ,000 | ,811 | 124,302 | 1,000 |

a. Estadístico exacto

b. Calculado con alfa = ,05

c. Diseño: Intersección

Diseño intra-sujetos: Tiempo

**Prueba de esfericidad de Mauchly[b]**

Medida:MEASURE_1

| Efecto intra-sujetos | W de Mauchly | Chi-cuadrado aprox. | gl | Sig. | Epsilon[a] | | |
|---|---|---|---|---|---|---|---|
| | | | | | Greenhouse-Geisser | Huynh-Feldt | Límite-inferior |
| Tiempo | ,317 | 35,700 | 14 | ,001 | ,701 | ,794 | ,200 |

Contrasta la hipótesis nula de que la matriz de covarianza error de las variables dependientes transformadas es proporcional a una matriz identidad.

a. Puede usarse para corregir los grados de libertad en las pruebas de significación promediadas. Las pruebas corregidas se muestran en la tabla Pruebas de los efectos inter-sujetos.

b. Diseño: Intersección

Diseño intra-sujetos: Tiempo

**Pruebas de efectos intra-sujetos.**

Medida:MEASURE_1

| Origen | | Suma de cuadrados tipo III | gl | Media cuadrática | F | Sig. | Eta al cuadrado parcial | Parámetro de no centralidad Parámetro | Potencia observada[a] |
|---|---|---|---|---|---|---|---|---|---|
| Tiempo | Esfericidad asumida | 690,626 | 5 | 138,125 | 32,697 | ,000 | ,498 | 163,484 | 1,000 |
| | Greenhouse-Geisser | 690,626 | 3,504 | 197,122 | 32,697 | ,000 | ,498 | 114,555 | 1,000 |
| | Huynh-Feldt | 690,626 | 3,971 | 173,932 | 32,697 | ,000 | ,498 | 129,828 | 1,000 |
| | Límite-inferior | 690,626 | 1,000 | 690,626 | 32,697 | ,000 | ,498 | 32,697 | 1,000 |
| Error(Tiempo) | Esfericidad asumida | 697,029 | 165 | 4,224 | | | | | |
| | Greenhouse-Geisser | 697,029 | 115,617 | 6,029 | | | | | |
| | Huynh-Feldt | 697,029 | 131,032 | 5,320 | | | | | |
| | Límite-inferior | 697,029 | 33,000 | 21,122 | | | | | |

a. Calculado con alfa = ,05

**Comparaciones por pares**

Medida:MEASURE_1

| (I)Tiempo | (J)Tiempo | Diferencia de medias (I-J) | Error típ. | Sig.[a] | Intervalo de confianza al 95 % para la diferencia[a] | |
|---|---|---|---|---|---|---|
| | | | | | Límite inferior | Límite superior |
| 1 | 2 | 3,932* | ,477 | ,000 | 2,422 | 5,442 |
| | 3 | 3,733* | ,453 | ,000 | 2,299 | 5,166 |
| | 4 | 4,080* | ,562 | ,000 | 2,301 | 5,859 |
| | 5 | 2,703* | ,686 | ,006 | ,532 | 4,874 |
| | 6 | -,344 | ,462 | 1,000 | -1,805 | 1,117 |
| 2 | 1 | -3,932* | ,477 | ,000 | -5,442 | -2,422 |
| | 3 | -,200 | ,313 | 1,000 | -1,190 | ,790 |
| | 4 | ,148 | ,456 | 1,000 | -1,294 | 1,590 |
| | 5 | -1,229 | ,626 | ,872 | -3,211 | ,753 |
| | 6 | -4,276* | ,513 | ,000 | -5,899 | -2,653 |
| 3 | 1 | -3,733* | ,453 | ,000 | -5,166 | -2,299 |
| | 2 | ,200 | ,313 | 1,000 | -,790 | 1,190 |
| | 4 | ,348 | ,348 | 1,000 | -,752 | 1,448 |
| | 5 | -1,030 | ,543 | 1,000 | -2,748 | ,689 |
| | 6 | -4,076* | ,398 | ,000 | -5,334 | -2,818 |
| 4 | 1 | -4,080* | ,562 | ,000 | -5,859 | -2,301 |
| | 2 | -,148 | ,456 | 1,000 | -1,590 | 1,294 |
| | 3 | -,348 | ,348 | 1,000 | -1,448 | ,752 |
| | 5 | -1,377 | ,484 | ,114 | -2,910 | ,155 |
| | 6 | -4,424* | ,443 | ,000 | -5,826 | -3,022 |
| 5 | 1 | -2,703* | ,686 | ,006 | -4,874 | -,532 |
| | 2 | 1,229 | ,626 | ,872 | -,753 | 3,211 |
| | 3 | 1,030 | ,543 | 1,000 | -,689 | 2,748 |
| | 4 | 1,377 | ,484 | ,114 | -,155 | 2,910 |
| | 6 | -3,047* | ,573 | ,000 | -4,861 | -1,233 |
| 6 | 1 | ,344 | ,462 | 1,000 | -1,117 | 1,805 |
| | 2 | 4,276* | ,513 | ,000 | 2,653 | 5,899 |
| | 3 | 4,076* | ,398 | ,000 | 2,818 | 5,334 |
| | 4 | 4,424* | ,443 | ,000 | 3,022 | 5,826 |
| | 5 | 3,047* | ,573 | ,000 | 1,233 | 4,861 |

Basadas en las medias marginales estimadas.

*. La diferencia de medias es significativa al nivel ,05.

a. Ajuste para comparaciones múltiples: Bonferroni.

# PEAK POWER TOTAL

**Factores intra-sujetos**

Medida:MEASURE_1

| Tiempo | Variable dependiente |
|---|---|
| 1 | M_CMJ_Med_Pre_Pmax_total |
| 2 | M_CMJ_Serie2_Pmax_total |
| 3 | M_CMJ_Serie4_Pmax_total |
| 4 | M_CMJ_Serie6_Pmax_total |
| 5 | M_CMJ_Serie8_Pmax_total |
| 6 | M_CMJ_Med_Post_Pmax_total |

**Estadísticos descriptivos**

| | Media | Desviación típica | N |
|---|---|---|---|
| M_CMJ_Med_Pre_Pmax_total | 4277,3436 | 553,10549 | 34 |
| M_CMJ_Serie2_Pmax_total | 3971,1776 | 546,13764 | 34 |
| M_CMJ_Serie4_Pmax_total | 3984,7196 | 552,00030 | 34 |
| M_CMJ_Serie6_Pmax_total | 3960,1380 | 521,78267 | 34 |
| M_CMJ_Serie8_Pmax_total | 3980,2014 | 528,12338 | 34 |
| M_CMJ_Med_Post_Pmax_total | 4301,3845 | 633,19611 | 34 |

**Contrastes multivariados[c]**

| Efecto | | Valor | F | Gl de la hipótesis | Gl del error | Sig. | Eta al cuadrado parcial | Parámetro de no centralidad Parámetro | Potencia observada[b] |
|---|---|---|---|---|---|---|---|---|---|
| Tiempo | Traza de Pillai | ,803 | 23,706[a] | 5,000 | 29,000 | ,000 | ,803 | 118,529 | 1,000 |
| | Lambda de Wilks | ,197 | 23,706[a] | 5,000 | 29,000 | ,000 | ,803 | 118,529 | 1,000 |
| | Traza de Hotelling | 4,087 | 23,706[a] | 5,000 | 29,000 | ,000 | ,803 | 118,529 | 1,000 |
| | Raíz mayor de Roy | 4,087 | 23,706[a] | 5,000 | 29,000 | ,000 | ,803 | 118,529 | 1,000 |

a. Estadístico exacto

b. Calculado con alfa = ,05

c. Diseño: Intersección

Diseño intra-sujetos: Tiempo

**Prueba de esfericidad de Mauchly[b]**

Medida:MEASURE_1

| Efecto intra-sujetos | W de Mauchly | Chi-cuadrado aprox. | gl | Sig. | Epsilon[a] | | |
|---|---|---|---|---|---|---|---|
| | | | | | Greenhouse-Geisser | Huynh-Feldt | Límite-inferior |
| Tiempo | ,273 | 40,390 | 14 | ,000 | ,655 | ,735 | ,200 |

Contrasta la hipótesis nula de que la matriz de covarianza error de las variables dependientes transformadas es proporcional a una matriz identidad.

a. Puede usarse para corregir los grados de libertad en las pruebas de significación promediadas. Las pruebas corregidas se muestran en la tabla Pruebas de los efectos inter-sujetos.

b. Diseño: Intersección

Diseño intra-sujetos: Tiempo

**Pruebas de efectos intra-sujetos.**

Medida:MEASURE_1

| Origen | | Suma de cuadrados tipo III | gl | Media cuadrática | F | Sig. | Eta al cuadrado parcial | Parámetro de no centralidad Parámetro | Potencia observada[a] |
|---|---|---|---|---|---|---|---|---|---|
| Tiempo | Esfericidad asumida | 4528756,111 | 5 | 905751,222 | 34,913 | ,000 | ,514 | 174,567 | 1,000 |
| | Greenhouse-Geisser | 4528756,111 | 3,273 | 1383669,868 | 34,913 | ,000 | ,514 | 114,272 | 1,000 |
| | Huynh-Feldt | 4528756,111 | 3,676 | 1231915,389 | 34,913 | ,000 | ,514 | 128,348 | 1,000 |
| | Límite-inferior | 4528756,111 | 1,000 | 4528756,111 | 34,913 | ,000 | ,514 | 34,913 | 1,000 |
| Error(Tiempo) | Esfericidad asumida | 4280567,703 | 165 | 25942,835 | | | | | |
| | Greenhouse-Geisser | 4280567,703 | 108,009 | 39631,543 | | | | | |
| | Huynh-Feldt | 4280567,703 | 121,314 | 35284,940 | | | | | |
| | Límite-inferior | 4280567,703 | 33,000 | 129714,173 | | | | | |

a. Calculado con alfa = ,05

**Comparaciones por pares**

Medida:MEASURE_1

| (I)Tiempo | (J)Tiempo | Diferencia de medias (I-J) | Error típ. | Sig.[a] | Intervalo de confianza al 95 % para la diferencia[a] | |
|---|---|---|---|---|---|---|
| | | | | | Límite inferior | Límite superior |
| 1 | 2 | 306,166* | 36,583 | ,000 | 190,410 | 421,922 |
| | 3 | 292,624* | 34,359 | ,000 | 183,908 | 401,340 |
| | 4 | 317,206* | 41,116 | ,000 | 187,108 | 447,303 |
| | 5 | 297,142* | 56,235 | ,000 | 119,206 | 475,079 |
| | 6 | -24,041 | 33,416 | 1,000 | -129,774 | 81,692 |
| 2 | 1 | -306,166* | 36,583 | ,000 | -421,922 | -190,410 |
| | 3 | -13,542 | 23,544 | 1,000 | -88,040 | 60,956 |
| | 4 | 11,040 | 34,236 | 1,000 | -97,287 | 119,366 |
| | 5 | -9,024 | 47,453 | 1,000 | -159,174 | 141,126 |
| | 6 | -330,207* | 39,713 | ,000 | -455,863 | -204,551 |
| 3 | 1 | -292,624* | 34,359 | ,000 | -401,340 | -183,908 |
| | 2 | 13,542 | 23,544 | 1,000 | -60,956 | 88,040 |
| | 4 | 24,582 | 26,962 | 1,000 | -60,730 | 109,893 |
| | 5 | 4,518 | 39,716 | 1,000 | -121,150 | 130,186 |
| | 6 | -316,665* | 32,353 | ,000 | -419,036 | -214,294 |
| 4 | 1 | -317,206* | 41,116 | ,000 | -447,303 | -187,108 |
| | 2 | -11,040 | 34,236 | 1,000 | -119,366 | 97,287 |
| | 3 | -24,582 | 26,962 | 1,000 | -109,893 | 60,730 |
| | 5 | -20,063 | 40,643 | 1,000 | -148,663 | 108,537 |
| | 6 | -341,247* | 35,107 | ,000 | -452,329 | -230,164 |
| 5 | 1 | -297,142* | 56,235 | ,000 | -475,079 | -119,206 |
| | 2 | 9,024 | 47,453 | 1,000 | -141,126 | 159,174 |
| | 3 | -4,518 | 39,716 | 1,000 | -130,186 | 121,150 |
| | 4 | 20,063 | 40,643 | 1,000 | -108,537 | 148,663 |
| | 6 | -321,183* | 51,104 | ,000 | -482,883 | -159,483 |
| 6 | 1 | 24,041 | 33,416 | 1,000 | -81,692 | 129,774 |
| | 2 | 330,207* | 39,713 | ,000 | 204,551 | 455,863 |
| | 3 | 316,665* | 32,353 | ,000 | 214,294 | 419,036 |
| | 4 | 341,247* | 35,107 | ,000 | 230,164 | 452,329 |
| | 5 | 321,183* | 51,104 | ,000 | 159,483 | 482,883 |

Basadas en las medias marginales estimadas.

*. La diferencia de medias es significativa al nivel ,05.

a. Ajuste para comparaciones múltiples: Bonferroni.

# AVERAGE POWER RELATIVE

**Factores intra-sujetos**

Medida:MEASURE_1

| Tiempo | Variable dependiente |
|---|---|
| 1 | M_CMJ_Med_Pre_Pmed_rel |
| 2 | M_CMJ_Serie2_Pmed_rel |
| 3 | M_CMJ_Serie4_Pmed_rel |
| 4 | M_CMJ_Serie6_Pmed_rel |
| 5 | M_CMJ_Serie8_Pmed_rel |
| 6 | M_CMJ_Med_Post_Pmed_rel |

**Estadísticos descriptivos**

| | Media | Desviación típica | N |
|---|---|---|---|
| M_CMJ_Med_Pre_Pmed_rel | 31,8384 | 3,53315 | 34 |
| M_CMJ_Serie2_Pmed_rel | 29,9909 | 3,42852 | 34 |
| M_CMJ_Serie4_Pmed_rel | 29,5936 | 3,41143 | 34 |
| M_CMJ_Serie6_Pmed_rel | 29,3333 | 3,36179 | 34 |
| M_CMJ_Serie8_Pmed_rel | 29,5870 | 3,69457 | 34 |
| M_CMJ_Med_Post_Pmed_rel | 31,1874 | 3,94928 | 34 |

**Contrastes multivariados[c]**

| Efecto | | Valor | F | Gl de la hipótesis | Gl del error | Sig. | Eta al cuadrado parcial | Parámetro de no centralidad Parámetro | Potencia observada[b] |
|---|---|---|---|---|---|---|---|---|---|
| Tiempo | Traza de Pillai | ,653 | 10,896[a] | 5,000 | 29,000 | ,000 | ,653 | 54,478 | 1,000 |
| | Lambda de Wilks | ,347 | 10,896[a] | 5,000 | 29,000 | ,000 | ,653 | 54,478 | 1,000 |
| | Traza de Hotelling | 1,879 | 10,896[a] | 5,000 | 29,000 | ,000 | ,653 | 54,478 | 1,000 |
| | Raíz mayor de Roy | 1,879 | 10,896[a] | 5,000 | 29,000 | ,000 | ,653 | 54,478 | 1,000 |

a. Estadístico exacto

b. Calculado con alfa = ,05

c. Diseño: Intersección

Diseño intra-sujetos: Tiempo

**Prueba de esfericidad de Mauchly[b]**

Medida:MEASURE_1

| Efecto intra-sujetos | W de Mauchly | Chi-cuadrado aprox. | gl | Sig. | Epsilon[a] Greenhouse-Geisser | Huynh-Feldt | Límite-inferior |
|---|---|---|---|---|---|---|---|
| Tiempo | ,422 | 26,846 | 14 | ,020 | ,765 | ,878 | ,200 |

Contrasta la hipótesis nula de que la matriz de covarianza error de las variables dependientes transformadas es proporcional a una matriz identidad.

a. Puede usarse para corregir los grados de libertad en las pruebas de significación promediadas. Las pruebas corregidas se muestran en la tabla Pruebas de los efectos inter-sujetos.

b. Diseño: Intersección

Diseño intra-sujetos: Tiempo

**Pruebas de efectos intra-sujetos.**

Medida:MEASURE_1

| Origen | | Suma de cuadrados tipo III | gl | Media cuadrática | F | Sig. | Eta al cuadrado parcial | Parámetro de no centralidad Parámetro | Potencia observada[a] |
|---|---|---|---|---|---|---|---|---|---|
| Tiempo | Esfericidad asumida | 176,096 | 5 | 35,219 | 15,085 | ,000 | ,314 | 75,425 | 1,000 |
| | Greenhouse-Geisser | 176,096 | 3,826 | 46,020 | 15,085 | ,000 | ,314 | 57,723 | 1,000 |
| | Huynh-Feldt | 176,096 | 4,391 | 40,104 | 15,085 | ,000 | ,314 | 66,239 | 1,000 |
| | Límite-inferior | 176,096 | 1,000 | 176,096 | 15,085 | ,000 | ,314 | 15,085 | ,965 |
| Error(Tiempo) | Esfericidad asumida | 385,227 | 165 | 2,335 | | | | | |
| | Greenhouse-Geisser | 385,227 | 126,274 | 3,051 | | | | | |
| | Huynh-Feldt | 385,227 | 144,903 | 2,659 | | | | | |
| | Límite-inferior | 385,227 | 33,000 | 11,674 | | | | | |

a. Calculado con alfa = ,05

**Comparaciones por pares**

Medida:MEASURE_1

| (I)Tiempo | (J)Tiempo | Diferencia de medias (I-J) | Error típ. | Sig.[a] | Intervalo de confianza al 95 % para la diferencia[a] | |
|---|---|---|---|---|---|---|
| | | | | | Límite inferior | Límite superior |
| 1 | 2 | 1,847* | ,296 | ,000 | ,910 | 2,785 |
| | 3 | 2,245* | ,359 | ,000 | 1,109 | 3,380 |
| | 4 | 2,505* | ,369 | ,000 | 1,338 | 3,673 |
| | 5 | 2,251* | ,468 | ,000 | ,772 | 3,731 |
| | 6 | ,651 | ,356 | 1,000 | -,476 | 1,778 |
| 2 | 1 | -1,847* | ,296 | ,000 | -2,785 | -,910 |
| | 3 | ,397 | ,265 | 1,000 | -,442 | 1,237 |
| | 4 | ,658 | ,316 | ,675 | -,341 | 1,656 |
| | 5 | ,404 | ,451 | 1,000 | -1,023 | 1,831 |
| | 6 | -1,196 | ,388 | ,062 | -2,425 | ,032 |
| 3 | 1 | -2,245* | ,359 | ,000 | -3,380 | -1,109 |
| | 2 | -,397 | ,265 | 1,000 | -1,237 | ,442 |
| | 4 | ,260 | ,299 | 1,000 | -,684 | 1,205 |
| | 5 | ,007 | ,363 | 1,000 | -1,143 | 1,156 |
| | 6 | -1,594* | ,413 | ,007 | -2,900 | -,287 |
| 4 | 1 | -2,505* | ,369 | ,000 | -3,673 | -1,338 |
| | 2 | -,658 | ,316 | ,675 | -1,656 | ,341 |
| | 3 | -,260 | ,299 | 1,000 | -1,205 | ,684 |
| | 5 | -,254 | ,391 | 1,000 | -1,492 | ,985 |
| | 6 | -1,854* | ,344 | ,000 | -2,942 | -,766 |
| 5 | 1 | -2,251* | ,468 | ,000 | -3,731 | -,772 |
| | 2 | -,404 | ,451 | 1,000 | -1,831 | 1,023 |
| | 3 | -,007 | ,363 | 1,000 | -1,156 | 1,143 |
| | 4 | ,254 | ,391 | 1,000 | -,985 | 1,492 |
| | 6 | -1,600* | ,418 | ,008 | -2,923 | -,278 |
| 6 | 1 | -,651 | ,356 | 1,000 | -1,778 | ,476 |
| | 2 | 1,196 | ,388 | ,062 | -,032 | 2,425 |
| | 3 | 1,594* | ,413 | ,007 | ,287 | 2,900 |
| | 4 | 1,854* | ,344 | ,000 | ,766 | 2,942 |
| | 5 | 1,600* | ,418 | ,008 | ,278 | 2,923 |

Basadas en las medias marginales estimadas.

*. La diferencia de medias es significativa al nivel ,05.

a. Ajuste para comparaciones múltiples: Bonferroni.

# AVERAGE POWER TOTAL

**Factores intra-sujetos**

Medida:MEASURE_1

| Tiempo | Variable dependiente |
|---|---|
| 1 | M_CMJ_Med_Pre_Pmed_total |
| 2 | M_CMJ_Serie2_Pmed_total |
| 3 | M_CMJ_Serie4_Pmed_total |
| 4 | M_CMJ_Serie6_Pmed_total |
| 5 | M_CMJ_Serie8_Pmed_total |
| 6 | M_CMJ_Med_Post_Pmed_total |

**Estadísticos descriptivos**

| | Media | Desviación típica | N |
|---|---|---|---|
| M_CMJ_Med_Pre_Pmed_total | 2445,2208 | 312,26043 | 34 |
| M_CMJ_Serie2_Pmed_total | 2303,8835 | 300,76988 | 34 |
| M_CMJ_Serie4_Pmed_total | 2271,7399 | 283,65807 | 34 |
| M_CMJ_Serie6_Pmed_total | 2255,0494 | 304,67673 | 34 |
| M_CMJ_Serie8_Pmed_total | 2226,9083 | 319,36734 | 34 |
| M_CMJ_Med_Post_Pmed_total | 2400,5695 | 375,02112 | 34 |

**Contrastes multivariados[c]**

| Efecto | | Valor | F | Gl de la hipótesis | Gl del error | Sig. | Eta al cuadrado parcial | Parámetro de no centralidad Parámetro | Potencia observada[b] |
|---|---|---|---|---|---|---|---|---|---|
| Tiempo | Traza de Pillai | ,634 | 10,046[a] | 5,000 | 29,000 | ,000 | ,634 | 50,230 | 1,000 |
| | Lambda de Wilks | ,366 | 10,046[a] | 5,000 | 29,000 | ,000 | ,634 | 50,230 | 1,000 |
| | Traza de Hotelling | 1,732 | 10,046[a] | 5,000 | 29,000 | ,000 | ,634 | 50,230 | 1,000 |
| | Raíz mayor de Roy | 1,732 | 10,046[a] | 5,000 | 29,000 | ,000 | ,634 | 50,230 | 1,000 |

a. Estadístico exacto

b. Calculado con alfa = ,05

c. Diseño: Intersección

Diseño intra-sujetos: Tiempo

**Prueba de esfericidad de Mauchly[b]**

Medida:MEASURE_1

| Efecto intra-sujetos | W de Mauchly | Chi-cuadrado aprox. | gl | Sig. | Epsilon[a] | | |
|---|---|---|---|---|---|---|---|
| | | | | | Greenhouse-Geisser | Huynh-Feldt | Límite-inferior |
| Tiempo | ,300 | 37,480 | 14 | ,001 | ,644 | ,722 | ,200 |

Contrasta la hipótesis nula de que la matriz de covarianza error de las variables dependientes transformadas es proporcional a una matriz identidad.

a. Puede usarse para corregir los grados de libertad en las pruebas de significación promediadas. Las pruebas corregidas se muestran en la tabla Pruebas de los efectos inter-sujetos.

b. Diseño: Intersección

Diseño intra-sujetos: Tiempo

**Pruebas de efectos intra-sujetos.**

Medida:MEASURE_1

| Origen | | Suma de cuadrados tipo III | gl | Media cuadrática | F | Sig. | Eta al cuadrado parcial | Parámetro de no centralidad Parámetro | Potencia observada[a] |
|---|---|---|---|---|---|---|---|---|---|
| Tiempo | Esfericidad asumida | 1278366,462 | 5 | 255673,292 | 17,454 | ,000 | ,346 | 87,270 | 1,000 |
| | Greenhouse-Geisser | 1278366,462 | 3,222 | 396748,824 | 17,454 | ,000 | ,346 | 56,239 | 1,000 |
| | Huynh-Feldt | 1278366,462 | 3,612 | 353942,385 | 17,454 | ,000 | ,346 | 63,040 | 1,000 |
| | Límite-inferior | 1278366,462 | 1,000 | 1278366,462 | 17,454 | ,000 | ,346 | 17,454 | ,982 |
| Error(Tiempo) | Esfericidad asumida | 2416980,200 | 165 | 14648,365 | | | | | |
| | Greenhouse-Geisser | 2416980,200 | 106,329 | 22731,047 | | | | | |
| | Huynh-Feldt | 2416980,200 | 119,189 | 20278,525 | | | | | |
| | Límite-inferior | 2416980,200 | 33,000 | 73241,824 | | | | | |

a. Calculado con alfa = ,05

**Comparaciones por pares**

Medida:MEASURE_1

| (I)Tiempo | (J)Tiempo | Diferencia de medias (I-J) | Error típ. | Sig.[a] | Intervalo de confianza al 95 % para la diferencia[a] | |
|---|---|---|---|---|---|---|
| | | | | | Límite inferior | Límite superior |
| 1 | 2 | 141,337* | 23,440 | ,000 | 67,169 | 215,505 |
| | 3 | 173,481* | 28,387 | ,000 | 83,659 | 263,303 |
| | 4 | 190,171* | 27,783 | ,000 | 102,261 | 278,082 |
| | 5 | 218,312* | 39,616 | ,000 | 92,960 | 343,664 |
| | 6 | 44,651 | 25,562 | 1,000 | -36,230 | 125,532 |
| 2 | 1 | -141,337* | 23,440 | ,000 | -215,505 | -67,169 |
| | 3 | 32,144 | 20,958 | 1,000 | -34,169 | 98,456 |
| | 4 | 48,834 | 23,633 | ,701 | -25,944 | 123,613 |
| | 5 | 76,975 | 33,454 | ,418 | -28,878 | 182,829 |
| | 6 | -96,686* | 29,849 | ,041 | -191,132 | -2,240 |
| 3 | 1 | -173,481* | 28,387 | ,000 | -263,303 | -83,659 |
| | 2 | -32,144 | 20,958 | 1,000 | -98,456 | 34,169 |
| | 4 | 16,691 | 23,262 | 1,000 | -56,914 | 90,295 |
| | 5 | 44,832 | 24,435 | 1,000 | -32,485 | 122,148 |
| | 6 | -128,830* | 32,831 | ,006 | -232,712 | -24,947 |
| 4 | 1 | -190,171* | 27,783 | ,000 | -278,082 | -102,261 |
| | 2 | -48,834 | 23,633 | ,701 | -123,613 | 25,944 |
| | 3 | -16,691 | 23,262 | 1,000 | -90,295 | 56,914 |
| | 5 | 28,141 | 31,502 | 1,000 | -71,535 | 127,817 |
| | 6 | -145,520* | 27,171 | ,000 | -231,492 | -59,548 |
| 5 | 1 | -218,312* | 39,616 | ,000 | -343,664 | -92,960 |
| | 2 | -76,975 | 33,454 | ,418 | -182,829 | 28,878 |
| | 3 | -44,832 | 24,435 | 1,000 | -122,148 | 32,485 |
| | 4 | -28,141 | 31,502 | 1,000 | -127,817 | 71,535 |
| | 6 | -173,661* | 40,255 | ,002 | -301,033 | -46,289 |
| 6 | 1 | -44,651 | 25,562 | 1,000 | -125,532 | 36,230 |
| | 2 | 96,686* | 29,849 | ,041 | 2,240 | 191,132 |
| | 3 | 128,830* | 32,831 | ,006 | 24,947 | 232,712 |
| | 4 | 145,520* | 27,171 | ,000 | 59,548 | 231,492 |
| | 5 | 173,661* | 40,255 | ,002 | 46,289 | 301,033 |

Basadas en las medias marginales estimadas.

*. La diferencia de medias es significativa al nivel ,05.

a. Ajuste para comparaciones múltiples: Bonferroni.

# MAXIMUM FORCE

**Factores intra-sujetos**

Medida:MEASURE_1

| Tiempo | Variable dependiente |
|---|---|
| 1 | M_CMJ_Media_Pre_Fmax |
| 2 | M_CMJ_Serie2_Fmax |
| 3 | M_CMJ_Serie4_Fmax |
| 4 | M_CMJ_Serie6_Fmax |
| 5 | M_CMJ_Serie8_Fmax |
| 6 | M_CMJ_Media_Post_Fmax |

**Estadísticos descriptivos**

| | Media | Desviación típica | N |
|---|---|---|---|
| M_CMJ_Media_Pre_Fmax | 2,5627 | ,31468 | 34 |
| M_CMJ_Serie2_Fmax | 2,5760 | ,34430 | 34 |
| M_CMJ_Serie4_Fmax | 2,5393 | ,25327 | 34 |
| M_CMJ_Serie6_Fmax | 2,5225 | ,32264 | 34 |
| M_CMJ_Serie8_Fmax | 2,4287 | ,24224 | 34 |
| M_CMJ_Media_Post_Fmax | 2,5426 | ,31987 | 34 |

**Contrastes multivariados[c]**

| Efecto | | Valor | F | Gl de la hipótesis | Gl del error | Sig. | Eta al cuadrado parcial | Parámetro de no centralidad Parámetro | Potencia observada[b] |
|---|---|---|---|---|---|---|---|---|---|
| Tiempo | Traza de Pillai | ,254 | 1,975[a] | 5,000 | 29,000 | ,112 | ,254 | 9,877 | ,577 |
| | Lambda de Wilks | ,746 | 1,975[a] | 5,000 | 29,000 | ,112 | ,254 | 9,877 | ,577 |
| | Traza de Hotelling | ,341 | 1,975[a] | 5,000 | 29,000 | ,112 | ,254 | 9,877 | ,577 |
| | Raíz mayor de Roy | ,341 | 1,975[a] | 5,000 | 29,000 | ,112 | ,254 | 9,877 | ,577 |

a. Estadístico exacto

b. Calculado con alfa = ,05

c. Diseño: Intersección

Diseño intra-sujetos: Tiempo

**Prueba de esfericidad de Mauchly[b]**

Medida:MEASURE_1

| Efecto intra-sujetos | W de Mauchly | Chi-cuadrado aprox. | gl | Sig. | Epsilon[a] | | |
|---|---|---|---|---|---|---|---|
| | | | | | Greenhouse-Geisser | Huynh-Feldt | Límite-inferior |
| Tiempo | ,306 | 36,848 | 14 | ,001 | ,688 | ,778 | ,200 |

Contrasta la hipótesis nula de que la matriz de covarianza error de las variables dependientes transformadas es proporcional a una matriz identidad.

a. Puede usarse para corregir los grados de libertad en las pruebas de significación promediadas. Las pruebas corregidas se muestran en la tabla Pruebas de los efectos inter-sujetos.

b. Diseño: Intersección

Diseño intra-sujetos: Tiempo

**Pruebas de efectos intra-sujetos.**

Medida:MEASURE_1

| Origen | | Suma de cuadrados tipo III | gl | Media cuadrática | F | Sig. | Eta al cuadrado parcial | Parámetro de no centralidad Parámetro | Potencia observada[a] |
|---|---|---|---|---|---|---|---|---|---|
| Tiempo | Esfericidad asumida | ,467 | 5 | ,093 | 2,986 | ,013 | ,083 | 14,930 | ,851 |
| | Greenhouse-Geisser | ,467 | 3,442 | ,136 | 2,986 | ,028 | ,083 | 10,277 | ,735 |
| | Huynh-Feldt | ,467 | 3,891 | ,120 | 2,986 | ,022 | ,083 | 11,619 | ,775 |
| | Límite-inferior | ,467 | 1,000 | ,467 | 2,986 | ,093 | ,083 | 2,986 | ,389 |
| Error(Tiempo) | Esfericidad asumida | 5,165 | 165 | ,031 | | | | | |
| | Greenhouse-Geisser | 5,165 | 113,577 | ,045 | | | | | |
| | Huynh-Feldt | 5,165 | 128,412 | ,040 | | | | | |
| | Límite-inferior | 5,165 | 33,000 | ,157 | | | | | |

a. Calculado con alfa = ,05

**Comparaciones por pares**

Medida:MEASURE_1

| (I)Tiempo | (J)Tiempo | Diferencia de medias (I-J) | Error típ. | Sig.[a] | Intervalo de confianza al 95 % para la diferencia[a] | |
|---|---|---|---|---|---|---|
| | | | | | Límite inferior | Límite superior |
| 1 | 2 | -,013 | ,036 | 1,000 | -,128 | ,102 |
| | 3 | ,023 | ,038 | 1,000 | -,098 | ,145 |
| | 4 | ,040 | ,035 | 1,000 | -,071 | ,151 |
| | 5 | ,134 | ,044 | ,069 | -,005 | ,273 |
| | 6 | ,020 | ,027 | 1,000 | -,064 | ,104 |
| 2 | 1 | ,013 | ,036 | 1,000 | -,102 | ,128 |
| | 3 | ,037 | ,046 | 1,000 | -,108 | ,181 |
| | 4 | ,054 | ,037 | 1,000 | -,062 | ,169 |
| | 5 | ,147 | ,057 | ,210 | -,032 | ,327 |
| | 6 | ,033 | ,040 | 1,000 | -,093 | ,160 |
| 3 | 1 | -,023 | ,038 | 1,000 | -,145 | ,098 |
| | 2 | -,037 | ,046 | 1,000 | -,181 | ,108 |
| | 4 | ,017 | ,049 | 1,000 | -,137 | ,170 |
| | 5 | ,111 | ,039 | ,125 | -,014 | ,235 |
| | 6 | -,003 | ,050 | 1,000 | -,160 | ,153 |
| 4 | 1 | -,040 | ,035 | 1,000 | -,151 | ,071 |
| | 2 | -,054 | ,037 | 1,000 | -,169 | ,062 |
| | 3 | -,017 | ,049 | 1,000 | -,170 | ,137 |
| | 5 | ,094 | ,048 | ,912 | -,059 | ,247 |
| | 6 | -,020 | ,038 | 1,000 | -,139 | ,099 |
| 5 | 1 | -,134 | ,044 | ,069 | -,273 | ,005 |
| | 2 | -,147 | ,057 | ,210 | -,327 | ,032 |
| | 3 | -,111 | ,039 | ,125 | -,235 | ,014 |
| | 4 | -,094 | ,048 | ,912 | -,247 | ,059 |
| | 6 | -,114 | ,051 | ,491 | -,276 | ,048 |
| 6 | 1 | -,020 | ,027 | 1,000 | -,104 | ,064 |
| | 2 | -,033 | ,040 | 1,000 | -,160 | ,093 |
| | 3 | ,003 | ,050 | 1,000 | -,153 | ,160 |
| | 4 | ,020 | ,038 | 1,000 | -,099 | ,139 |
| | 5 | ,114 | ,051 | ,491 | -,048 | ,276 |

Basadas en las medias marginales estimadas.

a. Ajuste para comparaciones múltiples: Bonferroni.

# MAXIMUM TAKEOFF VELOCITY

**Factores intra-sujetos**

Medida:MEASURE_1

| Tiempo | Variable dependiente |
|---|---|
| 1 | M_CMJ_Media_Pre_Vmáx |
| 2 | M_CMJ_Serie2_Vmáx |
| 3 | M_CMJ_Serie4_Vmáx |
| 4 | M_CMJ_Serie6_Vmáx |
| 5 | M_CMJ_Serie8_Vmáx |
| 6 | M_CMJ_Media_Post_Vmáx |

**Estadísticos descriptivos**

| | Media | Desviación típica | N |
|---|---|---|---|
| M_CMJ_Media_Pre_Vmáx | 2,8433 | ,14555 | 34 |
| M_CMJ_Serie2_Vmáx | 2,7436 | ,15690 | 34 |
| M_CMJ_Serie4_Vmáx | 2,7548 | ,16574 | 34 |
| M_CMJ_Serie6_Vmáx | 2,7254 | ,15473 | 34 |
| M_CMJ_Serie8_Vmáx | 2,7619 | ,15078 | 34 |
| M_CMJ_Media_Post_Vmáx | 2,8188 | ,17980 | 34 |

**Prueba de esfericidad de Mauchly[b]**

Medida:MEASURE_1

| Efecto intra-sujetos | W de Mauchly | Chi-cuadrado aprox. | gl | Sig. | Epsilon[a] | | |
|---|---|---|---|---|---|---|---|
| | | | | | Greenhouse-Geisser | Huynh-Feldt | Límite-inferior |
| Tiempo | ,457 | 24,381 | 14 | ,042 | ,767 | ,880 | ,200 |

Contrasta la hipótesis nula de que la matriz de covarianza error de las variables dependientes transformadas es proporcional a una matriz identidad.

a. Puede usarse para corregir los grados de libertad en las pruebas de significación promediadas. Las pruebas corregidas se muestran en la tabla Pruebas de los efectos inter-sujetos.

b. Diseño: Intersección

Diseño intra-sujetos: Tiempo

**Pruebas de efectos intra-sujetos.**

Medida:MEASURE_1

| Origen | | Suma de cuadrados tipo III | gl | Media cuadrática | F | Sig. | Eta al cuadrado parcial | Parámetro de no centralidad Parámetro | Potencia observada[a] |
|---|---|---|---|---|---|---|---|---|---|
| Tiempo | Esfericidad asumida | ,361 | 5 | ,072 | 15,163 | ,000 | ,315 | 75,816 | 1,000 |
| | Greenhouse-Geisser | ,361 | 3,833 | ,094 | 15,163 | ,000 | ,315 | 58,114 | 1,000 |
| | Huynh-Feldt | ,361 | 4,399 | ,082 | 15,163 | ,000 | ,315 | 66,702 | 1,000 |
| | Límite-inferior | ,361 | 1,000 | ,361 | 15,163 | ,000 | ,315 | 15,163 | ,965 |
| Error(Tiempo) | Esfericidad asumida | ,785 | 165 | ,005 | | | | | |
| | Greenhouse-Geisser | ,785 | 126,474 | ,006 | | | | | |
| | Huynh-Feldt | ,785 | 145,166 | ,005 | | | | | |
| | Límite-inferior | ,785 | 33,000 | ,024 | | | | | |

a. Calculado con alfa = ,05

**Comparaciones por pares**

Medida:MEASURE_1

| (I)Tiempo | (J)Tiempo | Diferencia de medias (I-J) | Error típ. | Sig.[a] | Intervalo de confianza al 95 % para la diferencia[a] | |
|---|---|---|---|---|---|---|
| | | | | | Límite inferior | Límite superior |
| 1 | 2 | ,100* | ,013 | ,000 | ,059 | ,141 |
| | 3 | ,088* | ,013 | ,000 | ,048 | ,129 |
| | 4 | ,118* | ,018 | ,000 | ,062 | ,174 |
| | 5 | ,081* | ,018 | ,001 | ,025 | ,138 |
| | 6 | ,024 | ,017 | 1,000 | -,031 | ,079 |
| 2 | 1 | -,100* | ,013 | ,000 | -,141 | -,059 |
| | 3 | -,011 | ,014 | 1,000 | -,054 | ,032 |
| | 4 | ,018 | ,019 | 1,000 | -,041 | ,077 |
| | 5 | -,018 | ,020 | 1,000 | -,083 | ,046 |
| | 6 | -,075* | ,018 | ,003 | -,133 | -,018 |
| 3 | 1 | -,088* | ,013 | ,000 | -,129 | -,048 |
| | 2 | ,011 | ,014 | 1,000 | -,032 | ,054 |
| | 4 | ,029 | ,015 | ,934 | -,019 | ,078 |
| | 5 | -,007 | ,015 | 1,000 | -,055 | ,041 |
| | 6 | -,064* | ,015 | ,002 | -,111 | -,017 |
| 4 | 1 | -,118* | ,018 | ,000 | -,174 | -,062 |
| | 2 | -,018 | ,019 | 1,000 | -,077 | ,041 |
| | 3 | -,029 | ,015 | ,934 | -,078 | ,019 |
| | 5 | -,037 | ,016 | ,488 | -,088 | ,015 |
| | 6 | -,093* | ,021 | ,001 | -,160 | -,027 |
| 5 | 1 | -,081* | ,018 | ,001 | -,138 | -,025 |
| | 2 | ,018 | ,020 | 1,000 | -,046 | ,083 |
| | 3 | ,007 | ,015 | 1,000 | -,041 | ,055 |
| | 4 | ,037 | ,016 | ,488 | -,015 | ,088 |
| | 6 | -,057* | ,016 | ,019 | -,108 | -,006 |
| 6 | 1 | -,024 | ,017 | 1,000 | -,079 | ,031 |
| | 2 | ,075* | ,018 | ,003 | ,018 | ,133 |
| | 3 | ,064* | ,015 | ,002 | ,017 | ,111 |
| | 4 | ,093* | ,021 | ,001 | ,027 | ,160 |
| | 5 | ,057* | ,016 | ,019 | ,006 | ,108 |

Basadas en las medias marginales estimadas.

*. La diferencia de medias es significativa al nivel ,05.

a. Ajuste para comparaciones múltiples: Bonferroni.
