# Supplementary material for: Muscular fatigue in response to different modalities of CrossFit sessions
Source: PLoS One. 2017 Jul 28;12(7):e0181855. doi: 10.1371/journal.pone.0181855 (PMC5533437; doi:10.1371/journal.pone.0181855)
Supplement: S6 File — Statistical analysis performed with mechanical variables in Metabolic WOD (PDF) [file pone.0181855.s006.pdf]

TOTAL DURATION JUMP

Factores intra-sujetos

Medida:MEASURE_1

Tiempo	Variable dependiente
1	M_CMJ_Med_Pre__Tiempo_TOTAL_repetición
2	M_CMJ_2__Tiempo_TOTAL_repetición
3	M_CMJ_4__Tiempo_TOTAL_repetición
4	M_CMJ_6__Tiempo_TOTAL_repetición
5	M_CMJ_8__Tiempo_TOTAL_repetición
6	M_CMJ_Med_Post__Tiempo_TOTAL_repetición

Estadísticos descriptivos

	Media	Desviación típica	N
M_CMJ_Med_Pre__Tiempo_TOTAL_repetición	,685137	,1478059	34
M_CMJ_2__Tiempo_TOTAL_repetición	,685118	,1432397	34
M_CMJ_4__Tiempo_TOTAL_repetición	,701176	,1565825	34
M_CMJ_6__Tiempo_TOTAL_repetición	,713118	,1713230	34
M_CMJ_8__Tiempo_TOTAL_repetición	,541706	,3398681	34
M_CMJ_Med_Post__Tiempo_TOTAL_repetición	,680902	,1997651	34

Contrastes multivariados[c]

Efecto		Valor	F	Gl de la hipótesis	Gl del error	Sig.	Eta al cuadrado parcial	Parámetro de no centralidad Parámetro	Potencia observada[b]
Tiempo	Traza de Pillai	,284	2,296[a]	5,000	29,000	,071	,284	11,479	,653
	Lambda de Wilks	,716	2,296[a]	5,000	29,000	,071	,284	11,479	,653
	Traza de Hotelling	,396	2,296[a]	5,000	29,000	,071	,284	11,479	,653
	Raíz mayor de Roy	,396	2,296[a]	5,000	29,000	,071	,284	11,479	,653

a. Estadístico exacto

b. Calculado con alfa = ,05

c. Diseño: Intersección

Diseño intra-sujetos: Tiempo

Prueba de esfericidad de Mauchly[b]

Medida:MEASURE_1

Efecto intra-sujetos	W de Mauchly	Chi-cuadrado aprox.	gl	Sig.	Epsilon[a]		
					Greenhouse-Geisser	Huynh-Feldt	Límite-inferior
Tiempo	,001	203,884	14	,000	,294	,305	,200

Contrasta la hipótesis nula de que la matriz de covarianza error de las variables dependientes transformadas es proporcional a una matriz identidad.

a. Puede usarse para corregir los grados de libertad en las pruebas de significación promediadas. Las pruebas corregidas se muestran en la tabla Pruebas de los efectos inter-sujetos.

b. Diseño: Intersección

Diseño intra-sujetos: Tiempo

Pruebas de efectos intra-sujetos.

Medida:MEASURE_1

Origen		Suma de cuadrados tipo III	gl	Media cuadrática	F	Sig.	Eta al cuadrado parcial	Parámetro de no centralidad Parámetro	Potencia observada[a]
Tiempo	Esfericidad asumida	,675	5	,135	5,246	,000	,137	26,229	,986
	Greenhouse-Geisser	,675	1,472	,458	5,246	,015	,137	7,720	,722
	Huynh-Feldt	,675	1,524	,443	5,246	,014	,137	7,992	,733
	Límite-inferior	,675	1,000	,675	5,246	,029	,137	5,246	,604
Error(Tiempo)	Esfericidad asumida	4,243	165	,026					
	Greenhouse-Geisser	4,243	48,564	,087					
	Huynh-Feldt	4,243	50,278	,084					
	Límite-inferior	4,243	33,000	,129					

a. Calculado con alfa = ,05

Comparaciones por pares

Medida:MEASURE_1

(I)Tiempo	(J)Tiempo	Diferencia de medias (I-J)	Error típ.	Sig.[a]	Intervalo de confianza al 95 % para la diferencia[a]	
					Límite inferior	Límite superior
1	2	1,961E-5	,009	1,000	-,027	,027
	3	-,016	,011	1,000	-,051	,019
	4	-,028	,013	,596	-,069	,013
	5	,143	,062	,409	-,053	,340
	6	,004	,026	1,000	-,080	,088
2	1	-1,961E-5	,009	1,000	-,027	,027
	3	-,016	,012	1,000	-,053	,020
	4	-,028	,012	,398	-,066	,010
	5	,143	,059	,321	-,044	,331
	6	,004	,025	1,000	-,076	,085
3	1	,016	,011	1,000	-,019	,051
	2	,016	,012	1,000	-,020	,053
	4	-,012	,014	1,000	-,057	,034
	5	,159	,061	,197	-,033	,352
	6	,020	,026	1,000	-,061	,101
4	1	,028	,013	,596	-,013	,069
	2	,028	,012	,398	-,010	,066
	3	,012	,014	1,000	-,034	,057
	5	,171	,063	,148	-,027	,369
	6	,032	,027	1,000	-,052	,116
5	1	-,143	,062	,409	-,340	,053
	2	-,143	,059	,321	-,331	,044
	3	-,159	,061	,197	-,352	,033
	4	-,171	,063	,148	-,369	,027
	6	-,139	,064	,566	-,343	,064
6	1	-,004	,026	1,000	-,088	,080
	2	-,004	,025	1,000	-,085	,076
	3	-,020	,026	1,000	-,101	,061
	4	-,032	,027	1,000	-,116	,052
	5	,139	,064	,566	-,064	,343

Basadas en las medias marginales estimadas.

a. Ajuste para comparaciones múltiples: Bonferroni.

ECCENTRIC PHASE

Factores intra-sujetos

Medida:MEASURE_1

Tiempo	Variable dependiente
1	M_CMJ_Med_Pre__Tiempo_EXCENTRICO_repetición
2	M_CMJ_2__Tiempo_EXCENTRICO_repetición
3	M_CMJ_4__Tiempo_EXCENTRICO_repetición
4	M_CMJ_6__Tiempo_EXCENTRICO_repetición
5	M_CMJ_8__Tiempo_EXCENTRICO_repetición
6	M_CMJ_Med_Post__Tiempo_EXCENTRICO_repetición

Estadísticos descriptivos

	Media	Desviación típica	N
M_CMJ_Med_Pre__Tiempo_EXCENTRICO_repetición	,482323	,0742365	32
M_CMJ_2__Tiempo_EXCENTRICO_repetición	,455563	,0725547	32
M_CMJ_4__Tiempo_EXCENTRICO_repetición	,479625	,0918434	32
M_CMJ_6__Tiempo_EXCENTRICO_repetición	,488313	,0978638	32
M_CMJ_8__Tiempo_EXCENTRICO_repetición	,371188	,2278827	32
M_CMJ_Med_Post__Tiempo_EXCENTRICO_repetición	,499729	,0961763	32

Contrastes multivariados[c]

Efecto		Valor	F	Gl de la hipótesis	Gl del error	Sig.	Eta al cuadrado parcial	Parámetro de no centralidad Parámetro	Potencia observada[b]
Tiempo	Traza de Pillai	,419	3,900[a]	5,000	27,000	,009	,419	19,500	,889
	Lambda de Wilks	,581	3,900[a]	5,000	27,000	,009	,419	19,500	,889
	Traza de Hotelling	,722	3,900[a]	5,000	27,000	,009	,419	19,500	,889
	Raíz mayor de Roy	,722	3,900[a]	5,000	27,000	,009	,419	19,500	,889

a. Estadístico exacto

b. Calculado con alfa = ,05

c. Diseño: Intersección

Diseño intra-sujetos: Tiempo

Prueba de esfericidad de Mauchly[b]

Medida:MEASURE_1

Efecto intra-sujetos	W de Mauchly	Chi-cuadrado aprox.	gl	Sig.	Epsilon[a]		
					Greenhouse-Geisser	Huynh-Feldt	Límite-inferior
Tiempo	,006	146,907	14	,000	,298	,310	,200

Contrasta la hipótesis nula de que la matriz de covarianza error de las variables dependientes transformadas es proporcional a una matriz identidad.

a. Puede usarse para corregir los grados de libertad en las pruebas de significación promediadas. Las pruebas corregidas se muestran en la tabla Pruebas de los efectos inter-sujetos.

b. Diseño: Intersección

Diseño intra-sujetos: Tiempo

Pruebas de efectos intra-sujetos.

Medida:MEASURE_1

Origen		Suma de cuadrados tipo III	gl	Media cuadrática	F	Sig.	Eta al cuadrado parcial	Parámetro de no centralidad Parámetro	Potencia observada[a]
Tiempo	Esfericidad asumida	,356	5	,071	5,282	,000	,146	26,412	,986
	Greenhouse-Geisser	,356	1,492	,239	5,282	,015	,146	7,881	,728
	Huynh-Feldt	,356	1,550	,230	5,282	,014	,146	8,189	,740
	Límite-inferior	,356	1,000	,356	5,282	,028	,146	5,282	,605
Error(Tiempo)	Esfericidad asumida	2,089	155	,013					
	Greenhouse-Geisser	2,089	46,251	,045					
	Huynh-Feldt	2,089	48,056	,043					
	Límite-inferior	2,089	31,000	,067					

a. Calculado con alfa = ,05

Comparaciones por pares

Medida:MEASURE_1

(I)Tiempo	(J)Tiempo	Diferencia de medias (I-J)	Error típ.	Sig.[a]	Intervalo de confianza al 95 % para la diferencia[a]	
					Límite inferior	Límite superior
1	2	,027	,009	,105	-,003	,056
	3	,003	,013	1,000	-,039	,044
	4	-,006	,014	1,000	-,051	,039
	5	,111	,047	,366	-,038	,260
	6	-,017	,017	1,000	-,071	,036
2	1	-,027	,009	,105	-,056	,003
	3	-,024	,011	,529	-,059	,011
	4	-,033	,013	,204	-,073	,007
	5	,084	,044	,997	-,057	,225
	6	-,044	,015	,104	-,093	,004
3	1	-,003	,013	1,000	-,044	,039
	2	,024	,011	,529	-,011	,059
	4	-,009	,016	1,000	-,058	,041
	5	,108	,048	,448	-,043	,260
	6	-,020	,018	1,000	-,077	,037
4	1	,006	,014	1,000	-,039	,051
	2	,033	,013	,204	-,007	,073
	3	,009	,016	1,000	-,041	,058
	5	,117	,048	,293	-,034	,268
	6	-,011	,017	1,000	-,065	,042
5	1	-,111	,047	,366	-,260	,038
	2	-,084	,044	,997	-,225	,057
	3	-,108	,048	,448	-,260	,043
	4	-,117	,048	,293	-,268	,034
	6	-,129	,043	,079	-,265	,008
6	1	,017	,017	1,000	-,036	,071
	2	,044	,015	,104	-,004	,093
	3	,020	,018	1,000	-,037	,077
	4	,011	,017	1,000	-,042	,065
	5	,129	,043	,079	-,008	,265

Basadas en las medias marginales estimadas.

a. Ajuste para comparaciones múltiples: Bonferroni.

ISOMETRIC PHASE

Factores intra-sujetos

Medida:MEASURE_1

Tiempo	Variable dependiente
1	M_CMJ_Med_Pre__Tiempo_ISOMÉTRICO_repetición
2	M_CMJ_2__Tiempo_ISOMÉTRICO_repetición
3	M_CMJ_4__Tiempo_ISOMÉTRICO_repetición
4	M_CMJ_6__Tiempo_ISOMÉTRICO_repetición
5	M_CMJ_8__Tiempo_ISOMÉTRICO_repetición
6	M_CMJ_Med_Post__Tiempo_ISOMÉTRICO_repetición

Estadísticos descriptivos

	Media	Desviación típica	N
M_CMJ_Med_Pre__Tiempo_ISOMÉTRICO_repetición	,008778	,0030498	24
M_CMJ_2__Tiempo_ISOMÉTRICO_repetición	,008333	,0057383	24
M_CMJ_4__Tiempo_ISOMÉTRICO_repetición	,008417	,0043730	24
M_CMJ_6__Tiempo_ISOMÉTRICO_repetición	,010583	,0063651	24
M_CMJ_8__Tiempo_ISOMÉTRICO_repetición	,014083	,0176584	24
M_CMJ_Med_Post__Tiempo_ISOMÉTRICO_repetición	,009222	,0022558	24

Contrastes multivariados[c]

Efecto		Valor	F	Gl de la hipótesis	Gl del error	Sig.	Eta al cuadrado parcial	Parámetro de no centralidad Parámetro	Potencia observada[b]
Tiempo	Traza de Pillai	,250	1,264[a]	5,000	19,000	,320	,250	6,319	,349
	Lambda de Wilks	,750	1,264[a]	5,000	19,000	,320	,250	6,319	,349
	Traza de Hotelling	,333	1,264[a]	5,000	19,000	,320	,250	6,319	,349
	Raíz mayor de Roy	,333	1,264[a]	5,000	19,000	,320	,250	6,319	,349

a. Estadístico exacto

b. Calculado con alfa = ,05

c. Diseño: Intersección

Diseño intra-sujetos: Tiempo

Prueba de esfericidad de Mauchly[b]

Medida:MEASURE_1

Efecto intra-sujetos	W de Mauchly	Chi-cuadrado aprox.	gl	Sig.	Epsilon[a]		
					Greenhouse-Geisser	Huynh-Feldt	Límite-inferior
Tiempo	,002	127,633	14	,000	,306	,324	,200

Contrasta la hipótesis nula de que la matriz de covarianza error de las variables dependientes transformadas es proporcional a una matriz identidad.

a. Puede usarse para corregir los grados de libertad en las pruebas de significación promediadas. Las pruebas corregidas se muestran en la tabla Pruebas de los efectos inter-sujetos.

b. Diseño: Intersección

Diseño intra-sujetos: Tiempo

Pruebas de efectos intra-sujetos.

Medida:MEASURE_1

Origen		Suma de cuadrados tipo III	gl	Media cuadrática	F	Sig.	Eta al cuadrado parcial	Parámetro de no centralidad Parámetro	Potencia observada[a]
Tiempo	Esfericidad asumida	,001	5	,000	1,829	,113	,074	9,147	,608
	Greenhouse-Geisser	,001	1,532	,000	1,829	,182	,074	2,803	,314
	Huynh-Feldt	,001	1,620	,000	1,829	,180	,074	2,963	,323
	Límite-inferior	,001	1,000	,001	1,829	,189	,074	1,829	,254
Error(Tiempo)	Esfericidad asumida	,007	115	6,387E-5					
	Greenhouse-Geisser	,007	35,238	,000					
	Huynh-Feldt	,007	37,252	,000					
	Límite-inferior	,007	23,000	,000					

a. Calculado con alfa = ,05

Comparaciones por pares

Medida:MEASURE_1

(I)Tiempo	(J)Tiempo	Diferencia de medias (I-J)	Error típ.	Sig.[a]	Intervalo de confianza al 95 % para la diferencia[a]	
					Límite inferior	Límite superior
1	2	,000	,001	1,000	-,003	,004
	3	,000	,001	1,000	-,003	,004
	4	-,002	,001	1,000	-,006	,002
	5	-,005	,004	1,000	-,017	,006
	6	,000	,001	1,000	-,002	,002
2	1	,000	,001	1,000	-,004	,003
	3	-8,333E-5	,001	1,000	-,003	,003
	4	-,002	,002	1,000	-,008	,003
	5	-,006	,003	1,000	-,016	,005
	6	-,001	,001	1,000	-,005	,003
3	1	,000	,001	1,000	-,004	,003
	2	8,333E-5	,001	1,000	-,003	,003
	4	-,002	,002	1,000	-,008	,003
	5	-,006	,004	1,000	-,018	,007
	6	-,001	,001	1,000	-,004	,002
4	1	,002	,001	1,000	-,002	,006
	2	,002	,002	1,000	-,003	,008
	3	,002	,002	1,000	-,003	,008
	5	-,004	,004	1,000	-,015	,008
	6	,001	,001	1,000	-,003	,006
5	1	,005	,004	1,000	-,006	,017
	2	,006	,003	1,000	-,005	,016
	3	,006	,004	1,000	-,007	,018
	4	,004	,004	1,000	-,008	,015
	6	,005	,004	1,000	-,007	,017
6	1	,000	,001	1,000	-,002	,002
	2	,001	,001	1,000	-,003	,005
	3	,001	,001	1,000	-,002	,004
	4	-,001	,001	1,000	-,006	,003
	5	-,005	,004	1,000	-,017	,007

Basadas en las medias marginales estimadas.

a. Ajuste para comparaciones múltiples: Bonferroni.

CONCENTRIC PHASE

Factores intra-sujetos

Medida:MEASURE_1

Tiempo	Variable dependiente
1	M_CMJ_Med_Pre__Tiempo_CONCÉNTRICO_repetición
2	M_CMJ_2__Tiempo_CONCÉNTRICO_repetición
3	M_CMJ_4__Tiempo_CONCÉNTRICO_repetición
4	M_CMJ_6__Tiempo_CONCÉNTRICO_repetición
5	M_CMJ_8__Tiempo_CONCÉNTRICO_repetición
6	M_CMJ_Med_Post__Tiempo_CONCÉNTRICO_repetición

Estadísticos descriptivos

	Media	Desviación típica	N
M_CMJ_Med_Pre__Tiempo_CONCÉNTRICO_repetición	,212653	,0503987	24
M_CMJ_2__Tiempo_CONCÉNTRICO_repetición	,240917	,0442364	24
M_CMJ_4__Tiempo_CONCÉNTRICO_repetición	,238417	,0461377	24
M_CMJ_6__Tiempo_CONCÉNTRICO_repetición	,237000	,0524230	24
M_CMJ_8__Tiempo_CONCÉNTRICO_repetición	,227000	,0654934	24
M_CMJ_Med_Post__Tiempo_CONCÉNTRICO_repetición	,212889	,0571245	24

Contrastes multivariados[c]

Efecto		Valor	F	Gl de la hipótesis	Gl del error	Sig.	Eta al cuadrado parcial	Parámetro de no centralidad Parámetro	Potencia observada[b]
Tiempo	Traza de Pillai	,634	6,591[a]	5,000	19,000	,001	,634	32,956	,983
	Lambda de Wilks	,366	6,591[a]	5,000	19,000	,001	,634	32,956	,983
	Traza de Hotelling	1,735	6,591[a]	5,000	19,000	,001	,634	32,956	,983
	Raíz mayor de Roy	1,735	6,591[a]	5,000	19,000	,001	,634	32,956	,983

a. Estadístico exacto

b. Calculado con alfa = ,05

c. Diseño: Intersección

Diseño intra-sujetos: Tiempo

Prueba de esfericidad de Mauchly[b]

Medida:MEASURE_1

Efecto intra-sujetos	W de Mauchly	Chi-cuadrado aprox.	gl	Sig.	Epsilon[a]		
					Greenhouse-Geisser	Huynh-Feldt	Límite-inferior
Tiempo	,183	35,789	14	,001	,592	,690	,200

Contrasta la hipótesis nula de que la matriz de covarianza error de las variables dependientes transformadas es proporcional a una matriz identidad.

a. Puede usarse para corregir los grados de libertad en las pruebas de significación promediadas. Las pruebas corregidas se muestran en la tabla Pruebas de los efectos inter-sujetos.

b. Diseño: Intersección

Diseño intra-sujetos: Tiempo

Pruebas de efectos intra-sujetos.

Medida:MEASURE_1

Origen		Suma de cuadrados tipo III	gl	Media cuadrática	F	Sig.	Eta al cuadrado parcial	Parámetro de no centralidad Parámetro	Potencia observada[a]
Tiempo	Esfericidad asumida	,020	5	,004	3,981	,002	,148	19,906	,940
	Greenhouse-Geisser	,020	2,962	,007	3,981	,012	,148	11,794	,811
	Huynh-Feldt	,020	3,449	,006	3,981	,008	,148	13,729	,855
	Límite-inferior	,020	1,000	,020	3,981	,058	,148	3,981	,481
Error(Tiempo)	Esfericidad asumida	,114	115	,001					
	Greenhouse-Geisser	,114	68,137	,002					
	Huynh-Feldt	,114	79,316	,001					
	Límite-inferior	,114	23,000	,005					

a. Calculado con alfa = ,05

Comparaciones por pares

Medida:MEASURE_1

(I)Tiempo	(J)Tiempo	Diferencia de medias (I-J)	Error típ.	Sig.[a]	Intervalo de confianza al 95 % para la diferencia[a]	
					Límite inferior	Límite superior
1	2	-,028*	,007	,014	-,053	-,004
	3	-,026*	,007	,023	-,049	-,002
	4	-,024	,009	,174	-,053	,005
	5	-,014	,013	1,000	-,057	,029
	6	,000	,010	1,000	-,032	,032
2	1	,028*	,007	,014	,004	,053
	3	,003	,006	1,000	-,016	,021
	4	,004	,008	1,000	-,021	,029
	5	,014	,012	1,000	-,026	,053
	6	,028*	,007	,010	,005	,051
3	1	,026*	,007	,023	,002	,049
	2	-,003	,006	1,000	-,021	,016
	4	,001	,005	1,000	-,016	,019
	5	,011	,012	1,000	-,028	,050
	6	,026*	,007	,015	,003	,048
4	1	,024	,009	,174	-,005	,053
	2	-,004	,008	1,000	-,029	,021
	3	-,001	,005	1,000	-,019	,016
	5	,010	,011	1,000	-,026	,046
	6	,024	,008	,070	-,001	,049
5	1	,014	,013	1,000	-,029	,057
	2	-,014	,012	1,000	-,053	,026
	3	-,011	,012	1,000	-,050	,028
	4	-,010	,011	1,000	-,046	,026
	6	,014	,010	1,000	-,019	,047
6	1	,000	,010	1,000	-,032	,032
	2	-,028*	,007	,010	-,051	-,005
	3	-,026*	,007	,015	-,048	-,003
	4	-,024	,008	,070	-,049	,001
	5	-,014	,010	1,000	-,047	,019

Basadas en las medias marginales estimadas.

*. La diferencia de medias es significativa al nivel ,05.

a. Ajuste para comparaciones múltiples: Bonferroni.
