# Supplementary material for: Muscular fatigue in response to different modalities of CrossFit sessions
Source: PLoS One. 2017 Jul 28;12(7):e0181855. doi: 10.1371/journal.pone.0181855 (PMC5533437; doi:10.1371/journal.pone.0181855)
Supplement: S7 File — Statistical analysis performed with mechanical variables in Weight WOD. (PDF) [file pone.0181855.s007.pdf]

JUMP HEIGHT

Factores intra-sujetos

Medida:MEASURE_1

Tiempo	Variable dependiente
1	W_CMJ_Med_Pre_AlturaVuelo
2	W_CMJ_min2ymedio_AlturaVuelo
3	W_CMJ_Med_Post_AlturaVuelo

Estadísticos descriptivos

	Media	Desviación típica	N
W_CMJ_Med_Pre_AlturaVuelo	36,5954	4,04905	34
W_CMJ_min2ymedio_AlturaVuelo	32,8483	5,26415	34
W_CMJ_Med_Post_AlturaVuelo	33,9017	4,85898	34

Contrastes multivariados[c]

Efecto		Valor	F	Gl de la hipótesis	Gl del error	Sig.	Eta al cuadrado parcial	Parámetro de no centralidad Parámetro	Potencia observada[b]
Tiempo	Traza de Pillai	,574	21,547[a]	2,000	32,000	,000	,574	43,094	1,000
	Lambda de Wilks	,426	21,547[a]	2,000	32,000	,000	,574	43,094	1,000
	Traza de Hotelling	1,347	21,547[a]	2,000	32,000	,000	,574	43,094	1,000
	Raíz mayor de Roy	1,347	21,547[a]	2,000	32,000	,000	,574	43,094	1,000

a. Estadístico exacto

b. Calculado con alfa = ,05

c. Diseño: Intersección

Diseño intra-sujetos: Tiempo

Prueba de esfericidad de Mauchly[b]

Medida:MEASURE_1

Efecto intra-sujetos	W de Mauchly	Chi-cuadrado aprox.	gl	Sig.	Epsilon[a]		
					Greenhouse-Geisser	Huynh-Feldt	Límite-inferior
Tiempo	,787	7,653	2	,022	,825	,862	,500

Contrasta la hipótesis nula de que la matriz de covarianza error de las variables dependientes transformadas es proporcional a una matriz identidad.

a. Puede usarse para corregir los grados de libertad en las pruebas de significación promediadas. Las pruebas corregidas se muestran en la tabla Pruebas de los efectos inter-sujetos.

b. Diseño: Intersección

Diseño intra-sujetos: Tiempo

Pruebas de efectos intra-sujetos.

Medida:MEASURE_1

Origen		Suma de cuadrados tipo III	gl	Media cuadrática	F	Sig.	Eta al cuadrado parcial	Parámetro de no centralidad Parámetro	Potencia observada[a]
Tiempo	Esfericidad asumida	253,937	2	126,968	27,413	,000	,454	54,826	1,000
	Greenhouse-Geisser	253,937	1,649	153,976	27,413	,000	,454	45,209	1,000
	Huynh-Feldt	253,937	1,725	147,230	27,413	,000	,454	47,281	1,000
	Límite-inferior	253,937	1,000	253,937	27,413	,000	,454	27,413	,999
Error(Tiempo)	Esfericidad asumida	305,691	66	4,632					
	Greenhouse-Geisser	305,691	54,423	5,617					
	Huynh-Feldt	305,691	56,917	5,371					
	Límite-inferior	305,691	33,000	9,263					

a. Calculado con alfa = ,05

Comparaciones por pares

Medida:MEASURE_1

(I)Tiempo	(J)Tiempo	Diferencia de medias (I-J)	Error típ.	Sig.[a]	Intervalo de confianza al 95 % para la diferencia[a]	
					Límite inferior	Límite superior
1	2	3,747*	,629	,000	2,159	5,335
	3	2,694*	,438	,000	1,589	3,798
2	1	-3,747*	,629	,000	-5,335	-2,159
	3	-1,053	,479	,105	-2,261	,155
3	1	-2,694*	,438	,000	-3,798	-1,589
	2	1,053	,479	,105	-,155	2,261

Basadas en las medias marginales estimadas.

*. La diferencia de medias es significativa al nivel ,05.

a. Ajuste para comparaciones múltiples: Bonferroni.

PEAK POWER RELATIVE

Factores intra-sujetos

Medida:MEASURE_1

Tiempo	Variable dependiente
1	W_CMJ_Med_Pre_Pmax_rel
2	W_CMJ_min2ymedio_Pmax_rel
3	W_CMJ_Med_Post_Pmax_rel

Estadísticos descriptivos

	Media	Desviación típica	N
W_CMJ_Med_Pre_Pmax_rel	54,5875	5,36670	34
W_CMJ_min2ymedio_Pmax_rel	51,2062	5,78566	34
W_CMJ_Med_Post_Pmax_rel	53,0321	5,77153	34

Contrastes multivariados[c]

Efecto		Valor	F	Gl de la hipótesis	Gl del error	Sig.	Eta al cuadrado parcial	Parámetro de no centralidad Parámetro	Potencia observada[b]
Tiempo	Traza de Pillai	,582	22,255[a]	2,000	32,000	,000	,582	44,510	1,000
	Lambda de Wilks	,418	22,255[a]	2,000	32,000	,000	,582	44,510	1,000
	Traza de Hotelling	1,391	22,255[a]	2,000	32,000	,000	,582	44,510	1,000
	Raíz mayor de Roy	1,391	22,255[a]	2,000	32,000	,000	,582	44,510	1,000

a. Estadístico exacto

b. Calculado con alfa = ,05

c. Diseño: Intersección

Diseño intra-sujetos: Tiempo

Prueba de esfericidad de Mauchly[b]

Medida:MEASURE_1

Efecto intra-sujetos	W de Mauchly	Chi-cuadrado aprox.	gl	Sig.	Epsilon[a]		
					Greenhouse-Geisser	Huynh-Feldt	Límite-inferior
Tiempo	,966	1,092	2	,579	,968	1,000	,500

Contrasta la hipótesis nula de que la matriz de covarianza error de las variables dependientes transformadas es proporcional a una matriz identidad.

a. Puede usarse para corregir los grados de libertad en las pruebas de significación promediadas. Las pruebas corregidas se muestran en la tabla Pruebas de los efectos inter-sujetos.

b. Diseño: Intersección

Diseño intra-sujetos: Tiempo

Pruebas de efectos intra-sujetos.

Medida:MEASURE_1

Origen		Suma de cuadrados tipo III	gl	Media cuadrática	F	Sig.	Eta al cuadrado parcial	Parámetro de no centralidad Parámetro	Potencia observada[a]
Tiempo	Esfericidad asumida	194,777	2	97,388	24,558	,000	,427	49,116	1,000
	Greenhouse-Geisser	194,777	1,935	100,656	24,558	,000	,427	47,522	1,000
	Huynh-Feldt	194,777	2,000	97,388	24,558	,000	,427	49,116	1,000
	Límite-inferior	194,777	1,000	194,777	24,558	,000	,427	24,558	,998
Error(Tiempo)	Esfericidad asumida	261,733	66	3,966					
	Greenhouse-Geisser	261,733	63,858	4,099					
	Huynh-Feldt	261,733	66,000	3,966					
	Límite-inferior	261,733	33,000	7,931					

a. Calculado con alfa = ,05

Comparaciones por pares

Medida:MEASURE_1

(I)Tiempo	(J)Tiempo	Diferencia de medias (I-J)	Error típ.	Sig.[a]	Intervalo de confianza al 95 % para la diferencia[a]	
					Límite inferior	Límite superior
1	2	3,381*	,509	,000	2,097	4,665
	3	1,555*	,500	,011	,295	2,816
2	1	-3,381*	,509	,000	-4,665	-2,097
	3	-1,826*	,437	,001	-2,928	-,724
3	1	-1,555*	,500	,011	-2,816	-,295
	2	1,826*	,437	,001	,724	2,928

Basadas en las medias marginales estimadas.

*. La diferencia de medias es significativa al nivel ,05.

a. Ajuste para comparaciones múltiples: Bonferroni.

PEAK POWER TOTAL

Factores intra-sujetos

Medida:MEASURE_1

Tiempo	Variable dependiente
1	W_CMJ_Med_Pre_Pmax_total
2	W_CMJ_min2ymedio_Pmax_total
3	W_CMJ_Med_Post_Pmax_total

Estadísticos descriptivos

	Media	Desviación típica	N
W_CMJ_Med_Pre_Pmax_total	4187,0683	535,76404	34
W_CMJ_min2ymedio_Pmax_total	3924,3441	527,88687	34
W_CMJ_Med_Post_Pmax_total	4071,4620	519,09316	34

Contrastes multivariados[c]

Efecto		Valor	F	Gl de la hipótesis	Gl del error	Sig.	Eta al cuadrado parcial	Parámetro de no centralidad Parámetro	Potencia observada[b]
Tiempo	Traza de Pillai	,571	21,269[a]	2,000	32,000	,000	,571	42,539	1,000
	Lambda de Wilks	,429	21,269[a]	2,000	32,000	,000	,571	42,539	1,000
	Traza de Hotelling	1,329	21,269[a]	2,000	32,000	,000	,571	42,539	1,000
	Raíz mayor de Roy	1,329	21,269[a]	2,000	32,000	,000	,571	42,539	1,000

a. Estadístico exacto

b. Calculado con alfa = ,05

c. Diseño: Intersección

Diseño intra-sujetos: Tiempo

Prueba de esfericidad de Mauchly[b]

Medida:MEASURE_1

Efecto intra-sujetos	W de Mauchly	Chi-cuadrado aprox.	gl	Sig.	Epsilon[a]		
					Greenhouse-Geisser	Huynh-Feldt	Límite-inferior
Tiempo	,962	1,223	2	,542	,964	1,000	,500

Contrasta la hipótesis nula de que la matriz de covarianza error de las variables dependientes transformadas es proporcional a una matriz identidad.

a. Puede usarse para corregir los grados de libertad en las pruebas de significación promediadas. Las pruebas corregidas se muestran en la tabla Pruebas de los efectos inter-sujetos.

b. Diseño: Intersección

Diseño intra-sujetos: Tiempo

Pruebas de efectos intra-sujetos.

Medida:MEASURE_1

Origen		Suma de cuadrados tipo III	gl	Media cuadrática	F	Sig.	Eta al cuadrado parcial	Parámetro de no centralidad Parámetro	Potencia observada[a]
Tiempo	Esfericidad asumida	1179034,671	2	589517,335	20,305	,000	,381	40,610	1,000
	Greenhouse-Geisser	1179034,671	1,928	611625,575	20,305	,000	,381	39,142	1,000
	Huynh-Feldt	1179034,671	2,000	589517,335	20,305	,000	,381	40,610	1,000
	Límite-inferior	1179034,671	1,000	1179034,671	20,305	,000	,381	20,305	,992
Error(Tiempo)	Esfericidad asumida	1916170,445	66	29032,886					
	Greenhouse-Geisser	1916170,445	63,614	30121,685					
	Huynh-Feldt	1916170,445	66,000	29032,886					
	Límite-inferior	1916170,445	33,000	58065,771					

a. Calculado con alfa = ,05

Comparaciones por pares

Medida:MEASURE_1

(I)Tiempo	(J)Tiempo	Diferencia de medias (I-J)	Error típ.	Sig.[a]	Intervalo de confianza al 95 % para la diferencia[a]	
					Límite inferior	Límite superior
1	2	262,724*	41,052	,000	159,182	366,266
	3	115,606*	44,776	,043	2,673	228,540
2	1	-262,724*	41,052	,000	-366,266	-159,182
	3	-147,118*	37,860	,001	-242,608	-51,628
3	1	-115,606*	44,776	,043	-228,540	-2,673
	2	147,118*	37,860	,001	51,628	242,608

Basadas en las medias marginales estimadas.

*. La diferencia de medias es significativa al nivel ,05.

a. Ajuste para comparaciones múltiples: Bonferroni.

AVERAGE POWER RELATIVE

Factores intra-sujetos

Medida:MEASURE_1

Tiempo	Variable dependiente
1	W_CMJ_Med_Pre_Pmed_rel
2	W_CMJ_min2ymedio_Pmed_rel
3	W_CMJ_Med_Post_Pmed_rel

Estadísticos descriptivos

	Media	Desviación típica	N
W_CMJ_Med_Pre_Pmed_rel	30,9504	3,38239	34
W_CMJ_min2ymedio_Pmed_rel	27,9324	4,06125	34
W_CMJ_Med_Post_Pmed_rel	28,6650	3,88942	34

Contrastes multivariados[c]

Efecto		Valor	F	Gl de la hipótesis	Gl del error	Sig.	Eta al cuadrado parcial	Parámetro de no centralidad Parámetro	Potencia observada[b]
Tiempo	Traza de Pillai	,625	26,701[a]	2,000	32,000	,000	,625	53,402	1,000
	Lambda de Wilks	,375	26,701[a]	2,000	32,000	,000	,625	53,402	1,000
	Traza de Hotelling	1,669	26,701[a]	2,000	32,000	,000	,625	53,402	1,000
	Raíz mayor de Roy	1,669	26,701[a]	2,000	32,000	,000	,625	53,402	1,000

a. Estadístico exacto

b. Calculado con alfa = ,05

c. Diseño: Intersección

Diseño intra-sujetos: Tiempo

Prueba de esfericidad de Mauchly[b]

Medida:MEASURE_1

Efecto intra-sujetos	W de Mauchly	Chi-cuadrado aprox.	gl	Sig.	Epsilon[a]		
					Greenhouse-Geisser	Huynh-Feldt	Límite-inferior
Tiempo	,827	6,092	2	,048	,852	,894	,500

Contrasta la hipótesis nula de que la matriz de covarianza error de las variables dependientes transformadas es proporcional a una matriz identidad.

a. Puede usarse para corregir los grados de libertad en las pruebas de significación promediadas. Las pruebas corregidas se muestran en la tabla Pruebas de los efectos inter-sujetos.

b. Diseño: Intersección

Diseño intra-sujetos: Tiempo

Pruebas de efectos intra-sujetos.

Medida:MEASURE_1

Origen		Suma de cuadrados tipo III	gl	Media cuadrática	F	Sig.	Eta al cuadrado parcial	Parámetro de no centralidad Parámetro	Potencia observada[a]
Tiempo	Esfericidad asumida	168,507	2	84,254	38,995	,000	,542	77,991	1,000
	Greenhouse-Geisser	168,507	1,705	98,859	38,995	,000	,542	66,468	1,000
	Huynh-Feldt	168,507	1,788	94,248	38,995	,000	,542	69,720	1,000
	Límite-inferior	168,507	1,000	168,507	38,995	,000	,542	38,995	1,000
Error(Tiempo)	Esfericidad asumida	142,600	66	2,161					
	Greenhouse-Geisser	142,600	56,249	2,535					
	Huynh-Feldt	142,600	59,001	2,417					
	Límite-inferior	142,600	33,000	4,321					

a. Calculado con alfa = ,05

Comparaciones por pares

Medida:MEASURE_1

(I)Tiempo	(J)Tiempo	Diferencia de medias (I-J)	Error típ.	Sig.[a]	Intervalo de confianza al 95 % para la diferencia[a]	
					Límite inferior	Límite superior
1	2	3,018*	,413	,000	1,975	4,061
	3	2,285*	,362	,000	1,373	3,198
2	1	-3,018*	,413	,000	-4,061	-1,975
	3	-,733*	,282	,042	-1,444	-,022
3	1	-2,285*	,362	,000	-3,198	-1,373
	2	,733*	,282	,042	,022	1,444

Basadas en las medias marginales estimadas.

*. La diferencia de medias es significativa al nivel ,05.

a. Ajuste para comparaciones múltiples: Bonferroni.

AVERAGE POWER TOTAL

Factores intra-sujetos

Medida:MEASURE_1

Tiempo	Variable dependiente
1	W_CMJ_Med_Pre_Pmed_total
2	W_CMJ_min2ymedio_Pmed_total
3	W_CMJ_Med_Post_Pmed_total

Estadísticos descriptivos

	Media	Desviación típica	N
W_CMJ_Med_Pre_Pmed_total	2371,2707	309,48808	34
W_CMJ_min2ymedio_Pmed_total	2140,9505	348,23762	34
W_CMJ_Med_Post_Pmed_total	2198,0279	319,38209	34

Contrastes multivariados[c]

Efecto		Valor	F	Gl de la hipótesis	Gl del error	Sig.	Eta al cuadrado parcial	Parámetro de no centralidad Parámetro	Potencia observada[b]
Tiempo	Traza de Pillai	,614	25,403[a]	2,000	32,000	,000	,614	50,806	1,000
	Lambda de Wilks	,386	25,403[a]	2,000	32,000	,000	,614	50,806	1,000
	Traza de Hotelling	1,588	25,403[a]	2,000	32,000	,000	,614	50,806	1,000
	Raíz mayor de Roy	1,588	25,403[a]	2,000	32,000	,000	,614	50,806	1,000

a. Estadístico exacto

b. Calculado con alfa = ,05

c. Diseño: Intersección

Diseño intra-sujetos: Tiempo

Prueba de esfericidad de Mauchly[b]

Medida:MEASURE_1

Efecto intra-sujetos	W de Mauchly	Chi-cuadrado aprox.	gl	Sig.	Epsilon[a]		
					Greenhouse-Geisser	Huynh-Feldt	Límite-inferior
Tiempo	,869	4,500	2	,105	,884	,930	,500

Contrasta la hipótesis nula de que la matriz de covarianza error de las variables dependientes transformadas es proporcional a una matriz identidad.

a. Puede usarse para corregir los grados de libertad en las pruebas de significación promediadas. Las pruebas corregidas se muestran en la tabla Pruebas de los efectos inter-sujetos.

b. Diseño: Intersección

Diseño intra-sujetos: Tiempo

Pruebas de efectos intra-sujetos.

Medida:MEASURE_1

Origen		Suma de cuadrados tipo III	gl	Media cuadrática	F	Sig.	Eta al cuadrado parcial	Parámetro de no centralidad Parámetro	Potencia observada[a]
Tiempo	Esfericidad asumida	978273,293	2	489136,646	34,211	,000	,509	68,421	1,000
	Greenhouse-Geisser	978273,293	1,768	553309,070	34,211	,000	,509	60,486	1,000
	Huynh-Feldt	978273,293	1,861	525754,623	34,211	,000	,509	63,656	1,000
	Límite-inferior	978273,293	1,000	978273,293	34,211	,000	,509	34,211	1,000
Error(Tiempo)	Esfericidad asumida	943655,665	66	14297,813					
	Greenhouse-Geisser	943655,665	58,345	16173,619					
	Huynh-Feldt	943655,665	61,403	15368,183					
	Límite-inferior	943655,665	33,000	28595,626					

a. Calculado con alfa = ,05

Comparaciones por pares

Medida:MEASURE_1

(I)Tiempo	(J)Tiempo	Diferencia de medias (I-J)	Error típ.	Sig.[a]	Intervalo de confianza al 95 % para la diferencia[a]	
					Límite inferior	Límite superior
1	2	230,320*	31,875	,000	149,924	310,716
	3	173,243*	31,137	,000	94,709	251,777
2	1	-230,320*	31,875	,000	-310,716	-149,924
	3	-57,077	23,186	,058	-115,558	1,403
3	1	-173,243*	31,137	,000	-251,777	-94,709
	2	57,077	23,186	,058	-1,403	115,558

Basadas en las medias marginales estimadas.

*. La diferencia de medias es significativa al nivel ,05.

a. Ajuste para comparaciones múltiples: Bonferroni.

MAXIMUM FORCE

Factores intra-sujetos

Medida:MEASURE_1

Tiempo	Variable dependiente
1	W_CMJ_Med_Pre_Fmax
2	W_CMJ_min2ymedio_Fmax
3	W_CMJ_Med_Post_Fmax

Estadísticos descriptivos

	Media	Desviación típica	N
W_CMJ_Med_Pre_Fmax	2,5185	,33063	34
W_CMJ_min2ymedio_Fmax	2,3550	,30025	34
W_CMJ_Med_Post_Fmax	2,4406	,25920	34

Contrastes multivariados[c]

Efecto		Valor	F	Gl de la hipótesis	Gl del error	Sig.	Eta al cuadrado parcial	Parámetro de no centralidad Parámetro	Potencia observada[b]
Tiempo	Traza de Pillai	,251	5,375[a]	2,000	32,000	,010	,251	10,750	,806
	Lambda de Wilks	,749	5,375[a]	2,000	32,000	,010	,251	10,750	,806
	Traza de Hotelling	,336	5,375[a]	2,000	32,000	,010	,251	10,750	,806
	Raíz mayor de Roy	,336	5,375[a]	2,000	32,000	,010	,251	10,750	,806

a. Estadístico exacto

b. Calculado con alfa = ,05

c. Diseño: Intersección

Diseño intra-sujetos: Tiempo

Prueba de esfericidad de Mauchly[b]

Medida:MEASURE_1

Efecto intra-sujetos	W de Mauchly	Chi-cuadrado aprox.	gl	Sig.	Epsilon[a]		
					Greenhouse-Geisser	Huynh-Feldt	Límite-inferior
Tiempo	,759	8,829	2	,012	,806	,841	,500

Contrasta la hipótesis nula de que la matriz de covarianza error de las variables dependientes transformadas es proporcional a una matriz identidad.

a. Puede usarse para corregir los grados de libertad en las pruebas de significación promediadas. Las pruebas corregidas se muestran en la tabla Pruebas de los efectos inter-sujetos.

b. Diseño: Intersección

Diseño intra-sujetos: Tiempo

Pruebas de efectos intra-sujetos.

Medida:MEASURE_1

Origen		Suma de cuadrados tipo III	gl	Media cuadrática	F	Sig.	Eta al cuadrado parcial	Parámetro de no centralidad Parámetro	Potencia observada[a]
Tiempo	Esfericidad asumida	,455	2	,227	7,271	,001	,181	14,541	,926
	Greenhouse-Geisser	,455	1,611	,282	7,271	,003	,181	11,716	,879
	Huynh-Feldt	,455	1,682	,270	7,271	,003	,181	12,228	,889
	Límite-inferior	,455	1,000	,455	7,271	,011	,181	7,271	,744
Error(Tiempo)	Esfericidad asumida	2,065	66	,031					
	Greenhouse-Geisser	2,065	53,178	,039					
	Huynh-Feldt	2,065	55,499	,037					
	Límite-inferior	2,065	33,000	,063					

a. Calculado con alfa = ,05

Comparaciones por pares

Medida:MEASURE_1

(I)Tiempo	(J)Tiempo	Diferencia de medias (I-J)	Error típ.	Sig.[a]	Intervalo de confianza al 95 % para la diferencia[a]	
					Límite inferior	Límite superior
1	2	,164*	,051	,009	,035	,292
	3	,078	,032	,062	-,003	,159
2	1	-,164*	,051	,009	-,292	-,035
	3	-,086	,044	,175	-,196	,024
3	1	-,078	,032	,062	-,159	,003
	2	,086	,044	,175	-,024	,196

Basadas en las medias marginales estimadas.

*. La diferencia de medias es significativa al nivel ,05.

a. Ajuste para comparaciones múltiples: Bonferroni.

MAXIMUM TAKEOFF VELOCITY

Factores intra-sujetos

Medida:MEASURE_1

Tiempo	Variable dependiente
1	W_CMJ_Med_Pre_Vmax
2	W_CMJ_min2ymedio_Vmax
3	W_CMJ_Med_Post_Vmax

Estadísticos descriptivos

	Media	Desviación típica	N
W_CMJ_Med_Pre_Vmax	2,8344	,12902	34
W_CMJ_min2ymedio_Vmax	2,7069	,16686	34
W_CMJ_Med_Post_Vmax	2,7455	,16978	34

Contrastes multivariados[c]

Efecto		Valor	F	Gl de la hipótesis	Gl del error	Sig.	Eta al cuadrado parcial	Parámetro de no centralidad Parámetro	Potencia observada[b]
Tiempo	Traza de Pillai	,591	23,150[a]	2,000	32,000	,000	,591	46,299	1,000
	Lambda de Wilks	,409	23,150[a]	2,000	32,000	,000	,591	46,299	1,000
	Traza de Hotelling	1,447	23,150[a]	2,000	32,000	,000	,591	46,299	1,000
	Raíz mayor de Roy	1,447	23,150[a]	2,000	32,000	,000	,591	46,299	1,000

a. Estadístico exacto

b. Calculado con alfa = ,05

c. Diseño: Intersección

Diseño intra-sujetos: Tiempo

Prueba de esfericidad de Mauchly[b]

Medida:MEASURE_1

Efecto intra-sujetos	W de Mauchly	Chi-cuadrado aprox.	gl	Sig.	Epsilon[a]		
					Greenhouse-Geisser	Huynh-Feldt	Límite-inferior
Tiempo	,819	6,400	2	,041	,847	,887	,500

Contrasta la hipótesis nula de que la matriz de covarianza error de las variables dependientes transformadas es proporcional a una matriz identidad.

a. Puede usarse para corregir los grados de libertad en las pruebas de significación promediadas. Las pruebas corregidas se muestran en la tabla Pruebas de los efectos inter-sujetos.

b. Diseño: Intersección

Diseño intra-sujetos: Tiempo

Pruebas de efectos intra-sujetos.

Medida:MEASURE_1

Origen		Suma de cuadrados tipo III	gl	Media cuadrática	F	Sig.	Eta al cuadrado parcial	Parámetro de no centralidad Parámetro	Potencia observada[a]
Tiempo	Esfericidad asumida	,291	2	,145	32,374	,000	,495	64,748	1,000
	Greenhouse-Geisser	,291	1,693	,172	32,374	,000	,495	54,812	1,000
	Huynh-Feldt	,291	1,775	,164	32,374	,000	,495	57,459	1,000
	Límite-inferior	,291	1,000	,291	32,374	,000	,495	32,374	1,000
Error(Tiempo)	Esfericidad asumida	,296	66	,004					
	Greenhouse-Geisser	,296	55,872	,005					
	Huynh-Feldt	,296	58,570	,005					
	Límite-inferior	,296	33,000	,009					

a. Calculado con alfa = ,05

Comparaciones por pares

Medida:MEASURE_1

(I)Tiempo	(J)Tiempo	Diferencia de medias (I-J)	Error típ.	Sig.[a]	Intervalo de confianza al 95 % para la diferencia[a]	
					Límite inferior	Límite superior
1	2	,128*	,018	,000	,081	,174
	3	,089*	,017	,000	,045	,132
2	1	-,128*	,018	,000	-,174	-,081
	3	-,039*	,012	,012	-,070	-,007
3	1	-,089*	,017	,000	-,132	-,045
	2	,039*	,012	,012	,007	,070

Basadas en las medias marginales estimadas.

*. La diferencia de medias es significativa al nivel ,05.

a. Ajuste para comparaciones múltiples: Bonferroni.
