# Supplementary material for: Muscular fatigue in response to different modalities of CrossFit sessions
Source: PLoS One. 2017 Jul 28;12(7):e0181855. doi: 10.1371/journal.pone.0181855 (PMC5533437; doi:10.1371/journal.pone.0181855)
Supplement: S8 File — Statistical analysis performed with mechanical variables in Weight WOD. (PDF) [file pone.0181855.s008.pdf]

TOTAL DURATION JUMP

Factores intra-sujetos

Medida:MEASURE_1

Tiempo	Variable dependiente
1	W_CMJ_Med_Pre__Tiempo_TOTAL
2	W_CMJ_2y5__Tiempo_TOTAL
3	W_CMJ_Med_Post__Tiempo_TOTAL

Estadísticos descriptivos

	Media	Desviación típica	N
W_CMJ_Med_Pre__Tiempo_TOTAL	,7203	,09083	34
W_CMJ_2y5__Tiempo_TOTAL	,7465	,09556	34
W_CMJ_Med_Post__Tiempo_TOTAL	,7431	,09666	34

Contrastes multivariados[c]

Efecto		Valor	F	Gl de la hipótesis	Gl del error	Sig.	Eta al cuadrado parcial	Parámetro de no centralidad Parámetro	Potencia observada[b]
Tiempo	Traza de Pillai	,243	5,139[a]	2,000	32,000	,012	,243	10,278	,787
	Lambda de Wilks	,757	5,139[a]	2,000	32,000	,012	,243	10,278	,787
	Traza de Hotelling	,321	5,139[a]	2,000	32,000	,012	,243	10,278	,787
	Raíz mayor de Roy	,321	5,139[a]	2,000	32,000	,012	,243	10,278	,787

a. Estadístico exacto

b. Calculado con alfa = ,05

c. Diseño: Intersección
Diseño intra-sujetos: Tiempo

Prueba de esfericidad de Mauchly[b]

Medida:MEASURE_1

Efecto intra-sujetos	W de Mauchly	Chi-cuadrado aprox.	gl	Sig.	Epsilon[a]		
					Greenhouse-Geisser	Huynh-Feldt	Límite-inferior
Tiempo	,820	6,344	2	,042	,848	,889	,500

Contrasta la hipótesis nula de que la matriz de covarianza error de las variables dependientes transformadas es proporcional a una matriz identidad.

a. Puede usarse para corregir los grados de libertad en las pruebas de significación promediadas. Las pruebas corregidas se muestran en la tabla Pruebas de los efectos inter-sujetos.

b. Diseño: Intersección
Diseño intra-sujetos: Tiempo

Pruebas de efectos intra-sujetos.

Medida:MEASURE_1

Origen		Suma de cuadrados tipo III	gl	Media cuadrática	F	Sig.	Eta al cuadrado parcial	Parámetro de no centralidad Parámetro	Potencia observada[a]
Tiempo	Esfericidad asumida	,014	2	,007	3,188	,048	,088	6,377	,591
	Greenhouse-Geisser	,014	1,695	,008	3,188	,057	,088	5,405	,542
	Huynh-Feldt	,014	1,777	,008	3,188	,054	,088	5,666	,556
	Límite-inferior	,014	1,000	,014	3,188	,083	,088	3,188	,411
Error(Tiempo)	Esfericidad asumida	,143	66	,002					
	Greenhouse-Geisser	,143	55,940	,003					
	Huynh-Feldt	,143	58,647	,002					
	Límite-inferior	,143	33,000	,004					

a. Calculado con alfa = ,05

Comparaciones por pares

Medida:MEASURE_1

(I)Tiempo	(J)Tiempo	Diferencia de medias (I-J)	Error típ.	Sig.[a]	Intervalo de confianza al 95 % para la diferencia[a]	
					Límite inferior	Límite superior
1	2	-,026*	,009	,014	-,048	-,004
	3	-,023	,012	,191	-,053	,007
2	1	,026*	,009	,014	,004	,048
	3	,003	,013	1,000	-,029	,036
3	1	,023	,012	,191	-,007	,053
	2	-,003	,013	1,000	-,036	,029

Basadas en las medias marginales estimadas.

*. La diferencia de medias es significativa al nivel ,05.

a. Ajuste para comparaciones múltiples: Bonferroni.

ECCENTRIC PHASE

Factores intra-sujetos

Medida:MEASURE_1

Tiempo	Variable dependiente
1	Weight_Pre_Tiempo_Excentrico_Med
2	Weight_2y5_Tiempo_Excentrico
3	Weight_Post_Tiempo_Excentrico_Med

Estadísticos descriptivos

	Media	Desviación típica	N
Weight_Pre_Tiempo_Excentrico_Med	,4892	,07996	34
Weight_2y5_Tiempo_Excentrico	,4790	,07925	34
Weight_Post_Tiempo_Excentrico_Med	,5076	,08927	34

Contrastes multivariados[c]

Efecto		Valor	F	Gl de la hipótesis	Gl del error	Sig.	Eta al cuadrado parcial	Parámetro de no centralidad Parámetro	Potencia observada[b]
Tiempo	Traza de Pillai	,111	2,007[a]	2,000	32,000	,151	,111	4,014	,384
	Lambda de Wilks	,889	2,007[a]	2,000	32,000	,151	,111	4,014	,384
	Traza de Hotelling	,125	2,007[a]	2,000	32,000	,151	,111	4,014	,384
	Raíz mayor de Roy	,125	2,007[a]	2,000	32,000	,151	,111	4,014	,384

a. Estadístico exacto

b. Calculado con alfa = ,05

c. Diseño: Intersección

Diseño intra-sujetos: Tiempo

Prueba de esfericidad de Mauchly[b]

Medida:MEASURE_1

Efecto intra-sujetos	W de Mauchly	Chi-cuadrado aprox.	gl	Sig.	Epsilon[a]		
					Greenhouse-Geisser	Huynh-Feldt	Límite-inferior
Tiempo	,990	,316	2	,854	,990	1,000	,500

Contrasta la hipótesis nula de que la matriz de covarianza error de las variables dependientes transformadas es proporcional a una matriz identidad.

a. Puede usarse para corregir los grados de libertad en las pruebas de significación promediadas. Las pruebas corregidas se muestran en la tabla Pruebas de los efectos inter-sujetos.

b. Diseño: Intersección

Diseño intra-sujetos: Tiempo

Pruebas de efectos intra-sujetos.

Medida:MEASURE_1

Origen		Suma de cuadrados tipo III	gl	Media cuadrática	F	Sig.	Eta al cuadrado parcial	Parámetro de no centralidad Parámetro	Potencia observada[a]
Tiempo	Esfericidad asumida	,014	2	,007	2,020	,141	,058	4,040	,403
	Greenhouse-Geisser	,014	1,981	,007	2,020	,141	,058	4,001	,400
	Huynh-Feldt	,014	2,000	,007	2,020	,141	,058	4,040	,403
	Límite-inferior	,014	1,000	,014	2,020	,165	,058	2,020	,281
Error(Tiempo)	Esfericidad asumida	,234	66	,004					
	Greenhouse-Geisser	,234	65,358	,004					
	Huynh-Feldt	,234	66,000	,004					
	Límite-inferior	,234	33,000	,007					

a. Calculado con alfa = ,05

Comparaciones por pares

Medida:MEASURE_1

(I)Tiempo	(J)Tiempo	Diferencia de medias (I-J)	Error típ.	Sig.[a]	Intervalo de confianza al 95 % para la diferencia[a]	
					Límite inferior	Límite superior
1	2	,010	,014	1,000	-,025	,046
	3	-,018	,015	,695	-,057	,020
2	1	-,010	,014	1,000	-,046	,025
	3	-,029	,014	,152	-,064	,007
3	1	,018	,015	,695	-,020	,057
	2	,029	,014	,152	-,007	,064

Basadas en las medias marginales estimadas.

a. Ajuste para comparaciones múltiples: Bonferroni.

ISOMETRIC PHASE

Factores intra-sujetos

Medida:MEASURE_1

Tiempo	Variable dependiente
1	Weight_Pre_Tiempo_Isometrico_Med
2	Weight_2y5_Tiempo_Isométrico
3	Weight_Post_Tiempo_Isometrico_Med

Estadísticos descriptivos

	Media	Desviación típica	N
Weight_Pre_Tiempo_Isometrico_Med	,0098	,00443	34
Weight_2y5_Tiempo_Isométrico	,0096	,00502	34
Weight_Post_Tiempo_Isometrico_Med	,0160	,02747	34

Contrastes multivariados[c]

Efecto		Valor	F	Gl de la hipótesis	Gl del error	Sig.	Eta al cuadrado parcial	Parámetro de no centralidad Parámetro	Potencia observada[b]
Tiempo	Traza de Pillai	,065	1,109[a]	2,000	32,000	,342	,065	2,218	,228
	Lambda de Wilks	,935	1,109[a]	2,000	32,000	,342	,065	2,218	,228
	Traza de Hotelling	,069	1,109[a]	2,000	32,000	,342	,065	2,218	,228
	Raíz mayor de Roy	,069	1,109[a]	2,000	32,000	,342	,065	2,218	,228

a. Estadístico exacto

b. Calculado con alfa = ,05

c. Diseño: Intersección

Diseño intra-sujetos: Tiempo

Prueba de esfericidad de Mauchly[b]

Medida:MEASURE_1

Efecto intra-sujetos	W de Mauchly	Chi-cuadrado aprox.	gl	Sig.	Epsilon[a]		
					Greenhouse-Geisser	Huynh-Feldt	Límite-inferior
Tiempo	,137	63,628	2	,000	,537	,540	,500

Contrasta la hipótesis nula de que la matriz de covarianza error de las variables dependientes transformadas es proporcional a una matriz identidad.

a. Puede usarse para corregir los grados de libertad en las pruebas de significación promediadas. Las pruebas corregidas se muestran en la tabla Pruebas de los efectos inter-sujetos.

b. Diseño: Intersección

Diseño intra-sujetos: Tiempo

Pruebas de efectos intra-sujetos.

Medida:MEASURE_1

Origen		Suma de cuadrados tipo III	gl	Media cuadrática	F	Sig.	Eta al cuadrado parcial	Parámetro de no centralidad Parámetro	Potencia observada[a]
Tiempo	Esfericidad asumida	,001	2	,000	1,774	,178	,051	3,547	,359
	Greenhouse-Geisser	,001	1,073	,001	1,774	,192	,051	1,904	,261
	Huynh-Feldt	,001	1,081	,001	1,774	,192	,051	1,916	,262
	Límite-inferior	,001	1,000	,001	1,774	,192	,051	1,774	,253
Error(Tiempo)	Esfericidad asumida	,017	66	,000					
	Greenhouse-Geisser	,017	35,425	,000					
	Huynh-Feldt	,017	35,659	,000					
	Límite-inferior	,017	33,000	,001					

a. Calculado con alfa = ,05

Comparaciones por pares

Medida:MEASURE_1

(I)Tiempo	(J)Tiempo	Diferencia de medias (I-J)	Error típ.	Sig.[a]	Intervalo de confianza al 95 % para la diferencia[a]	
					Límite inferior	Límite superior
1	2	,000	,001	1,000	-,003	,003
	3	-,006	,005	,629	-,018	,006
2	1	,000	,001	1,000	-,003	,003
	3	-,006	,004	,490	-,018	,005
3	1	,006	,005	,629	-,006	,018
	2	,006	,004	,490	-,005	,018

Basadas en las medias marginales estimadas.

a. Ajuste para comparaciones múltiples: Bonferroni.

CONCENTRIC PHASE

Factores intra-sujetos

Medida:MEASURE_1

Tiempo	Variable dependiente
1	Weight_Pre_Tiempo_Concentrico_Med
2	Weight_2y5_Tiempo_Concéntrico
3	Weight_Post_Tiempo_Concentrico_Med

Estadísticos descriptivos

	Media	Desviación típica	N
Weight_Pre_Tiempo_Concentrico_Med	,2229	,04254	34
Weight_2y5_Tiempo_Concéntrico	,2579	,06236	34
Weight_Post_Tiempo_Concentrico_Med	,2195	,07060	34

Contrastes multivariados[c]

Efecto		Valor	F	Gl de la hipótesis	Gl del error	Sig.	Eta al cuadrado parcial	Parámetro de no centralidad Parámetro	Potencia observada[b]
Tiempo	Traza de Pillai	,276	6,101[a]	2,000	32,000	,006	,276	12,201	,856
	Lambda de Wilks	,724	6,101[a]	2,000	32,000	,006	,276	12,201	,856
	Traza de Hotelling	,381	6,101[a]	2,000	32,000	,006	,276	12,201	,856
	Raíz mayor de Roy	,381	6,101[a]	2,000	32,000	,006	,276	12,201	,856

a. Estadístico exacto

b. Calculado con alfa = ,05

c. Diseño: Intersección

Diseño intra-sujetos: Tiempo

Prueba de esfericidad de Mauchly[b]

Medida:MEASURE_1

Efecto intra-sujetos	W de Mauchly	Chi-cuadrado aprox.	gl	Sig.	Epsilon[a]		
					Greenhouse-Geisser	Huynh-Feldt	Límite-inferior
Tiempo	,942	1,910	2	,385	,945	1,000	,500

Contrasta la hipótesis nula de que la matriz de covarianza error de las variables dependientes transformadas es proporcional a una matriz identidad.

a. Puede usarse para corregir los grados de libertad en las pruebas de significación promediadas. Las pruebas corregidas se muestran en la tabla Pruebas de los efectos inter-sujetos.

b. Diseño: Intersección

Diseño intra-sujetos: Tiempo

Pruebas de efectos intra-sujetos.

Medida:MEASURE_1

Origen		Suma de cuadrados tipo III	gl	Media cuadrática	F	Sig.	Eta al cuadrado parcial	Parámetro de no centralidad Parámetro	Potencia observada[a]
Tiempo	Esfericidad asumida	,031	2	,015	7,805	,001	,191	15,610	,943
	Greenhouse-Geisser	,031	1,890	,016	7,805	,001	,191	14,755	,933
	Huynh-Feldt	,031	2,000	,015	7,805	,001	,191	15,610	,943
	Límite-inferior	,031	1,000	,031	7,805	,009	,191	7,805	,774
Error(Tiempo)	Esfericidad asumida	,130	66	,002					
	Greenhouse-Geisser	,130	62,386	,002					
	Huynh-Feldt	,130	66,000	,002					
	Límite-inferior	,130	33,000	,004					

a. Calculado con alfa = ,05

Comparaciones por pares

Medida:MEASURE_1

(I)Tiempo	(J)Tiempo	Diferencia de medias (I-J)	Error típ.	Sig.[a]	Intervalo de confianza al 95 % para la diferencia[a]	
					Límite inferior	Límite superior
1	2	-,035*	,011	,011	-,063	-,007
	3	,003	,009	1,000	-,020	,027
2	1	,035*	,011	,011	,007	,063
	3	,038*	,012	,006	,009	,068
3	1	-,003	,009	1,000	-,027	,020
	2	-,038*	,012	,006	-,068	-,009

Basadas en las medias marginales estimadas.

*. La diferencia de medias es significativa al nivel ,05.

a. Ajuste para comparaciones múltiples: Bonferroni.
